# The Learning Rate Is Not a Constant: Sandwich-Adjusted Markov Chain Monte Carlo Simulation

**DOI:** 10.3390/e27100999

**Published:** 2025-09-25

**Authors:** Jasper A. Vrugt, Cees G. H. Diks

**Affiliations:** 1 Department of Civil and Environmental Engineering, University of California, Irvine, CA 92697, USA; 2 Center for Nonlinear Dynamics in Economics and Finance (CeNDEF), Amsterdam School of Economics, University of Amsterdam, 1018 WB Amsterdam, The Netherlands; c.g.h.diks@uva.nl

**Keywords:** maximum likelihood, Bayesian inference, model misspecification, naive variance, sandwich variance, Fisher information, Godambe information, Markov chain Monte Carlo simulation, learning rate, hydrologic modeling, DREAM-Suite

## Abstract

A fundamental limitation of maximum likelihood and Bayesian methods under model misspecification is that the asymptotic covariance matrix of the pseudo-true parameter vector $\theta_{*}$ is not the inverse of the Fisher information, but rather the sandwich covariance matrix 1nA*−1B*−1A*−1, where $A_{*}$ and $B_{*}$ are the sensitivity and variability matrices, respectively, evaluated at $\theta_{*}$ for training data record $\omega_{1},\ldots ,\omega_{n}$. This paper makes three contributions. First, we review existing approaches to robust posterior sampling, including the open-faced sandwich adjustment and magnitude- and curvature-adjusted Markov chain Monte Carlo (MCMC) simulation. Second, we introduce a new sandwich-adjusted MCMC method. Unlike existing approaches that rely on arbitrary matrix square roots, eigendecompositions or a single scaling factor applied uniformly across the parameter space, our method employs a parameter-dependent learning rate λ(θ) that enables direction-specific tempering of the likelihood. This allows the sampler to capture directional asymmetries in the sandwich distribution, particularly under model misspecification or in small-sample regimes, and yields credible regions that remain valid when standard Bayesian inference underestimates uncertainty. Third, we propose information-theoretic diagnostics for quantifying model misspecification, including a strictly proper divergence score and scalar summaries based on the Frobenius norm, Earth mover’s distance, and the Herfindahl index. These principled diagnostics complement residual-based metrics for model evaluation by directly assessing the degree of misalignment between the sensitivity and variability matrices, $A_{*}$ and $B_{*}$. Applications to two parametric distributions and a rainfall-runoff case study with the Xinanjiang watershed model show that conventional Bayesian methods systematically underestimate uncertainty, while the proposed method yields asymptotically valid and robust uncertainty estimates. Together, these findings advocate for sandwich-based adjustments in Bayesian practice and workflows.

## 1. Introduction

Suppose that we have a vector-valued statistical (mathematical) model $y=f\left(\theta \right):R^{d}\to R^{n}$ of a d×1-vector of parameters $\theta ={\left(\theta_{1},\ldots ,\theta_{d}\right)}^{⊤}$ that we wish to estimate from training data $\omega_{n}={\left(\omega_{1},\ldots ,\omega_{n}\right)}^{⊤}$. Common practice is to define a residual loss function $e_{t}\left(\theta \right)=\omega_{t}-y_{t}\left(\theta \right)$ for all t=1,…,n and minimize (maximize, if appropriate) this function using an automatic search algorithm. In this special issue on Bayesian estimation and information theory, we shall use a likelihood function $L_{n}\left(\theta \right)$ for θ given the *n* observations $\omega_{1},\ldots ,\omega_{n}$. However, the problem we address in this paper is not limited to Bayesian methods but applies equally to frequentist inference using least-squares methods. We use $L_{\omega }\left(\theta \right)$ as shorthand notation for L(ω∣θ) and write $L_{\omega }\left(\theta \right)=\log\left(L_{\omega }\left(\theta \right)\right)$ for the log-likelihood function. The joint likelihood $L_{n}\left(\theta \right)$ for the *n*-vector of data points, $\omega_{1},\ldots ,\omega_{n}$, is equal to the product of $L_{\omega_{1}}\left(\theta \right),\ldots ,L_{\omega_{n}}\left(\theta \right)$. Now, the unnormalized *d*-variate posterior density $P_{n}\left(\theta \right)=P\left(\theta |\omega_{n}\right)$ follows from Bayes’ theorem [1], $P_{n}\left(\theta \right)\;\propto \;P\left(\theta \right)L_{n}\left(\theta \right)$, where P(θ) is the prior density. In logarithmic form, $P_{n}\left(\theta \right)=P\left(\theta \right)+L_{n}\left(\theta \right)-\log\left(Z_{n}\right)$, where $P\left(\theta \right)=\log\left(P(\theta )\right)$ is the log-prior and $Z_{n}=\int P\left(\theta \right)L_{n}\left(\theta \right)\;d\theta$ denotes the marginal likelihood.

The Bernstein and von Mises [2] theorem establishes that when the sample size *n* grows, the posterior distribution of the parameters becomes approximately normal, centered on the true parameter values $\theta_{0}$ of the data-generating process and with a covariance matrix $\frac{1}{n}I_{0}^{-1}\left(\theta_{0}\right)$ equal to the inverse of the d×d Fisher [3] information matrix [4]

(1) $$\begin{array}{cc} \; & I_{0}\left(\theta_{0}\right)=E_{\omega }[\nabla L_{\omega }\left(\theta_{0}\right){\left(\nabla L_{\omega }\left(\theta_{0}\right)\right)}^{⊤}]. \end{array}$$

This theorem establishes that Bayesian credible sets asymptotically approximate optimal frequentist confidence sets and, as such, it forms the basis for using Bayesian credible sets in statistical inference. The fundamental underpinning of this theory is the information identity $A_{0}=B_{0}$, where

(2) $$\begin{array}{cc} \; & A_{0}=-E_{\omega }\left[\nabla^{2}L_{\omega }\left(\theta_{0}\right)\right], \end{array}$$

is the so-called sensitivity (negative Hessian) matrix, and

(3) $$\begin{array}{cc} B_{0} & =E_{\omega }[\nabla L_{\omega }\left(\theta_{0}\right){\left(\nabla L_{\omega }\left(\theta_{0}\right)\right)}^{⊤}]\\ \; & ={\mathrm{Var}}_{\omega }\left[\nabla L_{\omega }\left(\theta_{0}\right)\right], \end{array}$$

is the variability matrix at $\theta_{0}$. The term “variability” reflects the well-known identity $\mathrm{Var}\left[X\right]=E[\left(X-\mu \right){\left(X-\mu \right)}^{⊤}]$ with μ=E[X], applied to the score $\nabla L_{\omega }\left(\theta_{0}\right)$

(4) $$\begin{array}{cc} \mathrm{Var}[\nabla L_{\omega }\left(\theta_{0}\right)] & =E_{\omega }\left[\left(\nabla L_{\omega }\left(\theta_{0}\right)-\mu_{0}\right){\left(\nabla L_{\omega }\left(\theta_{0}\right)-\mu_{0}\right)}^{⊤}\right]\\ \; & =E_{\omega }\left[\nabla L_{\omega }\left(\theta_{0}\right){\left(\nabla L_{\omega }\left(\theta_{0}\right)\right)}^{⊤}\right]-\mu_{0}\mu_{0}^{⊤}. \end{array}$$

Under correct specification, the expected score $\mu_{0}=E_{\omega }\left[\nabla L_{\omega }\left(\theta_{0}\right)\right]$, equals a zero vector, and, consequently we yield that $B_{0}={\mathrm{Var}}_{\omega }\left[\nabla L_{\omega }\left(\theta_{0}\right)\right]$.

We then also have that the maximum likelihood (ML) density estimator ${\hat{\theta }}_{n}$ of the posterior parameter distribution satisfies [5]

$$\begin{array}{cc} \sqrt{n}\left({\hat{\theta }}_{n}-\theta_{0}\right) & \overset{d}{⟶}N_{d}\left(0,I_{0}^{-1}\left(\theta_{0}\right)\right), \end{array}$$

where $I_{0}\left(\theta_{0}\right)$ is the expected Fisher information for a single datum. Fisher information plays a fundamental role in statistical inference, including hypothesis testing, regression analysis, and the calculation of standard errors and parameter confidence intervals and regions. The information or second Bartlett [6] identity $A_{0}=B_{0}$, is only valid if the model f(θ) (and hence the likelihood function, $L_{n}\left(\theta \right)$) is correctly specified [7]. If the model is misspecified, the sensitivity and variability matrices are misaligned [8] and asymptotic 100(1−α)% credible intervals will usually have less than nominal frequentist coverage probabilities. Thus, Bayesian credible sets of confidence level γ=100(1−α)% cannot be interpreted as confidence sets of level γ% [9]. This so-called overconditioning [10,11,12] is a result of the customary aleatoric treatment of residuals when, in fact, they are nonrandom (systematic) in nature and manifest with an unduly small parameter uncertainty and poorly calibrated prediction intervals [13,14,15,16,17]. In such cases, interpretation of the posterior parameter distribution $P\left(\theta \right)L_{n}\left(\theta \right)$ may be problematic. Not only can the posterior $P_{n}\left(\theta \right)$ fail to provide a valid probabilistic description of information about θ, but it may also be unclear whether θ corresponds to any meaningful or scientifically relevant quantities [18].

**Figure 1:**
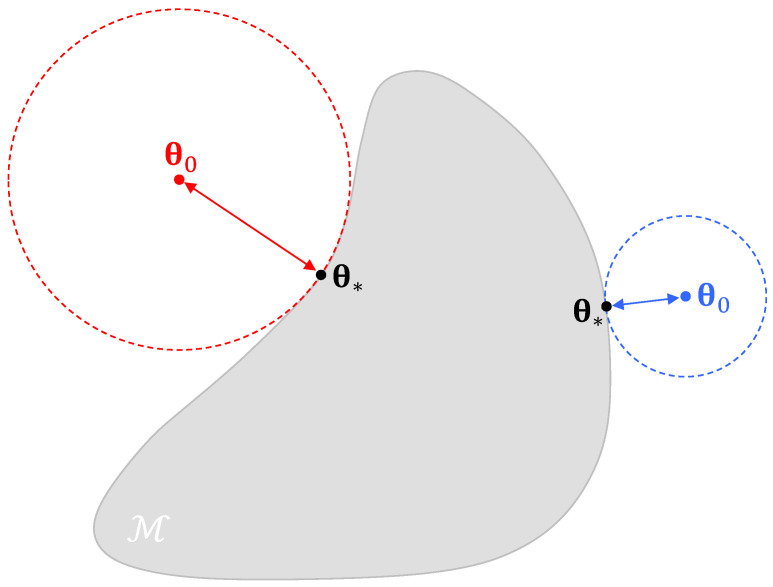
Consequences of model misspecification. Let M denote the family of densities f(ω∣θ) used to model observations $\omega_{1},\ldots ,\omega_{n}$ of a random variable Ω. Suppose the true density $q_{\Omega }\left(\omega |\theta_{0}\right)$ lies outside M (two examples). The *true* parameters $\theta_{0}$ are unattainable and the best approximation is given by the *pseudo-true* parameters $\theta_{*}={\left(\theta_{1,*},\ldots ,\theta_{d,*}\right)}^{⊤}={arg min}_{\theta \;\in \;\Theta }d_{\mathrm{KL}}\left(q_{\Omega }\left(\omega |\theta_{0}\right),f\left(\omega |\theta \right)\right)$.

Upon misspecification, the *true* parameter values $\theta_{0}$ of the data generating process are not in the model parameter space $\theta \in \Theta \subseteq R^{d}$ (see Figure 1).

The best attainable values of the parameters or so-called *pseudo-true* parameter values $\theta_{*}$ minimize the Kullback and Leibler [19] divergence between the *true* probability density function $q_{\Omega }\left(\omega |\theta_{0}\right)$ of Ω and the incorrect family of densities f(ω∣θ) defined by θ∈Θ[8]. The consequences of misspecification are that the posterior distribution will now center on $\theta_{*}$, the best distribution out of all distributions in the misspecified parametric family. However, a more pertinent problem is that the information identity $A_{*}=B_{*}$ will not hold under misspecification. The ML estimator will still be asymptotically normal but now around the pseudo-true parameter values ${\hat{\theta }}_{*}$

$$\begin{array}{cc} \; & \sqrt{n}\left({\hat{\theta }}_{*}\right)\overset{d}{⟶}N_{d}\left(0,G_{0}^{-1}\left(\theta_{*}\right)\right), \end{array}$$

where the covariance matrix of the estimator is 1nA*−1B*−1A*−1=1nG0−1(θ*) and G0=A*−1B*−1A*−1 is the so-called Godambe [20] information matrix. Thus, Godambe information $G_{0}$ is the only valid currency of data information under misspecification. This information matrix guarantees asymptotically valid parameter confidence intervals and standard errors even when the likelihood function $L_{n}\left(\theta \right)$ is incorrectly specified [21].

This paper builds on Vrugt et al. [8] and addresses the fundamental limitation that Bayesian methods do not provide asymptotically valid standard errors when the model is misspecified [22,23,24,25]. The asymptotic covariance matrix of Markov chain Monte Carlo (MCMC) simulation methods is the inverse of a single “slice of bread,” $\frac{1}{n}A_{*}^{-1}$, rather than the asymptotically valid sandwich matrix 1nA*−1B*−1A*−1. Analytic and numerical case studies in Vrugt et al. [8] confirm that the posterior distribution can significantly overestimate the informativeness of streamflow measurements, resulting in an overly optimistic model and parameter uncertainty estimates under misspecification. The sandwich estimator, by contrast, substantially widens the credible intervals for watershed model parameters and discharge. This theoretical inconsistency between Bayesian and frequentist approaches warrants a closer look at MCMC methodology, specifically, how we might adapt the Metropolis–Hastings (MH) algorithm [26,27] so that the stationary distribution of the Markov chains reflects the correct sandwich asymptotics. The general problem we address is that Bayesian methods yield $\theta ∼N_{d}\left({\hat{\theta }}_{*},\frac{1}{n}A_{*}^{-1}\right)$ as the asymptotic description of the posterior parameter distribution $P_{n}\left(\theta \right)=P\left(\theta \right)L_{n}\left(\theta \right)$ or $P_{n}\left(\theta \right)\;\propto \;\exp\left(P_{n}\left(\theta \right)\right)$, whereas this should be θ∼Nd(θ^*,1nA*−1B*−1A*−1) when the model is misspecified. This reconciliation of frequentist asymptotic theory with Bayesian computational procedures is of great practical importance, particularly for applications that make use of prior information, latent variables, and/or hierarchical models. We wish to enhance the robustness of Bayesian computation under model misspecification, while retaining the flexibility and coherence of MCMC simulation methods. We view this not as an attempt to force Bayesian and frequentist methods to align, but as a practical safeguard in applications where model assumptions are inevitably imperfect. Moreover, the strictly proper scoring rules we propose as a byproduct of the misalignment between the sensitivity and variability matrices offer information-theoretically principled metrics for quantifying the degree of misspecification and for guiding model selection and improvement.

The goals of this paper are three-fold. First, we review and examine existing methods for obtaining an asymptotically valid description of the sandwich posterior distribution using MCMC sampling methods. Then, as second objective, we introduce a new and more rigorous sandwich sampling method which overcomes limitations of currently available methods. In particular, existing approaches often rely on a single scalar correction factor applied uniformly across all parameters, which can fail to capture directional asymmetries in the sandwich distribution especially under model misspecification or for small-sample sizes. Our proposed method addresses this limitation by introducing a direction-dependent scaling factor or learning rate that adapts to the local curvature of the sandwich distribution. As third and last objective of this paper, we present an information-theoretic interpretation of the *strictly proper* alignment score proposed by Vrugt et al. [8], which quantifies the concordance between matrices $A_{*}$ and $B_{*}$. Several other scalar indicators of model misspecification are also introduced in this section.

The theory and methodology of this paper are an integral part of DREAM-Suite, a Matlab-Python software package for Bayesian model training, evaluation and diagnostics [28]. This software can be downloaded from the first author’s GitHub account https://github.com/jaspervrugt (accessed on 3 September 2025) and includes the different case studies presented herein.

## 2. Notation and Definitions

Boldface uppercase letters denote matrices, A, boldface lowercase letters signify vectors, a, and italic lowercase letters are used for scalars, *a*. The superscripts “⊤” and “−1” stand for matrix transpose and matrix inverse, respectively. By default, we assume column vectors and, thus, we write $a={\left(a_{1},\ldots ,a_{n}\right)}^{⊤}$ for a n×1 vector. If $X={\left(X_{1},\ldots ,X_{d}\right)}^{⊤}$ is a vector of *d* random variables, then we say that its *expectation* is the vector $\mu ={\left(\mu_{1},\ldots ,\mu_{d}\right)}^{⊤}$ and write μ=E[X], thus combining *d* scalar equations into one vector equation. The *variance* of random vector X is the d×d matrix Σ whose (i,j)th element is

$$\begin{array}{cc} \mathrm{Cov}[X_{i},X_{j}] & =E[\left(X_{i}-\mu_{i}\right)\left(X_{j}-\mu_{j}\right)], \end{array}$$

where i,j∈(1,…,d). In vector notation, we write

$$\begin{array}{cc} \mathrm{Var}[X] & =E[\left(X-\mu \right){\left(X-\mu \right)}^{⊤}], \end{array}$$

thus combining $d^{2}$ scalar equations into one matrix equation. In this formulation, X−μ, is a d×1 vector and the outer (cross) product of X−μ and ${\left(X-\mu \right)}^{⊤}$ returns a d×d matrix. The inner or dot product of two *n*-vectors a and b is equal to $a^{⊤}b$ and returns a scalar. For notational convenience, we write $Z_{n}^{z}\left(\theta \right)$ instead of ${\left(Z_{n}\left(\theta \right)\right)}^{z}$, where the superscript z∈{−1,⊤} denotes either matrix inversion or transposition, respectively. This convention applies to any matrix Z, such as A, B, I, and G. In the same spirit, we write $\nabla_{\theta }^{⊤}L_{\omega }\left(\theta \right)$ to denote the transpose of the gradient vector $\nabla_{\theta }L_{\omega }\left(\theta \right)$, so that outer products are written compactly.

Suppose $\theta_{*}$ are the *pseudo-true* parameter values of the data-generating process S and $\omega ={\left(\omega_{1},\ldots ,\omega_{n}\right)}^{⊤}$ and $y={\left(y_{1},\ldots ,y_{n}\right)}^{⊤}$ are n×1 vectors of materialized and modeled outcomes, respectively. Then, the most important scalars, vectors, and matrices are

1. The likelihood is a scalar and denoted $L_{\omega }\left(\theta \right)$ for a single datum ω. For a data set $\omega_{1},\ldots ,\omega_{n}$, we write $L_{n}\left(\theta \right)$. The symbol $L_{n}\left(\theta \right)$ denotes the natural logarithm of $L_{n}\left(\theta \right)$.
2. The d×d Hessian matrix $H_{\omega }\left(\theta \right)=\nabla^{2}L_{\omega }\left(\theta \right)$ contains the second-order partial derivatives of $L_{\omega }\left(\theta \right)$ w.r.t. θ. The total Hessian is given by $H_{n}\left(\theta \right)=\sum_{i=1}^{n}H_{\omega_{i}}\left(\theta \right)$, equivalently $H_{n}\left(\theta \right)=\nabla^{2}L_{n}\left(\theta \right)$.
3. The d×d sensitivity matrix is defined as $A_{n}=-\frac{1}{n}H_{n}\left({\hat{\theta }}_{*}\right)$ with probability limit $A_{*}=\mathrm{plim}A_{n}$.
4. The d×d variability matrix is defined as $B_{n}=\frac{1}{n}\sum_{i=1}^{n}\nabla L_{\omega_{i}}\left({\hat{\theta }}_{*}\right)\nabla^{⊤}L_{\omega_{i}}\left({\hat{\theta }}_{*}\right)$ with probability limit $B_{*}=\mathrm{plim}B_{n}$.
5. The d×d Fisher information matrix $I_{n}\left(\theta_{*}\right)=E_{\omega }\left[\nabla L_{n}\left(\theta_{*}\right)\nabla^{⊤}L_{n}\left(\theta_{*}\right)\right]$ is the expectation w.r.t. ω of the outer product of the gradient of the log-likelihood evaluated at $\theta_{*}$.
6. The matrix inverse of the Fisher information $I_{n}^{-1}\left(\theta_{*}\right)$ is a d×d covariance matrix. Under *correct specification* this naive variance equals the asymptotic variance of the ML estimator.
7. The d×d Godambe information matrix is defined as Gn(θ^*)=nAn−1Bn−1An−1, with probability limit G0=plim1nGn(θ*)=A*−1B*−1A*−1.
8. The matrix inverse of the Godambe information $G_{n}^{-1}\left({\hat{\theta }}_{*}\right)$ is a d×d covariance matrix. This robust or “sandwich” variance is a consistent estimator of the asymptotic variance of the ML estimator under *misspecification*.

Note that we omitted the subscript θ in the vector differential operator ∇ as differentiation of the log-likelihood function is always with respect to the parameters.

The entries of the d×d “information” matrices $I_{n}$, $H_{n}$, and $G_{n}$ grow linearly (on average) with *n* reflecting a steadily increasing amount of information about the unknown parameters θ with more data. In contrast, $A_{n}$ and $B_{n}$ are sample averages of the sensitivity and variability matrices for *n* data points. Cameron and Trivedi [29] treat these two d×d matrices as estimators of $A_{*}$ and $B_{*}$, respectively, the probability limits under the *pseudo-true* parameters $\theta_{*}$. For the time being, we formulate all our information matrices $A_{*}$, $B_{*}$, $I_{n}\left(\theta_{*}\right)$, and $G_{n}\left(\theta_{*}\right)$ as population quantities as if the *pseudo-true* parameter values $\theta_{*}$ of the data generating process are exactly known. In practice, the “information” matrices $A_{*}$ and $B_{*}$ are replaced with empirical estimates $A_{n}$ and $B_{n}$, evaluated at the estimator ${\hat{\theta }}_{*}$ obtained from the $\omega_{1},\ldots ,\omega_{n}$. Further details are provided later.

Statistical distributions are designated as common symbols. If X is multivariate normally distributed with mean $\mu \in R^{d}$ and d×d covariance matrix Σ=Var[X], we write $X∼N_{d}\left(\mu ,\Sigma \right)$ and use $X∼U_{d}\left(a,b\right)$ for the continuous *d*-variate uniform distribution on the closed-region [a,b], where $a,b\in R^{d\times 1}$ and $a_{j}<b_{j}$ for all j=(1,…,d). We write P(X∣ω) for the conditional pdf of X given the *n*-materialized outcomes ω. The Greek letter α∈(0,1) denotes the probability of rejecting the null hypothesis when the null hypothesis is *true*. We write γ=1−α for the confidence level.

## 3. Illustrative Example

Before discussing how to remedy Bayesian MCMC methods into sampling the asymptotically correct sandwich distribution, we first demonstrate the information identity $A_{*}=B_{*}$ and failure thereof for a simple parametric model and synthetic data.

We revisit the first study of Vrugt et al. [8] and consider as data generating process $\Omega ∼N(\mu ,\sigma^{2})$ of random variable Ω. We draw measurements $\omega_{1},\ldots ,\omega_{n}$ for μ=0, $\sigma^{2}=1$ and n=100. As our model we use $y_{i}∼N\left(m,s^{2}\right)$ with *m* unknown and $s^{2}$ fixed at some predefined value. If $s^{2}=\sigma^{2}$, then the model is correctly specified, otherwise for $s^{2}\neq \sigma^{2}$ the model is misspecified. Now, we wish to determine the value of *m* using training data $\omega_{1},\ldots ,\omega_{100}$. The normal log-likelihood $L_{n}^{n}\left(m|s^{2}\right)$ is equal to

(5) $$\begin{array}{cc} \; & L_{n}^{n}\left(m|s^{2}\right)=\log\left(L_{n}^{n}\left(m|s^{2}\right)\right)=-\frac{n}{2}\log\left(2\pi s^{2}\right)-\frac{1}{2}s^{-2}\sum_{i=1}^{n}{\left(\omega_{i}-m\right)}^{2}. \end{array}$$

**Figure 2:**
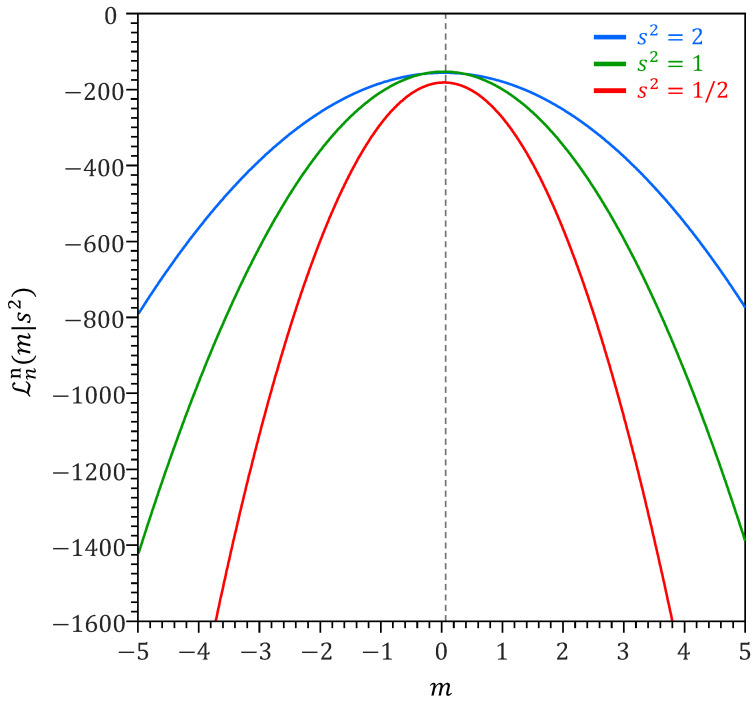
Normal log-likelihood $L_{n}^{n}\left(m|s^{2}\right)$ from Equation (5) as a function of the mean m∈[−5,5] of the Gaussian model $y∼N(m,s^{2})$, shown for three variances: $s^{2}=1/2$, $s^{2}=1$, and $s^{2}=2$. The data $\omega_{1},\ldots ,\omega_{n}$ are sampled from a normal distribution $\Omega ∼N(\mu ,\sigma^{2})$ with μ=0, $\sigma^{2}=1$, and sample size n=100. The vertical dashed gray line indicates the value of *m* that maximizes the log-likelihood.

Figure 2 displays $L_{n}^{n}\left(m|s^{2}\right)$ for −5≤m≤5 using $s^{2}=1/2$ (red), $s^{2}=1$ (green) and $s^{2}=2$ (blue).

For $s^{2}=1$ (green line), the model is correctly specified and the information identity, $A_{*}=B_{*}$ will hold. This implies that the expected value of the second derivative ${\ddot{L}}_{n}^{n}\left(m|s^{2}\right)$ of the log-likelihood function $L_{n}^{n}\left(m|s^{2}\right)$ at the likelihood maximum $\hat{m}\approx \mu$ will equal the expected value of the squared first-derivative ${\dot{L}}_{n}^{n}\left(m|s^{2}\right)$ of $L_{n}^{n}\left(m|s^{2}\right)$ at this maximum, where the expectation is with respect to ω∈Ω. This is easy to demonstrate with an analytic proof. The first and second derivatives of $L_{\omega }^{n}\left(m|s^{2}\right)$ with respect to *m* are

(6) $$\begin{array}{cccc} & \; & {\dot{L}}_{\omega }^{n}\left(m|s^{2}\right) & =\frac{d}{dm}L_{\omega }^{n}\left(m|s^{2}\right)=s^{-2}\left(\omega -m\right)\\ & \; & {\ddot{L}}_{\omega }^{n}\left(m|s^{2}\right) & =\frac{d^{2}}{dm^{2}}L_{\omega }^{n}\left(m|s^{2}\right)=-s^{-2}. \end{array}$$

The sensitivity matrix (a scalar in this case) is now equal to

(7) $$\begin{array}{cc} \; & A_{*}=E_{\omega }\left[-{\ddot{L}}_{\omega }^{n}\left(m|s^{2}\right)\right]=E_{\omega }\left[s^{-2}\right]=s^{-2}, \end{array}$$

and the variability matrix (also a scalar here) at the likelihood maximum m=μ is

(8) $$\begin{array}{cc} \; & B_{*}=E_{\omega }\left[{\dot{L}}_{\omega }^{n}\left(m|s^{2}\right){\dot{L}}_{\omega }^{n}{\left(m|s^{2}\right)}^{⊤}\right]=E_{\omega }\left[s^{-2}\left(\omega -m\right)s^{-2}\left(\omega -m\right)\right]=s^{-4}\sigma^{2}. \end{array}$$

If we assume the variance $s^{2}=\sigma^{2}$ of the green line in Figure 2, then $B_{*}=s^{-2}$. This is equal to the sensitivity matrix $A_{*}=s^{-2}$ from Equation (7), thus $A_{*}=B_{*}$. In this correctly specified case and with variance known, an exact 100(1−α)% confidence interval for $\hat{m}$ is

$$\hat{m}\pm \Phi^{-1}\left(1-\frac{1}{2}\alpha \right)\sqrt{\sigma^{2}/n}.$$

where $\Phi^{-1}\left(p_{\alpha }\right)$ is the quantile function of the standard normal distribution evaluated at percentile $p_{\alpha }=1-\frac{1}{2}\alpha$. This confidence interval for $\hat{m}$ coincides with the classical frequentist interval estimate of the sample mean [30].

For the other two models with $s^{2}=1/2$ (red line) and $s^{2}=2$ (blue line) $A_{*}\neq B_{*}$, and consequently the naive variance $\frac{1}{n}A_{*}^{-1}=s^{2}/n$ will underestimate and overestimate, respectively, the actual uncertainty of *m*. Upon misspecification, the sandwich variance 1nA*−1B*−1A*−1=s2s−4σ2s2/n=σ2/n equals the correct variance ($\sigma^{2}/n)$ of *m*. This estimator does not require prior knowledge of $\sigma^{2}$ as the matrices $A_{*}$ and $B_{*}$ are replaced by their sample estimates, $A_{n}$ and $B_{n}$, evaluated at $\theta ={\hat{\theta }}_{*}$.

In Appendix A, we verify that the variability matrix satisfies the variance rule given in Equation (4), confirming that $B_{*}=\mathrm{Var}\left[{\dot{L}}_{\omega }^{n}\left(m|s^{2}\right)\right]$.

## 4. Sandwich-Adjusted MCMC Simulation

### 4.1. The Metropolis–Hastings Algorithm

We must summarize the posterior parameter distribution, $P_{n}\left(\theta \right)\;\propto \;P\left(\theta \right)L_{n}\left(\theta \right)$. When this task cannot be carried out by analytical means nor by analytical approximation, Monte Carlo simulation methods can be used to generate samples from the posterior distribution.

The basis of MCMC simulation is a Markov chain that generates a random walk through the search space and successively visits solutions with stable frequencies stemming from a stationary distribution, $P_{n}\left(\theta \right)$. Assume that the points $\left\{\theta_{\left(0\right)},\theta_{\left(1\right)},\ldots ,\theta_{\left(t-1\right)}\right\}$ have already been sampled, then the MH algorithm [26,27] proceeds as follows (see Algorithm 1). At iteration *t*, the transition kernel $q(\theta_{p}|\theta_{\left(t-1\right)})$ generates a trial move $\theta_{p}$ around the current chain state $\theta_{\left(t-1\right)}$. Next, this candidate point is accepted with MH probability

(9) $$P_{\mathrm{acc}}\left(\theta_{\left(t-1\right)}\to \theta_{p}\right)=\min\left\{1,\frac{P\left(\theta_{p}\right)L_{n}\left(\theta_{p}\right)q\left(\theta_{\left(t-1\right)}|\theta_{p}\right)}{P\left(\theta_{\left(t-1\right)}\right)L_{n}\left(\theta_{\left(t-1\right)}\right)q\left(\theta_{p}|\theta_{\left(t-1\right)}\right)}\right\},$$

and we set $\theta_{\left(t\right)}=\theta_{p}$, otherwise if the candidate point is rejected, the chain remains at is old position, $\theta_{\left(t\right)}=\theta_{\left(t-1\right)}$. Repeated application of these steps results in a Markov chain $\left\{\theta_{\left(0\right)},\theta_{\left(1\right)},\ldots ,\theta_{\left(T\right)}\right\}$ which, under certain regularity conditions, has a unique stationary distribution with posterior probability density function, $P_{n}\left(\theta \right)$. In practice, this means that if one looks at the values of θ sufficiently far from the arbitrary initial value, $\theta_{\left(0\right)}$, the successively generated states of the chain will be distributed according to $P_{n}\left(\theta \right)$, the posterior probability distribution of θ. This so-called burn-in period $\left\{\theta_{\left(0\right)},\theta_{\left(1\right)},\ldots ,\theta_{\left(b-1\right)}\right\}$, where b≪T is required to allow the chain to travel to the high-probability density (HPD) region of the target distribution. Thus, the last M=T−b+1 samples $\left\{\theta_{\left(b\right)},\theta_{\left(b+1\right)},\ldots ,\theta_{\left(T\right)}\right\}$ are used to approximate the posterior parameter distribution, $P_{n}\left(\theta \right)\;\propto \;P\left(\theta \right)L_{n}\left(\theta \right)$.
**Algorithm 1** Metropolis–Hastings (MH)**Input:** Prior, P(θ), likelihood, $L_{n}\left(\theta \right)$, and transition density, $q(\theta_{p}|\theta_{\left(t-1\right)})$        Total number of samples *T***Output:** Samples $\left\{\theta_{\left(0\right)},\theta_{\left(1\right)},\ldots ,\theta_{\left(T\right)}\right\}$ with stationary distribution $P_{n}\left(\theta \right)\;\propto \;P\left(\theta \right)L_{n}\left(\theta \right)$Draw initial chain state $\theta_{\left(0\right)}$ from the prior distribution, $\theta_{\left(0\right)}∼P\left(\theta \right)$**for** t=1 **to** *T* **do**    Sample a proposal, $\theta_{p}∼q\left(\;\cdot \;|\theta_{\left(t-1\right)}\right)$, from the transition kernel    Compute the acceptance probability $P_{\mathrm{acc}}\left(\theta_{\left(t-1\right)}\to \theta_{p}\right)$ using Equation (9)    **if** $P_{\mathrm{acc}}\left(\theta_{\left(t-1\right)}\to \theta_{p}\right)\geq Z$ **then**        Accept the candidate point, $\theta_{\left(t\right)}=\theta_{p}$ and $P_{n}\left(\theta_{\left(t\right)}\right)=P_{n}\left(\theta_{p}\right)$    **else**        Reject the proposal and set $\theta_{\left(t\right)}=\theta_{\left(t-1\right)}$ and $P_{n}\left(\theta_{\left(t\right)}\right)=P_{n}\left(\theta_{\left(t-1\right)}\right)$    **end if****end for****Return:** $\left\{\theta_{\left(0\right)},\theta_{\left(1\right)},\ldots ,\theta_{\left(T\right)}\right\}$

In the limit of T→∞, the MAP density estimate ${\hat{\theta }}_{n}$ of the sampled Markov chain will converge to the *true* parameter values $\theta_{0}$ of the data generating process

$$\begin{array}{cc} \; & \lim_{n,T\;\to \;\infty }{\hat{\theta }}_{n}=\theta_{0}, \end{array}$$

or under misspecification, we write $\lim_{n,T\;\to \;\infty }{\hat{\theta }}_{n}=\theta_{*}$. The asymptotic covariance matrix of the Markov chain will equal the matrix inverse of a single “slice of bread”

$$\begin{array}{cc} \; & \lim_{n,T\;\to \;\infty }n\mathrm{Cov}\left[\left\{\theta_{\left(b\right)},\theta_{\left(b+1\right)},\ldots ,\theta_{\left(T\right)}\right\}\right]=A_{*}^{-1}. \end{array}$$

Thus, after burn-in, the covariance matrix of the chain draws is the nonlinear sample equivalent of $\frac{1}{n}A_{*}^{-1}$, whereas we desire this to be

limn,T→∞nCov[{θ(b),θ(b+1),…,θ(T)}]=A*−1B*−1A*−1.

In the language of Shaby [25], we want to complete the sandwich by joining the slice of bread $A_{*}^{-1}$ to the open-faced sandwich B*−1A*−1 to obtain the desired sandwich covariance.

It would be ideal if we could reformulate the recipe of Algorithm 1 so that the sampled Markov chains always converge to the right asymptotic distribution, which under misspecification is the sandwich estimator. This has proven to be a formidable task. The culprit is the implicit assumption in Bayes law that the model correctly describes the relationship between prior, likelihood, and evidence. We can relax this assumption with the use of so-called belief distributions, but it is not immediately clear how to turn this new paradigm into an MH recipe with correct limiting distribution under misspecification.

We do not delve into the MCMC theory, but rather focus our attention on more practical remedies that help adjust the random walk of Algorithm 1 to the sandwich distribution. Existing methods for doing so transform either the likelihood function $L_{n}\left(\theta \right)$ or the parameter values θ to match the curvature of the sandwich distribution around $\theta_{*}$. All these methods assume knowledge of the MAP parameter values ${\hat{\theta }}_{*}$ and the sensitivity $A_{*}$ and variability $B_{*}$ matrices at $\theta =\theta_{*}$. Next, we review two existing recipes based on magnitude- and curvature-adjustments of the log-likelihood function to sample the posterior sandwich distribution. Then, we present the theory of a third, more rigorous, and convenient approach, which we coin the *kernel-amendment* method for sandwich-adjusted MCMC simulation.

### 4.2. Algorithm 1: Magnitude Adjustment

We can adjust the magnitude of the log-likelihood function to enforce the sandwich variance matrix $\Sigma_{n}^{\mathrm{sand}}$ on the posterior realizations of the sampled Markov chain(s). If the log-likelihood function $L_{n}\left(\theta \right)$ is approximately quadratic in a neighborhood of the maximum a posteriori (MAP) parameter values ${\hat{\theta }}_{*}$, then posterior exploration of the scaled log-likelihood $kL_{n}\left(\theta \right)$ via the MH algorithm should yield a good approximation to the sandwich-adjusted posterior. This so-called *omnibus* adjustment was originally proposed by Pauli et al. [31] as a correction to the second Bartlett identity, and the scalar *k* is estimated following the procedures outlined by Ribatet et al. [24] and di San Miniato and Sartori [32]

(10) k=dtr(Σnnaive)−1Σnsand⟶pdtr(A*−1B*−1).

The unary trace operator tr(·) returns the sum of the diagonal elements of the d×d matrix A*−1B*−1. This trace is equal to the sum of the eigenvalues of the matrix-matrix product A*−1B*−1. The *omnibus* adjustment can be thought of as a tempering of the log-likelihood function and flattens $L_{n}\left(\theta \right)$ for 0<k<1, thereby slowing down learning and matching the informativeness of the data with the Godambe information Gn(θ*)=nA*−1B*−1A*−1. The MH algorithm with product of the *omnibus* scalar *k* and log-likelihood $L_{n}\left(\theta \right)$, will from now on be referred to as Algorithm 1.

**Remark 1.**  The omnibus scalar k is reminiscent of a learning rate in a power likelihood function, where ’learning rate’ refers to the extent to which the data influence posterior updating. A value of k<1 downweights the data and reduces sensitivity to outliers, while k>1 increases the influence of the data on the posterior distribution. To provide a deeper intuition of the advantages of a power likelihood, we revisit example 1 in Section 3. Suppose that we inadvertently assumed that $s^{2}=2$ (blue line in Figure 2) and the normal distribution model is misspecified. Our estimate of the (naive) variance of $\hat{m}$ will be $\frac{1}{n}A_{*}^{-1}=s^{2}/n=2/n$, while the true variance of $\hat{m}$ is $\sigma^{2}/n$ or 1/n. With the omnibus scalar, the sensitivity and variability matrices of $kL_{n}^{n}\left(m|s^{2}\right)$ are equal to $A_{*}=ks^{-2}$ and $B_{*}=k^{2}s^{-4}\sigma^{2}$, respectively (see Appendix C). For $s^{2}=2$, we have $A_{*}=k/2$ and $B_{*}=k^{2}/4$. The information identity $A_{*}=B_{*}$ holds if $k^{2}-2k=0$, yielding, k=2. At this value of k, the naive variance of $kL_{n}^{n}\left(\theta \right)$ is $\frac{1}{n}A_{*}^{-1}=2k^{-1}/n$ or 1/n, which is the correct variance of $\hat{m}$ as $\sigma^{2}=1$. Thus, the idea behind the omnibus scalar k is to choose its value such that the powered likelihood $L_{n}^{k}\left(\theta \right)$ satisfies the information identity $A_{*}=B_{*}$. As a result, the naive posterior parameter distribution under $L_{n}^{k}\left(\theta \right)$ coincides with the sandwich distribution of the original likelihood $L_{n}\left(\theta \right)$. This omnibus correction yields the correct variance-covariance matrix for the estimator ${\hat{\theta }}_{*}$.

**Remark 2.**  The use of a single scalar k for all d parameters may suffice when the sandwich distribution is well approximated by a multivariate Gaussian, that is, when the posterior is nearly symmetric and its surface is approximately quadratic around ${\hat{\theta }}_{*}$. However, in the presence of model misspecification, prior truncation, or directional heterogeneity in sensitivity, a global scalar k will distort the shape of the adjusted posterior distribution. In such cases, a separate scaling factor is required for each dimension to preserve the local geometry and asymmetry of the sandwich distribution. This directional asymmetry becomes especially pronounced in small-sample settings, say, n<100, where the central limit approximation does not hold and curvature varies across parameters.

**Remark 3.**  *Other definitions of the scalar k have been proposed in the literature [33], including those based on moment-matching conditions [34], adjustments to degrees of freedom [35] inspired by the Satterthwaite–Welch method [36,37], and alternative scaling approaches [38]. See also Varin et al. [39] for a broader overview.*

**Remark 4.**  *The exponent in a power likelihood is typically denoted by λ rather than k.*

### 4.3. Algorithm 2: Curvature Adjustment

While the asymptotic covariance of ${\hat{\theta }}_{*}$ is the sandwich matrix $\Sigma_{n}^{\mathrm{sand}}$, the MH algorithm instead yields $\frac{1}{n}A_{*}^{-1}$, the inverse of a single “slice of bread”. In the words of Shaby [25], we wish to complete the sandwich by attaching this slice to the open-faced piece B*−1A*−1 to recover the full sandwich covariance 1nA*−1B*−1A*−1. We now review two approaches that adjust the curvature of the log-likelihood to match this target distribution.

#### 4.3.1. A-Posteriori Adjustment

Let $\left\{\theta_{\left(b\right)},\theta_{\left(b+1\right)},\ldots ,\theta_{\left(T\right)}\right\}$ denote the post-burn-in samples from a Markov chain of length *T*, where the first *b* realizations are discarded. This yields M=T−b+1 samples drawn from the naive posterior $P_{n}\left(\theta \right)\;\propto \;P\left(\theta \right)L_{n}\left(\theta \right)$ based on a data sample of size *n*. We center these samples at a reference point ${\hat{\theta }}_{*}$ (e.g., posterior mode or mean) and pre-multiply by a d×d matrix $\Psi_{*}$ to obtain open-face sandwich (OFS)-adjusted samples [25]

(11) $$\begin{array}{cc} \theta_{\left(j\right)}^{\mathrm{ofs}} & ={\hat{\theta }}_{*}+\Psi_{*}\left(\theta_{\left(j\right)}-{\hat{\theta }}_{*}\right),\;j=1,\ldots ,M. \end{array}$$

This linear map applies direction-specific dilations along the principal axes of the local posterior ellipsoid without unnecessary rotation [38], thereby changing the local geometry of the naive posterior to that of the sandwich distribution. A convenient choice is [25]

(12) Ψ*⊤=A*−11/2B*1/2A*1/2.

Under standard regularity conditions, the naive posterior is locally Gaussian with covariance $\frac{1}{n}A_{*}^{-1}$. After centering at ${\hat{\theta }}_{*}$ and applying the transformation $\Psi_{*}$, we obtain

Cov(θofs)=CovΨ*⊤(θ−θ^*)=Ψ*⊤1nA*−1Ψ*⊤=1nA*−11/2B*1/2A*1/2A*−11/2A*1/2B*1/2A*−11/2=1nA*−1B*−1A*−1,

which is precisely the asymptotic sandwich covariance. Here, $A_{*}^{1/2}$ is the principal square root of the symmetric positive definite matrix $A_{*}$ such that $A_{*}^{1/2}\;\cdot \;A_{*}^{1/2}=A_{*}$.

This a posteriori correction method is computationally appealing, as it does not require additional evaluations of the likelihood function $L_{n}\left(\theta \right)$. However, the OFS-adjusted posterior samples may not accurately represent the true sandwich parameter distribution, particularly if the adjustment matrix $\Psi_{*}$ is not constant over the region of ${\hat{\theta }}_{*}$ in which there is sandwich parameter uncertainty. However, this assumption is not unique to curvature-based methods. What is specific to such methods is the nonuniqueness of the matrix square roots $A_{*}^{1/2}$ and $B_{*}^{1/2}$ when the sensitivity and variability matrices $A_{*}$ and $B_{*}$ are not positive semi-definite. This can occur if the quadratic approximation of $L_{n}\left(\theta \right)$ via the second-order Taylor expansion at $\theta ={\hat{\theta }}_{*}$ does not adequately describe the actual curvature of the log-likelihood function. In such cases, $A_{*}$ and $B_{*}$ may not be symmetric. Moreover, under certain conditions, these matrices may be ill-conditioned or nearly singular. This may arise when two or more parameters exhibit strong linear dependence, when parameters have very different magnitudes, and/or when the sample size *n* is small. Ill-conditioning can also be introduced as an artifact of numerical approximation, particularly when finite differences are used to estimate the first- and second-order partial derivatives of $L_{n}\left(\theta \right)$ with respect to θ. Floating-point arithmetic can lead to numerical instability due to rounding errors and subtractive cancellation when evaluating small differences between almost equal numbers. This will distort the computation of $A_{*}$ and $B_{*}$.

We can symmetrize a matrix Z by working with $\frac{1}{2}\left(Z+Z^{⊤}\right)$ instead. Ill-conditioning can be addressed through Tikhonov regularization by adding to $A_{*}$ and/or $B_{*}$ a diagonal matrix, $\epsilon I_{d}$, where $I_{d}$ is the d×d identity matrix and ϵ>0 is a small positive scalar. This technique, also known as ridge regression, changes the eigenvalues of the matrix from ${\underline{\lambda }}_{1},\ldots ,{\underline{\lambda }}_{d}$ to ${\underline{\lambda }}_{1}+\epsilon ,\ldots ,{\underline{\lambda }}_{d}+\epsilon$. If all eigenvalues are positive and the matrix is symmetric, then its principal square roots will be unique. Throughout this paper, we assume that the matrices $A_{*}$ and $B_{*}$ are positive definite.

The matrix square roots $A_{*}^{1/2}$ and $B_{*}^{1/2}$ of can be computed using different methods, including the generalized Cholesky factorization [40,41], singular value decomposition [42] or eigendecomposition [43,44]. These methods yield similar matrix square roots when $A_{*}$ and $B_{*}$ are approximately symmetric and positive definite, since the key properties of symmetric positive definite matrices—such as real, positive eigenvalues and diagonalizability—still hold approximately in such cases. In this case, Cholesky factorization provides computationally inexpensive and stable estimates of $A_{*}^{1/2}$ and $B_{*}^{1/2}$[45]. However, if the matrices $A_{*}$ and $B_{*}$ are far from symmetric, we should expect different matrix square roots, as the methods listed above have different optimality and invariance properties [46]. Preferably, the transformation from $\theta_{\left(j\right)}$ to $\theta_{\left(j\right)}^{\mathrm{ofs}}$ preserves directions of asymmetry. Singular value decomposition is numerically stable and preserves key geometric attributes of $A_{*}$ and $B_{*}$ [25]. For symmetric matrices, the matrix square roots are given by

(13) A*1/2=Ua1/2⊤Da1/2Ua1/2⊤andB*1/2=Ub1/2⊤Db1/2Ub1/2⊤,

where Ua⊤Da⊤Ua⊤ and Ub⊤Db⊤Ub⊤ are the eigendecompositions of the sensitivity and variability matrices, respectively. Here, $U_{a}$ and $U_{b}$ are orthogonal matrices whose columns are the eigenvectors of $A_{*}$ and $B_{*}$, and $D^{1/2}=\mathrm{diag}\left(\sqrt{{\underline{\lambda }}_{1}},\ldots ,\sqrt{{\underline{\lambda }}_{d}}\right)$, is the diagonal matrix of square roots of the corresponding eigenvalues.

The OFS adjustment of Equation (11) is equivalent to a linear transformation of the posterior samples. This transformation may not yield an accurate description of the *true* sandwich distribution if the model is highly nonlinear and/or the parameters are highly correlated. This difference between linear and nonlinear confidence intervals is well understood for the naive variance estimator [14,47,48,49,50], and these findings also apply to the sandwich estimator. In this situation, a priori adjustment (discussed next) will help determine whether the *true* sandwich confidence regions extend beyond this linear approximation. A consistent estimator of $\Psi_{*}$ should generate credible intervals that are consistent 100(1−α)% confidence intervals.

#### 4.3.2. A Priori Adjustment

The OFS adjustment (Method 2a) transforms posterior samples post hoc, after the MH algorithm has completed sampling. While computationally appealing, this approach does not guarantee an accurate characterization of the *true* sandwich distribution under model misspecification. An alternative is to adjust the curvature of the likelihood function $L_{n}\left(\theta \right)$ near the point estimate ${\hat{\theta }}_{*}$, thereby preserving the correct asymptotic behavior. Ribatet et al. [24] proposed achieving this by applying the affine transformation of Equation (11) during MCMC simulation

(14) $$\begin{array}{cc} \theta_{p}^{\mathrm{ca}} & ={\hat{\theta }}_{*}+C_{*}\left(\theta_{p}-{\hat{\theta }}_{*}\right). \end{array}$$

The likelihood $L_{n}\left(\theta \right)$ is then evaluated at the curvature-adjusted candidate point $\theta_{p}^{\mathrm{ca}}$ instead of $\theta_{p}$, resulting in the curvature-adjusted likelihood function

(15) $$\begin{array}{cc} L_{n}^{\mathrm{ca}}\left(\theta \right) & =L_{n}\left(\theta^{\mathrm{ca}}\right)=L_{n}\left({\hat{\theta }}_{*}+C_{*}\left(\theta_{p}-{\hat{\theta }}_{*}\right)\right). \end{array}$$

The transformation in (14) does not change the location of the MAP solution. Indeed, if we enter ${\hat{\theta }}_{*}$ we yield that $\theta_{p}^{\mathrm{ca}}={\hat{\theta }}_{*}$, and, thus, the MAP solution is also a global maximum for $L_{n}^{\mathrm{ca}}\left(\theta \right)$. The gradient or score and curvature (Hessian) of the curvature-adjusted log-likelihood $L_{n}^{\mathrm{ca}}\left(\theta \right)$ at θ is equal to

∇Lnca(θ)=C*⊤∇Ln(θca)and∇2Lnca(θ)=C*⊤∇2Ln(θca)C*⊤.

At the MAP estimator, the Hessian of the original log-likelihood $L_{n}\left(\hat{\theta }\right)$ is asymptotically $\nabla^{2}L_{n}\left({\hat{\theta }}_{*}\right)\approx -nA_{*}$, so that

∇2Lnca(θ^*)=−nC*⊤A*⊤C*⊤.

To obtain the correct asymptotic curvature under model misspecification, we equate this expression to the sandwich information matrix

(16) C*⊤A*⊤C*⊤=A*−1B*−1A*−1.

If $A_{*}$ is symmetric positive definite, both its square root $A_{*}^{1/2}$ and inverse square root $A_{*}^{-1/2}$ exist. Multiplying both sides of Equation (16) on the left and right by $A_{*}^{-1/2}$ gives

A*−1/2C*⊤−1/2A*−1/2C*−1/2A*−1/2=A*−1/2A*−1/2B*−1−1/2A*−1/2A*−1/2.

Letting Q=A*1/2C*−1/2A*−1/2 and hence C*−1/2=A*−1/2QA*1/2, the above expression simplifies to

Q⊤Q=A*1/2B*−11/2A*1/2.

The matrix Q is not unique, since any matrix of the form RQ, where R is an orthogonal matrix ($R^{⊤}R=I_{d}$), also satisfies the same condition. This phenomenon is known as *rotational freedom*, and it implies that any matrix square root $Q_{r}$ is only defined up to an orthogonal rotation or reflection

Qr=RQ=RA*1/2B*−11/2A*1/21/2.

Substituting this expression for $Q_{r}$ back into the expression for $C_{*}$ yields the general form of the d×d curvature-adjustment matrix from Equation (14)

(17) C*=A*−1/2RA*1/2B*−11/2A*1/21/2A*1/2.

This derivation demonstrates the nonuniqueness of matrix $C_{*}$. If $A_{*}$ and $B_{*}$ commute (e.g., are simultaneously diagonalizable), the expression for $C_{*}$ in Equation (17) simplifies to the following more compact form [24,32,38]

(18) C*−1=A*−1/2G01/2=A*−1/2A*1/2B*−1/2A*1/2=B*−1/2A*1/2,

and enforces the sandwich covariance matrix 1nA*−1B*−1A*−1 onto the sampled Markov chains.

The mapping from θ to $\theta^{\mathrm{ca}}$ can be regarded as a succession of transformations in which the ellipsoidal contours of $L_{n}^{\mathrm{ca}}\left(\theta \right)$ are first mapped to spheroids, and then transformed back to the contours of $L_{n}\left(\theta \right)$[38]. The MH acceptance probability of candidate point $\theta_{p}$ becomes

(19) Pacc(θ(t−1)→θp)=min1,P(θp∘)Ln(θpca)q(θ(t−1)∣θp∘)P(θ(t−1)∘)Ln(θ(t−1)ca)q(θp∣θ(t−1)∘).

Thus, the comparison of candidate points $\theta_{p}$ and the current chain state $\theta_{\left(t-1\right)}$ in curvature-adjusted MCMC simulation takes place after $\theta_{p}$ and $\theta_{\left(t-1\right)}$ are scaled and rotated [25]. Ribatet et al. [24] build on a result from Kent [51] to show that the acceptance probability in (19) shares the same asymptotic distribution as the *true* likelihood ratio. They further argue that the resulting sample has an asymptotic stationary distribution that is normal, with the desired sandwich covariance matrix.

Algorithm 2 outlines the steps of curvature-adjusted MCMC simulation using the MH algorithm. We refer to this procedure as the Curvature-Adjusted Metropolis–Hastings (CAMH) algorithm. The Markov chain generated by the CAMH algorithm has an asymptotic stationary distribution that is *d*-variate normal with mean ${\hat{\theta }}_{*}$ and d×d sandwich covariance matrix given by 1nA*−1B*−1A*−1. For a symmetric proposal distribution, $q\left(\theta_{\left(t-1\right)}|\theta_{p}\right)=q\left(\theta_{p}|\theta_{\left(t-1\right)}\right)$, and Equation (19) simplifies to

Pacc(θ(t−1)→θp)=min1,P(θp∘)Ln(θpca)P(θ(t−1)∘)Ln(θ(t−1)ca).

The above expression further reduces to a likelihood ratio with a uniform prior, $P\left(\theta \right)=U_{d}\left(\theta^{\min},\theta^{\max}\right)$, where $\theta^{\min}$ and $\theta^{\max}$ are *d*-vectors with lower and upper bounds of the parameters, where $\theta_{j}^{\min}<\theta_{j}^{\max}$ for all j=1,…,d.

The CAMH algorithm facilitates exploration of the posterior sandwich distribution, though it presents certain implementation challenges. First, and foremost, the tuning matrix $C_{*}$ may not be uniquely defined when the log-likelihood $L_{n}\left(\theta \right)$ in ${\hat{\theta }}_{*}$ is not exactly quadratic. In such cases, the nonuniqueness of the matrix square roots $A_{*}^{1/2}$ and $B_{*}^{1/2}$ introduces an arbitrary rotation of the spheroids prior to the back-transformation. Although this rotation is inconsequential when the likelihood is locally quadratic around ${\hat{\theta }}_{*}$, care must be taken to ensure that the mapping preserves directions of asymmetry in the posterior sandwich distribution. This issue is not specific to CAMH; any method that relies on the square roots of the “information” matrices $A_{*}$ and $B_{*}$ is subject to this ambiguity.

Second, a subtler issue arises from the nature of the curvature adjustment itself. The transformation is affine and acts on the parameter values such that the likelihood at a point θ is evaluated at its transformed counterpart $\theta^{\mathrm{ca}}$, that is, $L_{n}\left(\theta^{\mathrm{ca}}\right)$. This approach may be easy to implement, but is not intuitive. It may assign high likelihoods to points that are relatively far from the MAP estimate ${\hat{\theta }}_{*}$, even when their original likelihoods were comparatively low, and vice versa. For example, if $\theta_{m}$ is a local maximum of $L_{n}\left(\theta \right)$, the transformation substitutes $L_{n}\left(\theta_{m}\right)$ with $L_{n}\left({\hat{\theta }}_{*}+C_{*}\left(\theta_{m}-{\hat{\theta }}_{*}\right)\right)$, regardless of the actual likelihood at $\theta_{m}$. As a result, asymmetries or non-elliptical features of $L_{n}\left(\theta \right)$ can be distorted, compromising the geometric fidelity of the sandwich distribution.

Finally, the parameter transformation used in curvature adjustment can conflict with bounded parameter spaces. A candidate point $\theta_{p}$ may satisfy the prior constraints in the original parameterization, yet its curvature-adjusted counterpart $\theta_{p}^{\mathrm{ca}}$ may lie outside the feasible parameter space. This complication is not insurmountable, but requires careful handling to ensure that the Markov chain respects parameter constraints, preserves detailed balance, and maintains acceptable sampling efficiency.
**Algorithm 2** Curvature-adjusted Metropolis–Hastings (CAMH)**Input:** Prior, P(θ), likelihood, $L_{n}\left(\theta \right)$, and transition density, $q(\theta_{p}|\theta_{\left(t-1\right)})$   Total number of samples *T*   MAP solution, ${\hat{\theta }}_{*}$, and d×d tuning matrix $C_{*}$ of Equation (18)**Output:** Samples $\left\{\theta_{\left(0\right)},\theta_{\left(1\right)},\ldots ,\theta_{\left(T\right)}\right\}$ from sandwich posterior of $P_{n}\left(\theta \right)\;\propto \;P\left(\theta \right)L_{n}\left(\theta \right)$Draw initial chain state $\theta_{\left(0\right)}$ from the prior distribution, $\theta_{\left(0\right)}∼P\left(\theta \right)$Transform initial state, $\theta_{\left(0\right)}^{\mathrm{ca}}={\hat{\theta }}_{*}+C_{*}\left(\theta_{\left(0\right)}-{\hat{\theta }}_{*}\right)$, and compute $L_{n}\left(\theta_{\left(0\right)}^{\mathrm{ca}}\right)$**for** t=1 **to** *T* **do**    Sample a proposal $\theta_{p}∼q\left(\;\cdot \;|\theta_{\left(t-1\right)}\right)$ from the transition kernel    Transform the candidate point $\theta_{p}^{\mathrm{ca}}={\hat{\theta }}_{*}+C_{*}\left(\theta_{p}-{\hat{\theta }}_{*}\right)$ and compute $L_{n}\left(\theta_{p}^{\mathrm{ca}}\right)$    Compute the acceptance probability $P_{\mathrm{acc}}\left(\theta_{\left(t-1\right)}\to \theta_{p}\right)$ using Equation (19)    Draw a label *Z* from a uniform distribution, Z∼U(0,1)    **if** $P_{\mathrm{acc}}\left(\theta_{\left(t-1\right)}\to \theta_{p}\right)\geq Z$ **then**   Accept the candidate point, $\theta_{\left(t\right)}=\theta_{p}$ and $P_{n}\left(\theta_{\left(t\right)}\right)=P_{n}\left(\theta_{p}\right)$    **else**   Reject the proposal and set $\theta_{\left(t\right)}=\theta_{\left(t-1\right)}$ and $P_{n}\left(\theta_{\left(t\right)}\right)=P_{n}\left(\theta_{\left(t-1\right)}\right)$    **end if****end for****Return:** $\left\{\theta_{\left(0\right)},\theta_{\left(1\right)},\ldots ,\theta_{\left(T\right)}\right\}$

### 4.4. Algorithm 3: Kernel Adjustment

Given the limitations of existing sampling methods, we propose a new approach, so-called kernel-amendment, which combines elements of magnitude- and curvature-adjusted MCMC simulation but introduces two key innovations, (i) a direction-dependent scaling factor that captures asymmetric and non-quadratic features of the sandwich distribution, and (ii) an implementation that avoids matrix square roots of $A_{*}$ and $B_{*}$. This method guarantees an accurate description of the sandwich distribution by MCMC methods. We first develop the theoretical framework, then assess the performance of both kernel-amendment and existing MCMC sampling methods through applications to commonly used parametric distributions and to numerical models, using both synthetic and measured data.

#### Theory

Suppose $P_{n}\left(\theta \right)\;\propto \;P\left(\theta \right)L_{n}\left(\theta \right)$ is the unnormalized posterior density, or in logarithmic form, $P_{n}\left(\theta \right)\;\propto \;P\left(\theta \right)+L_{n}\left(\theta \right)$. Our proposed solution is to sample from the density

(20) $$\begin{array}{cc} \phi (\theta ) & \;\propto \;\exp\left\{\lambda \left(\theta \right)\left(L_{n}\left(\theta \right)-L_{n}\left({\hat{\theta }}_{*}\right)\right)\right\}, \end{array}$$

where $\lambda \left(\theta \right):R^{d}\to R$ is a scalar-valued function which scales linearly the difference in the log-likelihoods of points θ and ${\hat{\theta }}_{*}$. The argument of the exponential

(21) $$\begin{array}{cc} L_{n}^{p}\left(\theta |\lambda \right) & =\log\left(L_{n}^{p}\left(\theta |\lambda \right)\right)=\lambda \left(\theta \right)\left[L_{n}\left(\theta \right)-L_{n}\left({\hat{\theta }}_{*}\right)\right], \end{array}$$

is itself a power log-likelihood function. By construction, $L_{n}^{p}\left({\hat{\theta }}_{*}|\lambda \right)=0$, while for all other $\theta \in \Theta \subseteq R^{d}$, it is negative. Subtracting the value at ${\hat{\theta }}_{*}$ recenters $L_{n}^{p}\left(\theta |\lambda \right)$ so that its Hessian $H_{n}\left({\hat{\theta }}_{*}\right)=\nabla^{2}L_{n}^{p}\left({\hat{\theta }}_{*}|\lambda \right)$ accurately captures the local curvature free from arbitrary offsets in $L_{n}\left(\theta \right)$. This centering allows us to exactly align the curvature of the power log-likelihood function with that of the sandwich distribution, even when $L_{n}\left(\theta \right)$ is asymmetric near the mode and eliminates the need for matrix square roots (as shown later). Choosing ${\hat{\theta }}_{*}$ as the centering point ensures generality: under a uniform prior it coincides with the ML estimator, while under an informative prior it becomes the MAP estimator. This makes the approach seamlessly applicable to both frequentist and Bayesian settings. In short, subtracting, $L_{n}\left({\hat{\theta }}_{*}\right)$ normalizes curvature for scaling and avoids contamination by arbitrary log-likelihood offsets.

The Metropolis acceptance probability for a candidate point $\theta_{p}$ now becomes

(22) $$\begin{array}{cc} P_{\mathrm{acc}}\left(\theta_{p}\to \theta_{\left(t-1\right)}\right) & =\min\left\{1,\frac{P\left(\theta_{p}\right)L_{n}^{p}\left(\theta_{p}|\lambda \right)q\left(\theta_{\left(t-1\right)}|\theta_{p}\right)}{P\left(\theta_{\left(t-1\right)}\right)L_{n}^{p}\left(\theta_{\left(t-1\right)}|\lambda \right)q\left(\theta_{p}|\theta_{\left(t-1\right)}\right)}\right\}, \end{array}$$

where $L_{n}^{p}\left(\theta |\lambda \right)$ is the normalized power likelihood function

(23) $$\begin{array}{cc} L_{n}^{p}\left(\theta |\lambda \right)=\exp\left(L_{n}^{p}\left(\theta |\lambda \right)\right) & =\exp\left(\lambda \left(\theta \right)[L_{n}\left(\theta \right)-L_{n}\left({\hat{\theta }}_{*}\right)]\right)\\ \; & =\exp\left(\lambda \left(\theta \right)\log\left(L_{n}\left(\theta \right)\right)-\lambda \left(\theta \right)\log\left(L_{n}\left({\hat{\theta }}_{*}\right)\right)\right)\\ \; & =\exp\left(\log\left(L_{n}{\left(\theta \right)}^{\lambda (\theta )}\right)-\log\left(L_{n}{\left({\hat{\theta }}_{*}\right)}^{\lambda (\theta )}\right)\right)\\ \; & ={\left(\frac{L_{n}\left(\theta \right)}{L_{n}\left({\hat{\theta }}_{*}\right)}\right)}^{\;\lambda (\theta )}. \end{array}$$

The acceptance probability in Equation (22) reduces to a likelihood ratio

$$\begin{array}{cc} P_{\mathrm{acc}}\left(\theta_{p}\to \theta_{\left(t-1\right)}\right) & =\min\{1,L_{n}^{p}\left(\theta_{p}|\lambda \right)/L_{n}^{p}\left(\theta_{\left(t-1\right)}|\lambda \right)\}\\ \; & =\min\left\{1,\frac{L_{n}{\left({\hat{\theta }}_{*}\right)}^{\lambda (\theta_{\left(t-1\right)})}}{L_{n}{\left({\hat{\theta }}_{*}\right)}^{\lambda (\theta_{p})}}\frac{L_{n}{\left(\theta_{p}\right)}^{\lambda (\theta_{p})}}{L_{n}{\left(\theta_{\left(t-1\right)}\right)}^{\lambda (\theta_{\left(t-1\right)})}}\right\}\\ \; & =\min\left\{1,\frac{L_{n}{\left(\theta_{p}\right)}^{\lambda (\theta_{p})}}{L_{n}{\left(\theta_{\left(t-1\right)}\right)}^{\lambda (\theta_{\left(t-1\right)})}}L_{n}{\left({\hat{\theta }}_{*}\right)}^{\left(\lambda \left(\theta_{\left(t-1\right)}\right)-\lambda \left(\theta_{p}\right)\right)}\right\}, \end{array}$$

in the case of a uniform prior and symmetric transition kernel of the Markov chain.

We are now left to discuss the choice of λ(θ). What should this scalar-valued function be? For θ in the vicinity of ${\hat{\theta }}_{*}$ we know that

$$\begin{array}{cc} L_{n}\left(\theta \right)-L_{n}\left({\hat{\theta }}_{*}\right) & \approx -\frac{1}{2}n{\left(\theta -{\hat{\theta }}_{*}\right)}^{⊤}A_{*}\left(\theta -{\hat{\theta }}_{*}\right), \end{array}$$

whereas we desire this to be

Ln(θ)−Ln(θ^*)=−12n(θ−θ^*)⊤A*−1B*−1A*−1(θ−θ^*).

So a sensible choice for λ(θ) might be

(24) λ(θ)=(θ−θ^*)⊤A*−1B*−1A*−1(θ−θ^*)(θ−θ^*)⊤A*(θ−θ^*).

With this formulation for λ(θ), the acceptance probability $P_{\mathrm{acc}}\left(\theta_{p}\to \theta_{\left(t-1\right)}\right)$ of Equation (22) will guide a Markov chain to a stationary distribution with the underlying probability density function ϕ(θ) in Equation (20). A formal proof of this result is provided in Appendix B, and the complete recipe for the sandwich-adjusted Metropolis–Hastings (SAMH) algorithm is given in Algorithm 3. By multiplying the difference in the log-likelihoods between any point θ and the MAP solution ${\hat{\theta }}_{*}$ by the learning rate λ(θ), the resulting Markov chain converges to a stationary distribution with the correct asymptotic sandwich variance.
**Algorithm 3** Sandwich-adjusted Metropolis–Hastings (SAMH)**Input:** Prior, P(θ), likelihood, $L_{n}\left(\theta \right)$, and transition density, $q(\theta_{p}|\theta_{\left(t-1\right)})$   Total number of samples *T*   MAP solution, ${\hat{\theta }}_{*}$, associated likelihood, $L_{n}\left({\hat{\theta }}_{*}\right)$, and matrices $A_{*}$ and $B_{*}$**Output:** Samples $\left\{\theta_{\left(0\right)},\theta_{\left(1\right)},\ldots ,\theta_{\left(T\right)}\right\}$ from sandwich posterior of $P_{n}\left(\theta \right)\;\propto \;P\left(\theta \right)L_{n}\left(\theta \right)$Draw initial chain state $\theta_{\left(0\right)}$ from the prior distribution, $\theta_{\left(0\right)}∼P\left(\theta \right)$Compute $\lambda (\theta_{\left(0\right)})$ in Equation (24) and $L_{n}^{p}\left(\theta_{\left(0\right)}|\lambda \right)$ of Equation (21)**for** t=1 **to** *T* **do**    Sample a proposal $\theta_{p}∼q\left(\;\cdot \;|\theta_{\left(t-1\right)}\right)$ from the transition density    Compute $\lambda (\theta_{p})$ in Equation (24) and $L_{n}^{p}\left(\theta_{p}|\lambda \right)$ in Equation (21)    Compute the acceptance probability, $P_{\mathrm{acc}}\left(\theta_{\left(t-1\right)}\to \theta_{p}\right)$, using Equation (22)    Draw a label *Z* from a uniform distribution, Z∼U(0,1)    **if** $P_{\mathrm{acc}}\left(\theta_{\left(t-1\right)}\to \theta_{p}\right)\geq Z$ **then**   Accept the candidate point, $\theta_{\left(t\right)}=\theta_{p}$ and $P_{n}\left(\theta_{\left(t\right)}\right)=P_{n}\left(\theta_{p}\right)$    **else**   Reject the proposal and set $\theta_{\left(t\right)}=\theta_{\left(t-1\right)}$ and $P_{n}\left(\theta_{\left(t\right)}\right)=P_{n}\left(\theta_{\left(t-1\right)}\right)$    **end if****end for****Return:** $\left\{\theta_{\left(0\right)},\theta_{\left(1\right)},\ldots ,\theta_{\left(T\right)}\right\}$

Before proceeding with a detailed discussion of our methodology, we briefly revisit our expression for λ(θ). According to Definition 7 in Section 2 on Page 5, we can express the numerator of Equation (24) in terms of $G_{0}$, the expected Godambe information of a single observation

(25) $$\begin{array}{cc} \lambda (\theta ) & =\frac{{\left(\theta -{\hat{\theta }}_{*}\right)}^{⊤}G_{0}\left(\theta -{\hat{\theta }}_{*}\right)}{{\left(\theta -{\hat{\theta }}_{*}\right)}^{⊤}A_{*}\left(\theta -{\hat{\theta }}_{*}\right)}. \end{array}$$

Matrix $A_{*}$ in the denominator is itself an information matrix. Under correct model specification, this matrix equals $I_{0}$, the expected Fisher information of a single datum. The scalar λ(θ) is thus the ratio of two information matrices, with $A_{*}$ reflecting sensitivity-based curvature and $G_{0}\left(\theta \right)$ representing the Godambe (sandwich) information. In most practical cases of model misspecification, the latter has smaller curvature, so 0<λ(θ)≤1, with equality at 1 under correct specification. Values λ(θ)>1 can occur but are uncommon, arising when the variability-based curvature locally exceeds the sensitivity-based curvature. In all cases, λ(θ) acts as a parameter-dependent learning rate that tempers data informativeness, ensuring that Algorithm 3 converges to the sandwich rather than the naive posterior distribution. The nonnegative multiplier λ(θ) can also be viewed as a kernel: it is symmetric about ${\hat{\theta }}_{*}$ and positive semi-definite due to the information matrices in both numerator and denominator. In keeping with the terminology of the other two methods, we refer to our approach as the kernel-adjustment method, though the term generalized power likelihood is also appropriate.

**Remark 5.**  We could define ϕ(θ)=exp−12n(θ−θ^*)⊤A*−1B*−1A*−1(θ−θ^*) as the probability density function of the sandwich posterior distribution [52]. This mathematical form is equivalent to a d-variate normal distribution Nd(θ^*,1nA*−1B*−1A*−1) centered on ${\hat{\theta }}_{*}$ with d×d sandwich covariance matrix. We can draw any desired number of “posterior” samples from this distribution. However, this assumes that the local quadratic sandwich approximation is valid. Algorithm 3 relaxes this assumption for finite n. The rationale for choosing a multiplicative form—rather than, say, an additive one—will become evident soon when we interpret our method in terms of power likelihoods.

**Remark 6.**  For the single-parameter case, matrices $A_{*}$ and $B_{*}$ are scalars, and $\lambda =A_{*}/B_{*}$ can be interpreted as an estimator of the “best” λ in power likelihood, $\exp(\lambda L_{n}\left(\theta \right))$, in the sense of having correctly sized credible sets asymptotically. In other words, for d=1, our approach reduces to the magnitude adjustment method (Method 1), but with one important distinction. We apply the power λ to the score difference $L_{n}\left(\theta \right)-L_{n}\left({\hat{\theta }}_{*}\right)$ rather than to the log-likelihood $L_{n}\left(\theta \right)$ itself, as in Ribatet et al. [24]. This centering of the power likelihood around $L_{n}\left({\hat{\theta }}_{*}\right)$ ensures proper scaling for d>1.

**Remark 7.**  The ratio λ(θ) in Equation (24) depends only on the direction of $\theta -{\hat{\theta }}_{*}$, not on its magnitude. In fact, we obtain λ(θ)=λ(rθ) for any scalar r≠0. Although λ(θ) is not defined at $\theta ={\hat{\theta }}_{*}$, the product $\lambda \left(\theta \right)[L_{n}\left(\theta \right)-L_{n}\left({\hat{\theta }}_{*}\right)]$ remains well defined. This is because both the denominator of Equation (24) and the log-likelihood difference $L_{n}\left(\theta \right)-L_{n}\left({\hat{\theta }}_{*}\right)$ exhibit similar quadratic behavior in a neighborhood of $\theta ={\hat{\theta }}_{*}$. Thus, Algorithm 3 generalizes the power likelihood to the multi-parameter case d>1 as

$$P_{n}\left(\theta \right)\;\propto \;\exp\left(\lambda \left(\theta \right)\left(L_{n}\left(\theta \right)-L_{n}\left({\hat{\theta }}_{*}\right)\right)\right),$$

*where λ(θ)=λ(rθ) for any scalar r≠0, thus defining a distinct scaling factor for each direction in parameter space.*

**Remark 8.**  The kernel λ(θ) has an eigenspace decomposition. We can transform the d×1-vector of differences $\theta -{\hat{\theta }}_{*}$ and write lambda as a function of the matrix-vector product $\vartheta =A_{*}^{1/2}\left(\theta -{\hat{\theta }}_{*}\right)$. The resulting expression

$$\lambda_{\vartheta }\left(\vartheta \right)=\frac{\vartheta^{⊤}A_{*}^{1/2}B_{*}^{-1}A_{*}^{1/2}\vartheta }{\vartheta^{⊤}\vartheta },$$

is a Rayleigh quotient in terms of ϑ. The largest and smallest value of the Rayleigh quotient are equal to the largest ${\underline{\lambda }}_{1}$ and smallest ${\underline{\lambda }}_{d}$ eigenvalues of the precision matrix M≡A*1/2−1B*−11/2A*1/2−1.

**Remark 9.**  *Since M is the inverse variance-covariance matrix of the posterior of ϑ, the critical values of $\lambda_{\vartheta }\left(\vartheta \right)$ where the gradient vanishes correspond to the orthogonal eigenvectors $\vartheta_{1}\leq \ldots \leq \vartheta_{d}$ of M.*

**Remark 10.**  Eigenvalues and eigenvectors of M are informative on how much information the data carry on average on the transformed pseudo-true parameter values $\vartheta_{*}=A_{*}^{1/2}\left(\theta_{*}-\hat{\theta }\right)$ in each of the d eigendirections of ϑ relative to what you would expect under correct specification.

### 4.5. Other Methods

As a general remedy for poor uncertainty quantification under misspecification, Frazier et al. [53] replace the usual posterior with a score-based approximate posterior

P˜n(θ)∝P(θ)exp−n2s¯n⊤(θ)B^n−1(θ)s¯n⊤(θ),

where ${\bar{s}}_{n}\left(\theta \right)=\frac{1}{n}\sum_{i=1}^{n}s_{\omega_{i}}\left(\theta \right)$ is the d×1 mean score with $s_{\omega_{i}}\left(\theta \right)=\nabla L_{\omega_{i}}\left(\theta \right)$, and ${\hat{B}}_{n}\left(\theta \right)$ estimates the score variability. This works well as n→∞, but with a flat prior the exponential kernel equals 1 at any θ where ${\bar{s}}_{n}\left(\theta \right)=0_{d}$, so all such roots attain the same peak height. This obscures the relative support of competing modes and distorts the faithful representation of multimodal posteriors. These issues are most acute at small sample sizes.

In a recent paper, Li and Rice [54] reviewed Bayesian analogues of sandwich variance estimators and derive Bayes rules under a so-called balanced inference loss function, $\ell_{\mathrm{BI}}\left(\theta \right)$. Such loss functions, originally introduced by Zellner [55] and discussed by Dawid and Sebastiani [56] in the context of Bayesian decision theory and optimal experimental design, blend attributes of standard parametric inference with weighted average penalty terms for lack-of-fit and estimation error

(26) $$\begin{array}{cc} \ell_{\mathrm{BI}}\left(\theta ,\Sigma_{n},\Phi \right) & =\log(|\Sigma_{n}\left|\right)+\underset{\mathrm{Estimation}\;\mathrm{error}}{\underset{︸}{{\left(\theta -\mu_{\theta }\right)}^{⊤}\Phi \Sigma_{n}^{-1}\left(\theta -\mu_{\theta }\right)}}\;+\underset{\mathrm{Lack}\;\mathrm{of}\;\mathrm{fit}}{\underset{︸}{\frac{1}{n}\sum_{i=1}^{n}\nabla^{⊤}L_{\omega_{i}}\left(\theta \right){\left\{\Phi A_{n}\left(\theta \right)\right\}}^{-1}\nabla L_{\omega_{i}}\left(\theta \right)}}, \end{array}$$

where $\Sigma_{n}$ signifies the n×n measurement error covariance matrix of the data, $\omega_{1},\ldots ,\omega_{n}$, $\mu_{\theta }$ is the d×1 vector of expected parameter values for the *n* data points, and Φ is a d×d positive definite weighting matrix. This balanced inference loss function is equivalent to a negative log-likelihood function in a Bayesian context. Li and Rice [54] show by simulation that the balanced inference loss function yields robust Bayesian standard error estimates under model misspecification, thus retaining the attractive features of frequentist inference. Yet, the balanced loss function of Equation (26) is optimal only when residuals follow a Gaussian distribution. This is a significant limitation for discharge residuals of conceptual hydrologic models, which typically deviate from normality and are more accurately described by Laplacian or double-exponential distributions [11,57]. Moreover, the balanced loss function requires repeated evaluation of the sensitivity matrix $A_{n}$—i.e., the empirical Fisher information, which incurs a substantial computational overhead on the order of $d^{2}$ model evaluations for each MH candidate point $\theta_{p}$. The balanced inference loss function is well-suited for the ABC model used in our first sandwich paper [8], as it provides an analytic expression for the sensitivity matrix $A_{n}$. For other studies, we resort instead to magnitude-, curvature-, or sandwich-adjusted MCMC simulation.

## 5. Empirical Estimates of Information Matrices

Sandwich-adjusted MCMC simulation assumes knowledge of the *true* parameter values $\theta_{0}$ (and $\theta_{*}$ under misspecification) and the sensitivity and variability matrices $A_{*}$ and $B_{*}$, respectively, of the data-generating process S. These are theoretical quantities that are not known in practice. In Section 2, we define that $A_{*}$ is the probability limit of $A_{n}$ and, similarly, $B_{*}=\mathrm{plim}B_{n}$, where $A_{n}$ and $B_{n}$ are the averages of the sensitivity and variability matrices for the *n* data points $\omega_{1},\ldots ,\omega_{n}$. Thus, we must replace the population quantities $A_{*}$ and $B_{*}$ with their sample-based estimates

$$A_{n}=-P_{{\hat{\theta }}_{*}}^{n}\;\nabla^{2}L_{\omega }\left(\theta \right)\;\mathrm{and}\;B_{n}=P_{{\hat{\theta }}_{*}}^{n}\;\nabla L_{\omega }\left(\theta \right)\nabla_{\theta }^{⊤}L_{\omega }\left(\theta \right),$$

where the notation with a precursor $P_{{\hat{\theta }}_{*}}^{n}$ is borrowed from Kleijn and van der Vaart [9] and designates that we must evaluate the *n*-sample average for $\omega_{1},\ldots ,\omega_{n}$ of the quantity to its right and in the MAP solution $\theta ={\hat{\theta }}_{*}$. Sample-based quantities exhibit variability due to the random nature of the data, but should be consistent estimates. That means that as the sample size *n* increases, $A_{n}$ and $B_{n}$ tend to converge in probability to $A_{*}$ and $B_{*}$, respectively. The matrices $C_{*}$ and $\Psi_{*}$ used in a priori and a posteriori curvature-adjusted posterior exploration, respectively, are replaced by their sample equivalents

$$C_{n}=B_{n}^{-1/2}A_{n}^{1/2}\;\mathrm{and}\;\Psi_{n}=A_{n}^{-1}B_{n}^{1/2}A_{n}^{1/2},$$

where the matrix square roots $A_{n}^{1/2}$ and $B_{n}^{1/2}$ follow from Equation (13) using singular value decomposition.

The MAP solution can be determined from an optimization method or a MCMC pre-trial. The Markov chain sample that maximizes the posterior density, $P_{n}\left(\theta \right)=P\left(\theta \right)L_{n}\left(\theta \right)$, is a MAP estimator. Next, the d×1 vector of first-order derivatives, $\nabla L_{\omega }\left(\theta_{*}\right)$, and d×d matrix of second-order derivatives, $\nabla^{2}L_{\omega }\left(\theta_{*}\right)$, can be determined by numerical means using values of the log-likelihood function $L_{n}\left(\theta \right)$ at points nearby ${\hat{\theta }}_{*}$. We use the DERIVESTsuite toolbox of D’Errico [58], a Matlab collection of fully adaptive numerical differentiation methods for scalar- and vector-valued functions. This toolbox handles the computation of first- and higher-order derivatives of functions that do not have simple analytical expressions. We employ semi-adaptive central difference schemes of varying orders, combined with a generalized Richardson [59] extrapolation approach (this method is also referred to as multi-term extrapolation in the context of numerical integration Romberg [60]), to enhance the accuracy of the first- and second-order partial derivatives of the log-likelihood function $L_{n}\left(\theta \right)$ w.r.t. the parameters. This estimation is obtained using a sequence of logarithmically spaced points away from the MAP solution. The “best” differencing interval is automatically selected from the sequence of proportionally cascading points to minimize the approximation errors of $\nabla L_{n}\left(\theta_{*}\right)$ and $\nabla^{2}L_{n}\left(\theta_{*}\right)$.

If successive data points $\omega_{1},\ldots ,\omega_{n}$ exhibit serial correlation, then we must correct the variability matrix $B_{n}$ for possible autocorrelation among the successive scores, $\nabla L_{\omega_{1}}\left({\hat{\theta }}_{*}\right),\ldots ,\nabla L_{\omega_{n}}\left({\hat{\theta }}_{*}\right)$. As in Vrugt et al. [8], we use the estimator of Newey and West [61] to determine the variability matrix $\beta_{n}$ of the scores $g_{t}=\nabla L_{\omega_{t}}\left({\hat{\theta }}_{*}\right)$ as follows

(27) $$\begin{array}{cc} \beta_{n} & =B_{0}+\sum_{\tau =1}^{\tau_{\max}}w\left(\tau ,\tau_{\max}\right)\left(B_{\tau }+B_{\tau }^{⊤}\right), \end{array}$$

where

$$\begin{array}{cc} B_{\tau } & =\frac{1}{n}\sum_{t=\tau +1}^{n}g_{t}g_{t-\tau }^{⊤}, \end{array}$$

is an estimate of the autocovariance matrix of scores a distance τ apart, $\tau_{\max}\in N_{+}$ signifies the maximum lag and

$$\begin{array}{cc} w(\tau ,\tau_{\max}) & =1-\frac{\tau }{1+\tau_{\max}}\;\tau \in \left[0,\tau_{\max}\right], \end{array}$$

is a weight function which smooths the sample autocovariance function [62]. For τ=0, we yield $B_{0}=\frac{1}{n}\sum_{t=1}^{n}g_{t}g_{t}^{⊤}$, which corresponds to $B_{n}$, the variance matrix of the scores—provided that the scores have zero mean. Under correct model specification, the sum of the lagged autocovariance matrices, $B_{\tau }$ vanishes, yielding a d×d zero matrix. Consequently, we have $\beta_{n}=B_{n}=B_{0}$.

Bartlett [63] proposed truncating the sum in Equation (27) at a finite lag $\tau_{\max}$ so as to balance the trade-off between estimator variance and bias. This finite lag is also called the Bartlett window. Larger windows increase the estimator’s variance, whereas smaller values of $\tau_{\max}$ increase the bias of $\beta_{n}$ by omitting relevant score autocovariances. Bartlett’s ideas about the adequate choice of the truncation lag $\tau_{\max}$ have been formalized in rules of thumb such as $\tau_{\max}=\left\lfloor c\;\cdot \;n^{1/4}\right\rfloor$ or $\tau_{\max}=c\left\lfloor 4{\left(n/100\right)}^{2/9}\right\rfloor$ [64], where *c* is a small positive integer and the function ⌊·⌋ rounds down to the nearest integer. If the scores exhibit strong autocorrelation, one can set *c* relatively large, say c=10, otherwise, one may use c=1. We set c=5 and yield values of $\tau_{\max}$ on the order of 12, 18, 32, and 55 for data sets of length n=10, n=100, n=1000, and n=10,000, respectively.

## 6. Case Studies

We demonstrate the different sampling methods by application to three different case studies with increasing complexity. The first two case studies involve statistical models and analytic differentiation. These two studies are purposely kept simple, as this allows us to clearly demonstrate the effects of model misspecification and illustrate how the sandwich estimator rectifies resulting biases in uncertainty quantification as a result of a wrong model parameterization (study 1) or an inadequate parametric form (study 2) for the data-generating process. The third and last study considers the application of the presented methods to rainfall-discharge simulation using the Xinanjiang model [65,66]. This study confirms that traditional MCMC methods produce overly narrow credible regions, so-called overconditioning, and demonstrates the advantages of our proposed SAMH algorithm (Algorithm 3) for sandwich-adjusted posterior exploration.

For MCMC simulation, we employ the DREAM_(ZS)_ algorithm [67,68,69], a differential evolution-based sampler that evolves multiple chains in parallel. Candidate points are generated dynamically using linear combinations of differences between chain states. The transition kernel is self-adaptive, automatically adjusting to the scale and orientation of the target distribution, $P_{n}\left(\theta \right)\;\propto \;P\left(\theta \right)L_{n}\left(\theta \right)$. Computational efficiency is not a primary concern in this paper, as our main objective is to evaluate the theoretical and practical differences between the magnitude-, curvature-, and sandwich-adjusted MCMC simulation methods. The relative speed with which an MCMC method converges to the sandwich distribution does not influence the validity of these adjustments, which are the central focus of our work. Nonetheless, the DREAM algorithm has been benchmarked extensively and shown to perform well across a wide range of complex inference problems (see references in Vrugt [28]). To ensure reliable posterior exploration, we monitor convergence using a suite of established diagnostics, including the single-chain methods of Raftery and Lewis [70] and Geweke [71], as well as the multi-chain scale-reduction factors proposed by Gelman and Rubin [72] and Brooks and Gelman [73], following best practices advocated by Cowles and Carlin [74].

### 6.1. Case Study 1

We revisit example 1 in Section 3 and compute the ML estimate $\hat{m}$ of the mean of the normal distribution model $N(m,s^{2})$, and the corresponding values of $A_{n}$, $B_{n}$, *omnibus* scalar $\hat{k}=B_{n}^{-1}A_{n}$, naive $\Sigma_{n}^{\mathrm{naive}}=\frac{1}{n}A_{n}^{-1}$, and sandwich $\Sigma_{n}^{\mathrm{sand}}=\frac{1}{n}A_{n}^{-1}B_{n}A_{n}^{-1}$ variances using $s^{2}=2$, $s^{2}=1$ and $s^{2}=1/2$. The ML solution $\hat{m}$ is simply equal to the sample mean of the data points $\omega_{1},\ldots ,\omega_{100}$ drawn from $N(\mu ,\sigma^{2})$ with μ=0 and $\sigma^{2}=1$ and $A_{n}$ and $B_{n}$ are derived by numerical means using the DERIVESTsuite toolbox of D’Errico [58]. We repeat this computation for $M=10^{4}$ different realizations of the *n* data points. Table 1 presents the result of this Monte Carlo experiment and lists mean values of $\hat{m}$, $A_{n}$, $B_{n}$, $\hat{k}$, $\Sigma_{n}^{\mathrm{naive}}$ and $\Sigma_{n}^{\mathrm{sand}}$ and their respective standard deviations (in parentheses). The Matlab code is given in Appendix C.

The tabulated results confirm the theory. The ML estimate of the mean $\hat{m}$ is centered around zero and has a standard deviation that approaches the theoretic standard deviation $\sqrt{\sigma^{2}/n}=0.1$. The ML sensitivity matrix $A_{n}$ derived from numerical differentiation equals its theoretic value $A_{*}=s^{-2}$ and does not differ between the trials. The variability matrix $B_{n}$ approaches its theoretic value $B_{*}=s^{-4}\sigma^{2}$ and has a nonzero standard deviation as $\sigma^{2}$ is replaced by the sample variance of the ω’s. The mean of the omnibus scalar approaches its theoretic value $k=B_{*}^{-1}A_{*}=s^{4}\sigma^{-2}s^{-2}$. Thus, we find that $k=s^{2}\sigma^{-2}$ and the standard deviation between parenthesis is a result that $\sigma^{2}$ of the data-generating process is replaced by the sample variance of the ω’s. The naive variance estimator is equal to its theoretic value $\Sigma_{n}^{\mathrm{naive}}=s^{2}/n$ and does not differ between the trials, as *n* and $s^{2}$ are fixed. The sandwich variance estimator $\Sigma_{n}^{\mathrm{sand}}$ does not depend on the value of $s^{2}$ and asymptotically converges to the *true* variance of the mean μ of the data-generating process. The standard deviation is the result of the variation in $B_{n}$ in the Monte Carlo trials.

**Figure 3:**
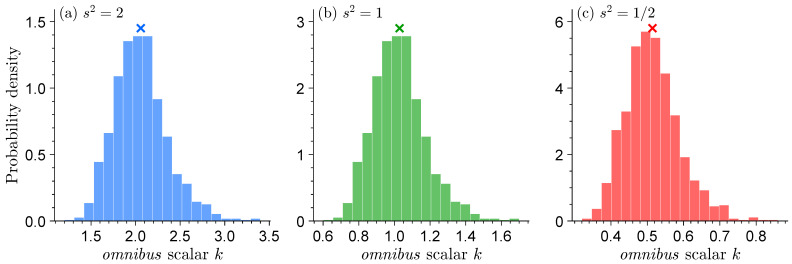
Histogram of the *omnibus* scalar *k* for the $M=10^{4}$ Monte Carlo simulations using (**a**) $s^{2}=2$, (**b**) $s^{2}=1$ and (**c**) $s^{2}=0.5$. The × in each graph corresponds to the mean value of *k*.

Figure 3 displays the histograms of the *omnibus* scalar *k* for each of the three normal distribution models. The use of $kL_{n}\left(\theta \right)$ will retrieve the sandwich variance $\Sigma_{n}^{\mathrm{sand}}$.

When $s^{2}=2$, the model underestimates the information contained in the data and the *omnibus* scalar is greater than one. Vice versa, for $s^{2}=0.5$ we systematically overestimate the informativeness of the data and, as a result, k<1, to slow down learning and produce robust confidence intervals for μ, the mean of the data-generating process. For $s^{2}=1$, the normal distribution model $N(m,s^{2})$ is equal to the standard normal distribution N(0,1) of the data generating process and k=1.

**Figure 4:**
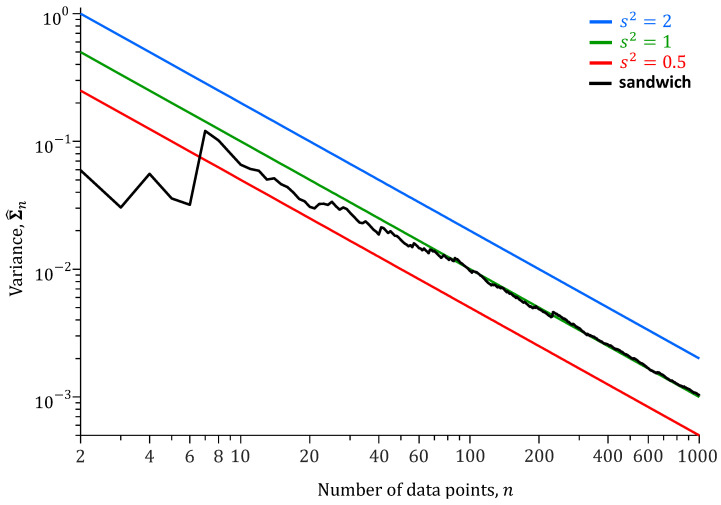
Relationship between number *n* of data points of the data generating process, Ω∼N(0,1) and the naive variance $\Sigma_{n}^{\mathrm{naive}}$ of the ML estimate $\hat{m}$ of the mean of the normal distribution model $N(m,s^{2})$ using $s^{2}=2$ (blue), $s^{2}=1$ (green) and $s^{2}=1/2$ (red). The black line displays the evolution of the sandwich variance estimator $\Sigma_{n}^{\mathrm{sand}}$. This estimator is invariant to the choice of $s^{2}$.

To better understand the relationship between the number *n* of data points ω and the naive and sandwich variances, we repeat the analysis of Table 1 for different values of *n*. Figure 4 presents the results of this analysis.

The naive variance decreases linearly on a logarithmic scale with the length *n* of the training record. The slope of this line is proportional to 1/n on a linear scale. The naive variance of *m* depends on the choice of $s^{2}$. The sandwich variance does not depend on $s^{2}$ and settles on the *true* variance of *m* with increasing number of data points ω.

Table 2 examines the coverage probabilities of the true mean μ of the data generating process according to the 100(1−α)% confidence intervals of $\hat{\mu }$ derived from the naive and sandwich variance estimators.

The results of Table 2 demonstrate that the sandwich variance estimator provides adequate confidence intervals of the mean μ of the data-generating process, even if the underlying model is misspecified. The sandwich estimator $\Sigma_{n}^{\mathrm{sand}}$ consistently achieves the correct frequentist coverage probabilities, whereas the naive variance estimator $\Sigma_{n}^{\mathrm{naive}}$ either over- ($s^{2}=2$) or under- ($s^{2}=1/2$) estimates the coverage probabilities. The confidence intervals are either too dispersed or too sharp.

This concludes our first case study. This study was rather unrealistic in that misspecification was introduced by affixing one of the model parameters $s^{2}$ to a wrong value. The correct distribution was used, but with a wrong value for one of its parameters, namely the variance $s^{2}$. In the next study, we are going to take misspecification one step further and use a different model for inference than was used to generate the data.

### 6.2. Case Study 2

Our second case study is another analytic exercise, but one that better reflects practice as the parametric form of our model differs from that of the data-generating process. We draw *n* measurements $\omega_{1},\ldots ,\omega_{n}$ from a gamma distribution Ω∼G(a,b) with pdf

(28) $$f_{G}\left(\omega |a,b\right)=\frac{1}{b^{a}\Gamma \left(a\right)}\omega^{a-1}\exp\left(-\omega /b\right),\;\omega \geq 0,$$

where a>0 and b>0 are shape and scale parameters, respectively, and Γ(z) is the Gamma function. Now, suppose our model for Ω is not a gamma but an exponential distribution E(μ) with location parameter μ>0 and pdf

$$f_{E}\left(\omega |\mu \right)=\mu^{-1}\exp\left(-\omega /\mu \right),\;\omega \geq 0.$$

Note that it is not uncommon to parameterize E(μ) with a rate parameter $\lambda =\mu^{-1}$ instead. The likelihood function for a single observation ω is now equal to

$$L_{\omega }\left(\mu \right)=f\left(\omega |\mu \right)=\mu^{-1}\exp\left(-\omega /\mu \right),$$

and the log-likelihood $L_{\omega }\left(\mu \right)$ becomes

$$L_{\omega }\left(\mu \right)=\log\left(L_{\omega }\left(\mu \right)\right)=-\log\left(\mu \right)-\omega /\mu .$$

Appendix D derives analytic expressions for the naive and sandwich variance estimators of the mean of the exponential distribution. We yield

(29) $$\Sigma_{n}=\left\{\begin{array}{cc} \Sigma_{n}^{\mathrm{naive}}=m_{\omega }^{2}/n={\hat{I}}_{n}^{-1}, & \mathrm{naive}\;\mathrm{variance},\\ \Sigma_{n}^{\mathrm{sand}}=s_{\omega }^{2}/n={\hat{G}}_{n}^{-1}, & \mathrm{sandwich}\;\mathrm{variance}. \end{array}\right.$$

where $m_{\omega }$ and $s_{\omega }^{2}$ are the sample mean and sample variance of the data points, $\omega_{1},\ldots ,\omega_{n}$. According to Equation (A12), the *omnibus* scalar *k* is now equal to $\hat{k}=m_{\omega }^{2}s_{\omega }^{-2}$, whereas its theoretical value, derived in Equation (A13), corresponds to the shape parameter *a* of G(a,b).

Table 3 confirms again the erroneous description of the confidence intervals by the naive variance estimator. The 100(1−α)% confidence intervals are too sharp and underestimate the theoretic coverage probabilities of the mean μ of the data generating process. This overconditioning is a result of misspecification and, thus, due to a misalignment of the sensitivity and variability matrices. In contrast to other methods, the coverage probabilities of the sandwich estimator align much more closely with theoretical expectations. The estimates are not perfect, as a result of the symmetry assumption used in constructing confidence intervals for $\hat{\mu }$. This assumption is not valid for the exponential distribution and is further exacerbated when the sample size *n* is small. To mitigate this latter effect, we chose n=100 in our Monte Carlo experiments. To address the asymmetry, one could construct non-symmetric confidence intervals by identifying the shortest interval for $\hat{\mu }$ that contains the true mean with probability 1−α. However, doing so would require knowledge of the posterior distribution of $\hat{\mu }$, which is generally not available in frequentist settings. Importantly, the variance of the ML estimates $\hat{\mu }$ across the *M* Monte Carlo trials matches the theoretical sandwich variance, $\Sigma_{n}^{\mathrm{sand}}=a^{2}b^{2}/n$. This confirms that the only correct confidence intervals of $\hat{\mu }$ are those derived from the sandwich estimator.

Table 4 documents the nominal coverage probabilities of the credible regions obtained from MCMC simulation with the DREAM_(ZS)_ algorithm using the log-likelihood $L_{n}\left(\mu \right)$ of Equation (A10) (=naive estimator), OFS-adjusted naive posterior samples of Equation (11), magnitude-adjusted log-likelihood $kL_{n}\left(\mu \right)$ with *omnibus* scalar *k* of Equation (10) (=Algorithm 1), curvature-adjusted log-likelihood $L_{n}^{\mathrm{ca}}\left(\mu \right)=L_{n}\left(\hat{\mu }+C_{n}\left(\mu -\hat{\mu }\right)\right)$ of Equation (15) (=Algorithm 2), and centralized power log-likelihood $L_{n}^{p}\left(\mu |\lambda \right)$ of Equation (21) (=Algorithm 3).

The curvature-adjustment matrix $C_{n}$ is a scalar in this case, and according to Equations (18) and (29), we yield $C_{n}=m_{\omega }s_{\omega }^{-1}$, where $s_{\omega }^{-1}$ is the reciprocal of the sample standard deviation of the ω’s. For OFS-adjustment, we substitute the expressions for $A_{*}$ and $B_{*}$ of Equation (A7) into Equation (12) and yield $\Psi_{n}=s_{\omega }/m_{\omega }$. The tabulated values confirm that

1. The asymptotic covariance matrix of the Metropolis algorithm is a single slice of bread. The 100(1−α)% credible intervals are in agreement with the frequentist confidence intervals of the naive variance estimator, $\Sigma_{n}^{\mathrm{naive}}$ in Table 3 and underestimate the theoretic coverage probabilities.
2. The OFS adjustment of Equation (11) enlarges the spread of the naive posterior samples but the coverage probabilities of the so-obtained sandwich credible regions underestimate their counterparts of the sandwich estimator in Table 3.
3. The three MCMC recipes discussed in this paper successfully join a single slice of bread $\frac{1}{n}A_{n}^{-1}$ to the open-faced sandwich $B_{n}A_{n}^{-1}$ to produce the sandwich variance $\Sigma_{n}^{\mathrm{sand}}$. The coverage probabilities of the 100(1−α)% credible regions of Algorithms 1–3 match those of the sandwich estimator in Table 3.
4. The tabulated values for Algorithm 3 are the first proof that the centralized power log-likelihood function $L_{n}^{p}\left(\mu |\lambda \right)$ of Equation (21) works in practice. This inspires confidence that we can sample the sandwich distribution without using matrix square roots.

The OFS adjustment is computationally appealing and enlarges the spread of the naive posterior samples, yet the so-obtained credible regions underestimate the theoretical coverage probabilities. Magnitude, curvature, and kernel adjustment of the log-likelihood function all appear viable methods for sandwich-adjusted MCMC simulation. There are important differences between these three sampling methods and the practical consequences of this are better illustrated with a multivariate target distribution.

**Figure 5:**
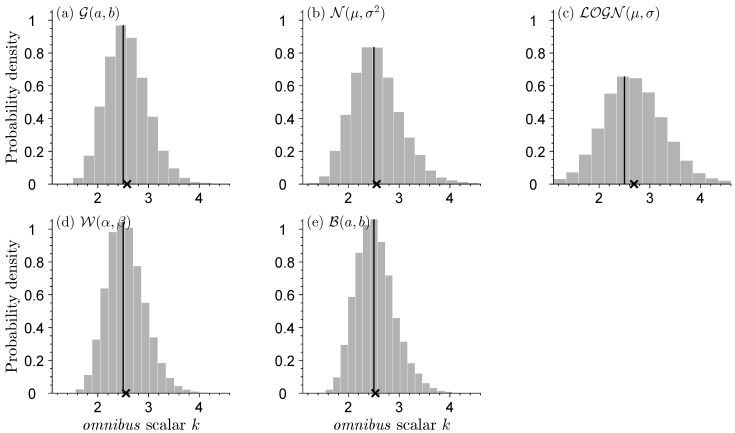
Histogram of the *omnibus* scalar *k* for $M=10^{4}$ Monte Carlo simulations using the (**a**) Gamma, (**b**) Normal, (**c**) Lognormal, (**d**) Weibull and (**e**) Beta distributions for the data generating process. The mean value of *k* is separately displayed in each graph with the solid cross, whereas the vertical black line is the theoretic value of the *omnibus* scalar.

Having completed the above exercise, we now replace G(a,b) with alternative distributions for the data-generating process. Figure 5 shows histograms of the *omnibus* scalar *k* when the data-generating process is (a) G(a,b), (b) $N(\mu ,\sigma^{2})$, (c) LOGN(μ,σ), (d) W(α,β), and (e) B(a,b). For comparability, the scale, shape, and/or location parameters of each distribution are chosen such that E[ω]=1/2 and Var[ω]=1/10. The theoretical value of the omnibus scalar for each distribution is $k=\mu^{2}\sigma^{-2}=2.5$.

The histograms of $\hat{k}$ appear remarkably similar across the different distributions. This confirms that our inferences for μ are robust and do not depend on the distribution of the data-generating process. The marginal distributions of the omnibus scalar center on the theoretic value of k=2.5 and display a small right tail. The dispersion of $\hat{k}$ is a result of sample size and will disappear if we set *n* much larger in the Monte Carlo trials.

We now move on to our third and last case study. This will involve the use of real-world data and a multivariate posterior distribution.

### 6.3. Case Study 3

Our third and final case study examines the streamflow response of the Leaf River near Collins, MS, USA. The precipitation–discharge transformation is simulated using the Xinanjiang conceptual watershed model originally developed by Zhao and Zhuang [65]. We adopt the implementation of Jayawardena and Zhou [75] and Knoben et al. [76], augmented with a pan evaporation parameter and three linear routing reservoirs. This configuration comprises seven control volumes that conceptually represent water storage and routing. Appendix E provides a detailed description of the Xinanjiang model structure, including the control volumes, state variables, flux relationships, and routing scheme used to convert areal average precipitation into total channel inflow and river discharge. The model equations are solved using a mass-conservative, second-order integration method with adaptive time stepping, ensuring both numerical stability and accuracy. A one-year spin-up period removes the influence of state variable initialization.

Table A2 lists the 14 parameters of the Xinanjiang model to be estimated from streamflow measurements. For inference, we express the Xinanjiang model as the vector-valued regression

ω=f(θ,I)+e,

where $\omega ={\left(\omega_{1},\ldots ,\omega_{n}\right)}^{⊤}$ is the n×1 vector of discharge observations, $\theta ={\left(f_{p},A_{\mathrm{im}},a,b,f_{\mathrm{wm}},f_{\mathrm{lm}},c,s_{\mathrm{tot}},\beta ,k_{i},k_{g},c_{i},c_{g},k_{f}\right)}^{⊤}$ signifies the parameter vector, I is the n×2 matrix of exogenous variables containing daily areal-average rainfall and potential evapotranspiration, and $e={\left(e_{1},\ldots ,e_{n}\right)}^{⊤}$ is the n×1 vector of discharge measurement errors. We assign a uniform prior P(θ) over the bounds given in Table A2 and use the standardized skewed-*t* (SST) density of Scharnagl et al. [77] to evaluate agreement between observed and simulated streamflows

(30) $$\begin{array}{cc} f_{\mathrm{SST}}\left(\epsilon_{t}|0,1,\nu ,\xi \right) & =\frac{2\sigma_{\nu \xi }}{\left(\xi +\xi^{-1}\right)}\frac{\Gamma \left((\nu +1)/2\right)}{\Gamma \left(\nu /2\right)\sqrt{\pi (\nu -2)}}{\left[1+\frac{1}{\nu -2}{\left(\frac{\mu_{\nu \xi }+\sigma_{\nu \xi }\epsilon_{t}}{\xi^{\mathrm{sign}(\mu_{\nu \xi }+\sigma_{\nu \xi }\epsilon_{t})}}\right)}^{\;\;2}\right]}^{-(\nu +1)/2}, \end{array}$$

where $\epsilon_{t}=e_{t}/s_{t}$ is the *t*^th^ studentized streamflow residual, sign(x)=|x|/x, denotes the signum function, and scalars $\mu_{\nu \xi }=M_{1}\left(\xi -\xi^{-1}\right)$ and $\sigma_{\nu \xi }={\left\{\left(M_{2}-M_{1}^{2}\right)\left(\xi^{2}+\xi^{-2}\right)+2M_{1}^{2}-M_{2}\right\}}^{1/2}$ are shift and scale constants, respectively, which depend on the degrees of freedom ν>2, the skewness parameter ξ>0, and the first and second absolute moments $M_{1}$ and $M_{2}$ of the SST density [57,77]. The total likelihood $L_{n}\left(\theta ,\nu ,\xi \right)$ for a *n*-record of studentized residuals $\epsilon_{1}\left(\theta \right),\ldots ,\epsilon_{n}\left(\theta \right)$ is now equal to

$$\begin{array}{cc} L_{n}\left(\theta ,\nu ,\xi \right) & =C\left(\nu ,\xi ,n\right)\prod_{t=1}^{n}{\left[1+\frac{1}{\nu -2}{\left(\frac{\mu_{\nu \xi }+\sigma_{\nu \xi }\;\epsilon_{t}\left(\theta \right)}{\xi^{\mathrm{sign}(\mu_{\nu \xi }+\sigma_{\nu \xi }\;\epsilon_{t}\left(\theta \right))}}\right)}^{\;2}\;\right]}^{\;-(\nu +1)/2}, \end{array}$$

where the prefactor C(ν,ξ,n) is

$$\begin{array}{cc} C(\nu ,\xi ,n) & ={\left(\frac{2\sigma_{\nu \xi }}{\left(\xi +\xi^{-1}\right)}\frac{\Gamma \left((\nu +1)/2\right)}{\Gamma \left(\nu /2\right)\sqrt{\pi (\nu -2)}}\right)}^{\;n}. \end{array}$$

The measurement error standard deviation $s_{t}$ of the *t*^th^ streamflow observation $\omega_{t}$ is modeled as a linear function of the simulated discharge $y_{t}\left(\theta \right)$ under model parameters θ

$$\begin{array}{cc} s_{t} & =s_{0}+s_{1}y_{t}\left(\theta \right), \end{array}$$

where the intercept $s_{0}=10^{-4}$ (mm/d) is fixed at a small positive value, and the slope $s_{1}>0$ is determined offline so as to enforce unit variance of the studentized raw residuals $\epsilon_{1}\left(\theta \right),\ldots ,\epsilon_{n}\left(\theta \right)$. The slope is obtained via an iterative root-finding procedure described in detail by Vrugt et al. [57]. With this variance model, the Student–*t* log-likelihood becomes

(31) $$\begin{array}{cc} L_{n}^{s}\left(\theta ,\nu ,\xi |s_{0}\right) & ≃n\log\left(C(\nu ,\xi ,1)\right)-\sum_{t=1}^{n}\left\{\log(\right|s_{0}+s_{1}y_{t}\left(\theta \right)|)\}-\frac{\nu +1}{2}\sum_{t=1}^{n}\left\{\log\left[1+\frac{1}{\nu -2}{\left(\frac{\mu_{\nu \xi }+\sigma_{\nu \xi }\;\epsilon_{t}\left(\theta \right)}{\xi^{\mathrm{sign}(\mu_{\nu \xi }+\sigma_{\nu \xi }\;\epsilon_{t}\left(\theta \right))}}\right)}^{\;2}\;\right]\right\}. \end{array}$$

To facilitate both pairwise and parameter-wise comparisons of the d×d sensitivity $A_{n}$ and variability $B_{n}$ matrices, we apply the affine rescaling

$$\begin{array}{cc} {\underline{\theta }}_{j} & =\frac{\theta_{j}-\theta_{j}^{\min}}{\theta_{j}^{\max}-\theta_{j}^{\min}}\;\mathrm{for}\;j=1,\ldots ,14,\;\mathrm{and}\;\;{\underline{\eta }}_{r}=\frac{\eta_{r}-\eta_{r}^{\min}}{\eta_{r}^{\max}-\eta_{r}^{\min}}\;\mathrm{for}\;r=1,2, \end{array}$$

which maps the Xinanjiang parameters $\theta ={\left(\theta_{1},\ldots ,\theta_{14}\right)}^{⊤}$ and nuisance variables $\eta ={\left(\nu ,\xi \right)}^{⊤}$ onto the unit hypercube. Inference is then conducted on the normalized parameters

$$\begin{array}{cc} \underline{\theta } & ={\left({\underline{f}}_{p},{\underline{A}}_{\mathrm{im}},\underline{a},\underline{b},{\underline{f}}_{\mathrm{wm}},{\underline{f}}_{\mathrm{lm}},\underline{c},{\underline{s}}_{\mathrm{tot}},\underline{\beta },{\underline{k}}_{i},{\underline{k}}_{g},{\underline{c}}_{i},{\underline{c}}_{g},{\underline{k}}_{f}\right)}^{⊤}, \end{array}$$

and normalized nuisance variables $\underline{\eta }={\left(\underline{\nu },\underline{\xi }\right)}^{⊤}$. Prior to Xinanjiang model execution, $\underline{\theta }$ is transformed back to the original parameter scales using the lower and upper bounds in Table A2. The prior distributions for the degrees of freedom and skewness parameters are uniform with support $\nu \in (2,10^{4}]$ and $\xi \in [10^{-1},10^{2}]$, respectively.

**Figure 6:**
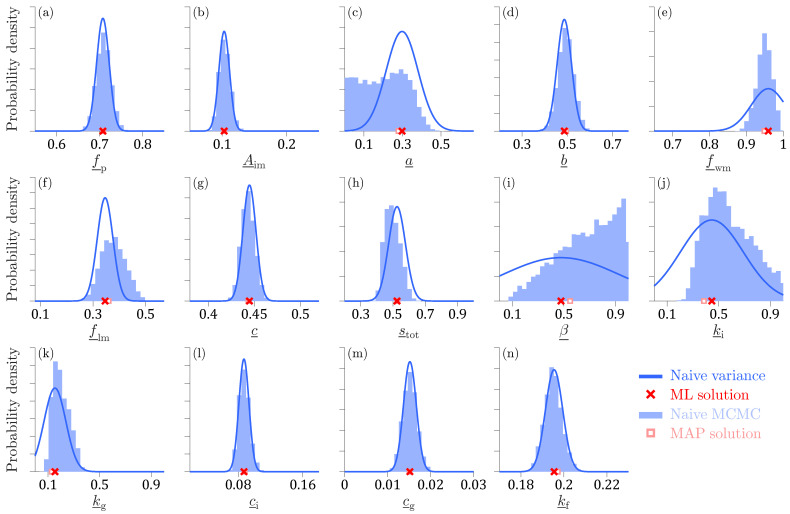
Marginal posterior distributions (blue histograms) of the Xinanjiang parameters (**a**) $f_{p}$, (**b**) $A_{\mathrm{im}}$, (**c**) *a*, (**d**) *b*, (**e**) $f_{\mathrm{wm}}$, (**f**) $f_{\mathrm{lm}}$, (**g**) *c*, (**h**) $s_{\mathrm{tot}}$, (**i**) β, (**j**) $k_{i}$, (**k**) $k_{g}$, (**l**) $c_{i}$, (**m**) $c_{g}$, and (**n**) $k_{f}$ obtained from the DREAM_(ZS)_ algorithm. Inference is based on the Student *t* likelihood function $L_{n}^{s}\left(\theta ,\nu ,\xi |s_{0}=10^{-4}\right)$ and a uniform prior distribution. The solid blue lines display the normal marginal distributions derived from the naive frequentist variance estimator. The red × corresponds to the ML estimator, whereas the red square is the MAP solution of the sampled Markov chains. To conserve space, we do not display numerical labels on the *y*-axis.

Figure 6 shows histograms of the marginal posterior distributions of the normalized Xinanjiang model parameters obtained using the DREAM_(ZS)_ algorithm.

The Markov chain sample with highest value of $P_{n}\left(\underline{\theta },\underline{\nu },\underline{\xi }\right)=P\left(\underline{\theta },\underline{\nu },\underline{\xi }\right)L_{n}^{s}\left(\underline{\theta },\underline{\nu },\underline{\xi }|s_{0}=10^{-4}\right)$ (red square) coincides almost perfectly with the ML solution (red cross) of the frequentist estimator separately obtained by maximizing the Student *t* likelihood $L_{n}^{s}\left(\underline{\theta },\underline{\nu },\underline{\xi }|s_{0}=10^{-4}\right)$ using a gradient-based optimization method. For all Xinanjiang model parameters except the tension water inflection parameter *a* and the free water shape parameter β, the MCMC-sampled marginal posterior distributions are unimodal, bell-shaped and centered around the ML solution. In contrast, the marginal posterior distribution of *a* is approximately uniform on the interval between 0–0.4, whereas the density function of β has a trapezoidal shape. The MAP values of these two parameters do not coincide with distinct posterior peaks, yet are in close vicinity of their ML estimates.

Most of the MCMC-sampled posterior histograms are in close agreement with the normal marginal distributions (blue lines) derived from the naive variance $\Sigma_{n}^{\mathrm{naive}}=\frac{1}{n}A_{n}^{-1}$ of the frequentist estimator where the sensitivity matrix $A_{n}=-\frac{1}{n}\nabla^{2}L_{n}^{s}\left({\hat{\underline{\theta }}}_{*},{\hat{\underline{\nu }}}_{*},{\hat{\underline{\xi }}}_{*}|s_{0}=10^{-4}\right)$ is computed from the second-order partial derivatives of the Student *t* likelihood function. This frequentist estimator assumes model linearity and a symmetric Gaussian distribution around the ML estimate. In contrast, the MCMC method approximates the marginal distributions of the parameters from a large sample of posterior realizations. This enables MCMC to account for nonlinear model relationships and represent arbitrary posterior shapes, including skewed and heavy-tailed distributions. As a result, frequentist and Bayesian estimates of parameter uncertainty may differ. In the literature, this distinction is often framed in terms of linear versus nonlinear confidence intervals. However, in the Bayesian context, the more appropriate term is credible intervals, which reflect the probabilistic interpretation of uncertainty inherent to Bayesian inference.

Table A3 in Appendix F shows that most Xinanjiang parameters exhibit only weak correlations. Notable exceptions are the recession parameters $k_{i}$ and $k_{g}$ of the interflow and groundwater reservoirs, respectively, which display a very strong correlation (r=0.985), followed by a correlation of r=0.856 between the tension water inflection parameter *a* and the total soil moisture storage $s_{\mathrm{tot}}$, and r=0.782 between the fraction of tension water storage $f_{\mathrm{wm}}$ and the free water distribution shape parameter β. The generally low posterior correlation coefficients account in part for the close agreement between the naive posterior histograms of the Xinanjiang parameters and the normal marginal distributions derived from the frequentist estimator.

Before examining the posterior Xinanjiang parameter distributions obtained from Algorithms 1–3, we first take a closer look in Table 5 at the bread and meat matrices of the Student *t* likelihood $L_{n}^{s}\left(\theta ,\nu ,\xi |s_{0}=10^{-4}\right)$. Comparing these two matrices offers insight into the magnitude of the sandwich correction.

The main diagonal entries of $A_{n}^{s}$ and $\beta n_{s}$ are in relatively poor agreement. The bolded entries of $\beta n_{s}$ are nearly an order of magnitude larger than their counterparts of $A_{n}^{s}$. This gives rise to a value of k=0.1327 for the *omnibus* scalar of Pauli et al. [31] in Equation (10). This value is far removed from the desired value of k=1 under correct specification. In Section 9, we formulate several other quantitative measures of (dis)similarity between the bread and meat matrices. This includes the Frobenius norm of the naive and sandwich variance matrices in Equation (35). The norm exceeds 2.0, indicating substantial misspecification and underscoring the need for the sandwich estimator to robustly quantify Xinanjiang parameter uncertainty.

In Table A4 of Appendix G we compare the frequentist bread matrix $A_{n}^{s}$ with the inverse of the covariance matrix of the DREAM_(ZS)_-sampled naive posterior realizations. The MCMC-derived bread matrix is in reasonable agreement with $A_{n}^{s}$, consistent with the close correspondence observed in Figure 6 between the frequentist characterization of naive parameter uncertainty and the normal posterior histograms sampled by the DREAM_(ZS)_ algorithm for most parameters. The marginal posterior distributions of the tension water inflection parameter *a* and the free water shape parameter β deviate noticeably from normality, which explains in part the relatively large differences in their diagonal elements of the bread matrices of the frequentist and MCMC methods. The largest discrepancy is observed for the parameter $f_{\mathrm{wm}}$, whose MCMC-derived bread matrix value on the main diagonal is 0.122, approximately 40 times smaller than the corresponding value of 4.860 from the frequentist estimator. The culprit may be the prior distribution, which truncates the posterior distribution of $f_{\mathrm{wm}}$ at unity but does not affect the normal approximation underlying the frequentist characterization of naive parameter uncertainty. Thus, in summary, good agreement between the linear (frequentist-based) and nonlinear (Bayesian sample-based) estimates of the sensitivity (bread) matrix suggests that the posterior distribution is approximately Gaussian, and that the multinormal frequentist description of the ML uncertainty is consistent with the fully Bayesian approach.

**Figure 7:**
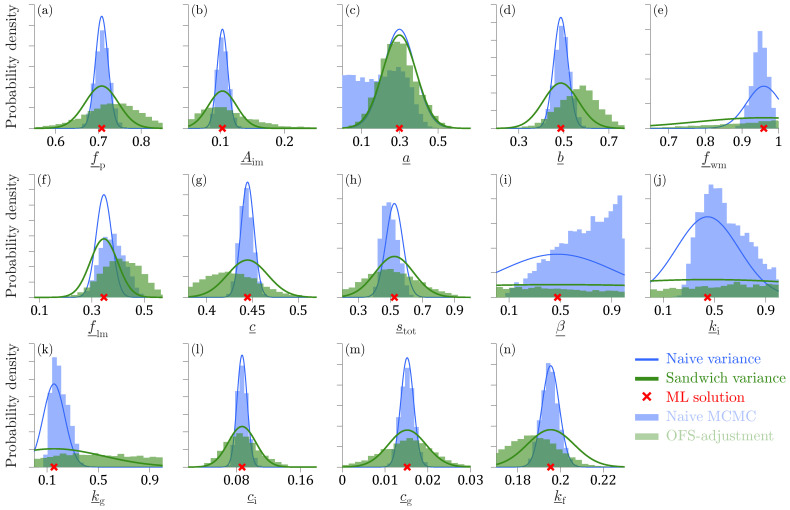
Marginal distributions of the OFS-adjusted naive posterior samples of the Xinanjiang parameters (**a**) $f_{p}$, (**b**) $A_{\mathrm{im}}$, (**c**) *a*, (**d**) *b*, (**e**) $f_{\mathrm{wm}}$, (**f**) $f_{\mathrm{lm}}$, (**g**) *c*, (**h**) $s_{\mathrm{tot}}$, (**i**) β, (**j**) $k_{i}$, (**k**) $k_{g}$, (**l**) $c_{i}$, (**m**) $c_{g}$ and (**n**) $k_{f}$ obtained from Equation (11). The solid blue and green lines display the normal frequentist distributions of the naive and sandwich variance estimators. The blue histograms correspond to the naive posterior parameter distributions of Figure 6 and the red × highlights the ML solution.

Figure 7 presents histograms of the OFS-adjusted posterior samples of the Xinanjiang parameters and degrees of freedom ν of the Student *t* likelihood function $L_{n}^{s}\left(\theta ,\nu ,\xi |s_{0}=10^{-4}\right)$. The OFS-adjusted posterior samples are derived from Equation (11) using $\Psi_{n}=A_{n}^{-1}B_{n}^{1/2}A_{n}^{1/2}$ where the matrix square roots, $A_{n}^{1/2}$ and $B_{n}^{1/2}$, are computed according to Equation (13) using singular value decomposition.

The OFS-adjustment enhances substantially the dispersion of the posterior samples for all Xinanjiang parameters but the tension water inflection parameter *a*. The histograms of the OFS-adjusted posterior samples (green bars) stretch far beyond the normal marginal distributions (blue lines) derived from the sensitivity matrix $A_{n}$ of second-order partial derivatives of the Student *t* log-likelihood function w.r.t. the ML solution {θ,ν,ξ} and the naive (blue) histograms of the Xinanjiang parameters. The culprit is model misspecification and, consequently, a poor alignment of the sensitivity $A_{n}$ and variability $\beta_{n}$ matrices. The OFS-derived sandwich histograms of the Xinanjiang parameters are in reasonable agreement with the normal marginal distributions (green lines) of the frequentist estimator of $\Sigma_{n}^{\mathrm{sand}}$. Note that the OFS-adjusted sandwich density functions for $f_{\mathrm{wm}}$, β, and $k_{i}$ are visibly lower than their corresponding frequentist densities. This discrepancy arises because the OFS transformation in Equation (11) does not honor the unit interval of the normalized Xinanjiang parameters. Infeasible parameter values lower the probability density of the adjusted posterior samples within the admissible range. Last but not least, for several parameters, the OFS adjustment of Equation (11) altered the location of the mode (peak) of the sandwich distribution. The most notable shifts occurred for $f_{p}$, *b*, $f_{\mathrm{lm}}$, *c*, $k_{g}$, and $k_{f}$. Such changes are somewhat counterintuitive and arise in part from the non-uniqueness of the matrix square roots $A_{n}^{1/2}$ and $B_{n}^{1/2}$ used in the adjustment.

**Figure 8:**
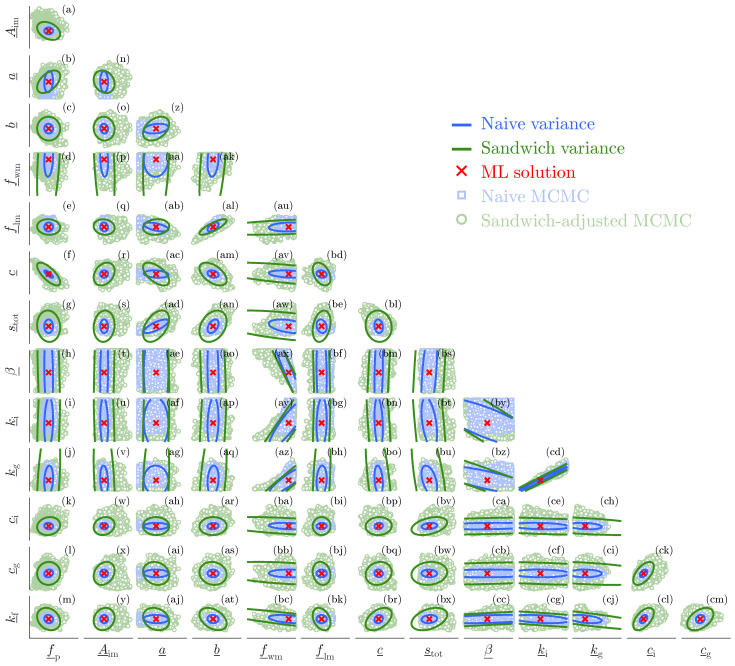
Scatter plot matrix of bivariate confidence and credible regions for all pairs of Xinanjiang model parameters. The ellipsoids show frequentist 95% confidence intervals estimated from the naive variance $\Sigma_{n}^{\mathrm{naive}}=\frac{1}{n}A_{n}$ (in blue) and the sandwich variance Σnsand=1nAn−1Bn−1An−1 (in green). The blue squares and green dots represent the 95% credible regions of the naive and sandwich-adjusted posterior distributions sampled by the DREAM_(ZS)_ algorithm. Axis values are omitted to save space.

Figure 8 presents a matrix plot of the bivariate 95% confidence (lines) and credible (dots) regions of all pairs of Xinanjiang parameters. The blue area corresponds to the naive variance whereas the green area is associated with the sandwich-adjusted posterior samples of Algorithm 3.

The bivariate scatter plots offer a clearer depiction of the naive and sandwich uncertainty estimates for the Xinanjiang parameters. The following conclusions can be drawn.

1. The naive Bayesian 95% credible regions (blue squares), as sampled by the DREAM_(ZS)_ algorithm are in strong agreement with the frequentist 95% confidence ellipsoids derived from the naive variance estimator. There are some notable exceptions, particularly in the bivariate scatter plots involving parameter *a*, where the MCMC-sampled naive confidence regions exceed the frequentist ellipsoids. This is a well-known phenomenon that highlights the distinction between linear and nonlinear confidence (or credible) regions [49,78,79,80].
2. The 95% credible regions of the sandwich-adjusted posterior samples (green dots) extend well beyond the sandwich ellipsoids (green lines) of the frequentist estimator. These linear sandwich confidence regions substantially underestimate the true parameter uncertainty, and appear woefully inadequate for accurately characterizing Xinanjiang discharge uncertainty.
3. For most parameter pairs, the MCMC-derived sandwich credible regions are unimodal and well described by a bivariate normal distribution.
4. The sandwich credible regions of the Xinanjiang parameters are much larger than their naive counterparts. This is a result of misspecification and confirms that the sensitivity (bread) matrix $A_{n}^{s}$ of the Student *t* likelihood function substantially overestimates the information content of the discharge observations. The only valid currency of discharge data informativeness under model misspecification is the Godambe information, as expressed by the sandwich credible regions. The enlarged parameter uncertainty should yield the appropriate parameter coverage probabilities.

Substantial differences between linear and nonlinear confidence regions, such as those observed for the tension water inflection parameter *a*, often signal problems in model formulation. Other indicators of model misspecification include parameters whose MAP estimates occur at or near the bounds of their prior ranges. Although the Xinanjiang model does not exhibit this behavior for the Leaf River dataset, practical experience with other conceptual hydrologic models suggests that such issues are more common than rare. When a MAP estimate lies close to a parameter bound, the local curvature of the log-likelihood becomes poorly defined, making it difficult or impossible to compute a stable Hessian (bread) matrix. This, in turn, undermines the validity of asymptotic approximations in frequentist inference, such as the ML sandwich estimator used herein.

**Figure 9:**
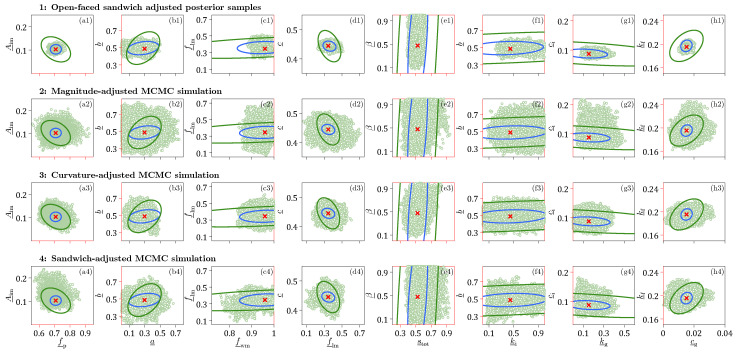
Comparison of 95% parameter credible regions derived from (1) OFS-adjusted sandwich samples and (2) magnitude-, (3) curvature- and (4) sandwich-adjusted MCMC simulation using Algorithms 1–3, respectively: (**a1**–**a4**) $f_{p}-A_{\mathrm{im}}$, (**b1**–**b4**) a−b, (**c1**–**c4**) $f_{\mathrm{wm}}-f_{\mathrm{lm}}$, (**d1**–**d4**) $f_{\mathrm{lm}}-c$, (**e1**–**e4**) $s_{\mathrm{tot}}-\beta$ (**f1**–**f4**) $k_{i}-b$, (**g1**–**g4**) $k_{g}-c_{i}$, and (**h1**–**h4**) $c_{g}-k_{f}$. The OFS-adjusted posterior samples are obtained from Equation (11) using Ψn=An−11/2Bn1/2An1/2 with matrix square roots $A_{n}^{1/2}$ and $B_{n}^{1/2}$ computed according to Equation (13) using singular value decomposition. The blue and green ellipsoids are the 95% confidence regions of the frequentist naive and sandwich variance estimators. Red lines delineate the boundaries of the standard uniform prior distribution.

Before turning our attention to Xinanjiang discharge uncertainty, we first examine in Figure 9 bivariate scatter plots of the OFS-adjusted posterior samples and their counterparts obtained from magnitude-, curvature-, and sandwich-adjusted MCMC simulation. For a direct comparison of the different methods, the same *x*- and *y*-axis limits are used for all four graphs in each column. We focus our attention on only a subset of the Xinanjiang parameter pairs.

The results in Figure 9 highlight several interesting observations.

1. The sandwich credible regions for the Xinanjiang parameters vary substantially across different sandwich-adjustment methods and often diverge from the ellipsoidal confidence regions obtained using the frequentist sandwich estimator.
2. The OFS-adjusted posterior samples in the top panel yield, on average, the smallest 95% credible regions for the Xinanjiang parameters. These regions are straightforward to construct from the naive posterior samples but systematically underestimate the width of the frequentist sandwich confidence regions (green lines). Moreover, the OFS transformation of Equation (11) does not guarantee preservation of the posterior mode. This is evident in Figure 7, where the peak of the OFS-adjusted posterior distributions has shifted away from the ML/MAP solution.
3. Magnitude-adjusted MCMC simulation yields, on average, the largest credible regions for the Xinanjiang parameters. The sandwich credible regions of this method usually extend beyond the frequentist sandwich ellipsoids, although not necessarily in both directions of parameter space. The magnitude-adjusted sandwich uncertainty is particularly large for the parameter pairs a−b and $k_{i}-b$, as shown in Figure 9b2 and Figure 9f2, respectively, with credible regions that extend almost the entire parameter space and appear truncated by the boundaries of the uniform prior distribution. This behavior may be an artifact of the *omnibus* scalar *k*, which does not preserve the directional asymmetries inherent in the bread and meat matrices. This requires the use of a separate scaling factor for each Xinanjiang parameter.
4. The 95% credible regions derived from curvature-adjusted MCMC simulation are the overall closest match to the 95% sandwich ellipsoids obtained from the frequentist estimator. The prime examples are the credible regions of $f_{p}-A_{\mathrm{im}}$ (Figure 9a3), a−b (Figure 9b3), $f_{\mathrm{lm}}-c$ (Figure 9d3), and $c_{g}-k_{f}$ (Figure 9h3). The sandwich credible regions center on the ML estimator, have a single peak, and appear well described by a multinormal distribution. Large discrepancies between the frequentist confidence regions and curvature-adjusted credible regions are visible for $f_{\mathrm{wm}}-f_{\mathrm{lm}}$ and $k_{g}-c_{i}$ in Figure 9c3 and Figure 9g3, respectively.
5. The sandwich-adjusted credible regions obtained from our SAMH algorithm closely align with those derived from curvature-adjusted MCMC simulation, though with a slightly enlarged dispersion. The sandwich credible regions are a compromise between the results of magnitude- and curvature-adjusted MCMC simulation. This result inspires confidence in the centralized power likelihood $L_{n}^{p}\left(\theta |\lambda \right)$ of Equation (23) and couples with the dynamic learning rate λ(θ) of Equation (25) to successfully infer the sandwich posterior distribution. This method avoids the need for principal matrix square roots $A_{n}^{1/2}$ and $B_{n}^{1/2}$ in constructing a Bayesian approximation to the frequentist sandwich distribution. The dynamic learning rate λ(θ) redistributes the posterior probability mass away from the ML solution according to the more robust sandwich description of the parameters of the Xinanjiang model.

We cannot prove that the sandwich credible regions are more accurate, as the pseudo-true values of the Xinanjiang model parameters that generated the observed discharge record are unknown. Instead, we rely on statistical theory, which establishes the sandwich estimator as the only valid asymptotic descriptor of data informativeness, and, consequently, parameter uncertainty under model misspecification.

**Figure 10:**
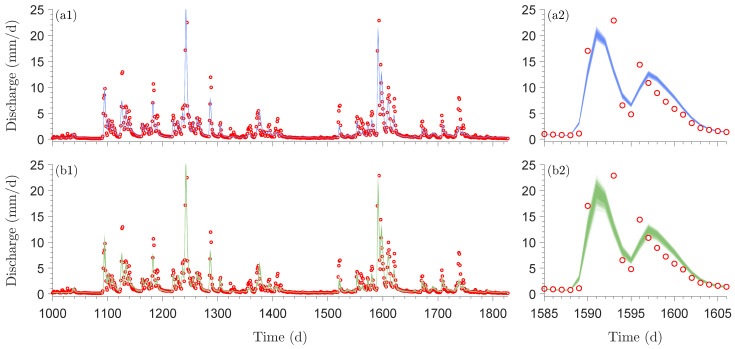
Simulation intervals for Xinanjiang streamflow based on the (**a**) naive and (**b**) sandwich variance estimators. Bands show the 68%, 90%, 95%, and 99% parameter uncertainty induced discharge intervals. Red dots are the observed discharge.

Figure 10 shows posterior predictive bands for simulated streamflow from Xinanjiang over a representative segment of the six-year training period, obtained by propagating posterior draws of θ through the model.

**Figure A2:**
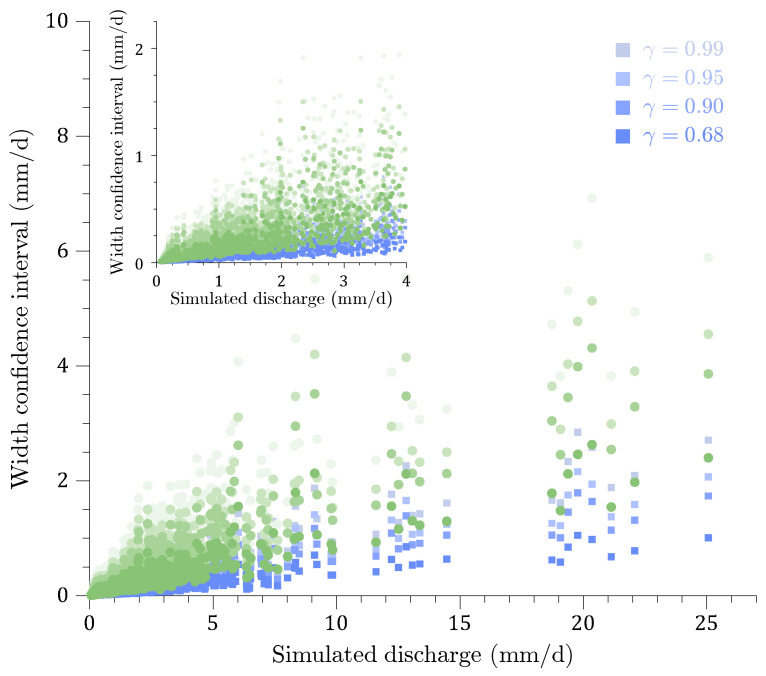
Width of the 100γ% streamflow credible intervals in units of mm/d resulting from naive (blue squares) and sandwich (green dots) parameter uncertainty, plotted as a function of the ML-simulated discharge values.

The sandwich variance estimator substantially widens the parameter uncertainty induced intervals for simulated streamflow from the Xinanjiang model, as evident in the right-hand panels. The 99% intervals expand markedly, especially near peak flows. Quantitatively, the 99%, 95%, 90% and 68% streamflow intervals based on sandwich parameter uncertainty contain 37.0%, 26.3%, 21.1% and 12.9% of the discharge observations, respectively, compared with 13.3%, 10.1%, 8.7% and 5.4% under the naive variance estimator. Thus, the naive 99% intervals achieve roughly the same coverage (≈13%) as the sandwich 68% intervals. In terms of width, the sandwich intervals are about twice as wide at low flows and roughly three times as wide at the hydrograph peaks (see Figure A2 in Appendix H).

**Figure 11:**
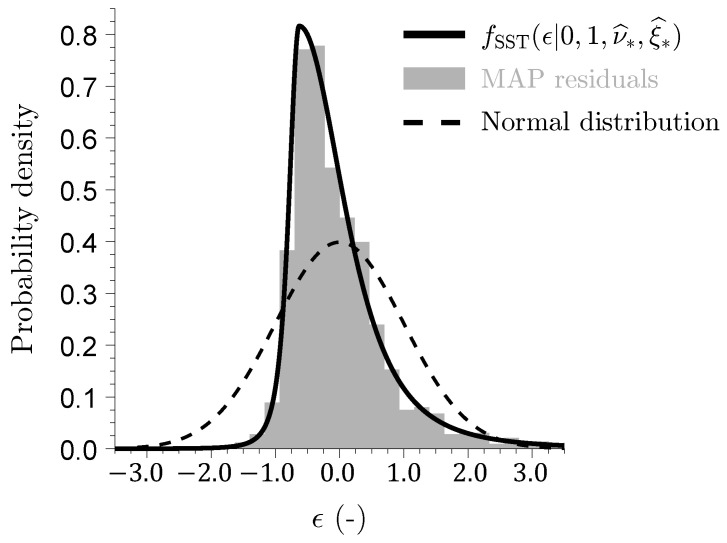
Histogram (gray bins) of the studentized residuals $\epsilon_{1}\left(\hat{\theta }\right),\ldots ,\epsilon_{n}\left(\hat{\theta }\right)$ of the Xinanjiang discharge simulation and SST density $f_{\mathrm{SST}}\left(\epsilon |0,1,\nu ,\xi \right)$ using ML values of the model parameters, degrees of freedom ν and kurtosis ξ. The probability density of the standard normal distribution $f_{N}\left(\epsilon |\mu =0,\sigma^{2}=1\right)$ is separately displayed with a dashed black line.

Finally, we examine the discharge residuals obtained from the ML parameter values of the Xinanjiang model. Figure 11 shows a histogram of the studentized discharge residuals $\epsilon_{1}\left({\hat{\theta }}_{*}\right),\ldots ,\epsilon_{n}\left({\hat{\theta }}_{*}\right)$, with gray bars normalized to represent a probability density estimate such that the total area under the bars amounts to one. We also plot the SST density $f_{\mathrm{SST}}\left(\epsilon |0,1,\nu ,\xi \right)$ of Equation (30) using the ML values of ν and ξ.

The histogram of the discharge residuals is in excellent agreement with the SST density. The studentized residuals $\epsilon_{1}\left(\hat{\theta }\right),\ldots ,\epsilon_{n}\left(\hat{\theta }\right)$ follow a Student *t* distribution with $\hat{\nu }=2.92$ degrees of freedom and skewness $\hat{\xi }=2.09$. This degrees of freedom is much smaller than one would expect from the sample size n=1827 and the number of parameters p=16 alone. This result once again confirms that the discharge residuals follow a Laplacian or double exponential distribution [11,57]. A skewness of $\hat{\xi }=2.09$ indicates that the distribution of MAP discharge residuals is right-skewed. Consequently, the mode (peak) of the distribution of the studentized streamflow residuals is located at −0.64, to the left of the median value of −0.20, which itself is smaller than the mean studentized residual of approximately 0.054. This mean value points to a negative bias in the Xinanjiang model, indicating a tendency, on average, to underestimate measured streamflows. The magnitude of this bias is around −0.14 mm/d or 11.3% of the mean measured discharge of 1.25 mm/d.

The SST density with low degrees of freedom exhibits both a sharper peak near its mean and heavier tails compared to the normal distribution (dotted line). This makes the Student *t* likelihood more robust to outliers and is well suited for inverse modeling of discharge data with the occasional large streamflow residuals. The largest residuals are typically attributable to precipitation measurement errors and are less governed by structural limitations and/or deficiencies of the hydrologic model. However, the sandwich estimator cannot distinguish between these two error sources. Both count as misspecification.

## 7. Numerical Estimation of the Sensitivity (Bread) Matrix

The naive and sandwich variance estimators rely on knowledge of the sensitivity matrix $A_{n}$ and variability matrix $B_{n}$ both evaluated at the ML estimator ${\hat{\theta }}_{*}$. Matrix $B_{n}=\frac{1}{n}\sum_{i=1}^{n}\nabla L_{\omega_{i}}\left({\hat{\theta }}_{*}\right)\nabla^{⊤}L_{\omega_{i}}\left({\hat{\theta }}_{*}\right)$ is constructed solely from first-order derivatives of the log-likelihood. When computed carefully either analytically or through numerical differentiation the resulting matrix is typically symmetric and positive definite. This is not true for the sensitivity matrix $A_{n}=-\frac{1}{n}\nabla^{2}L_{n}\left({\hat{\theta }}_{*}\right)$. The main challenge arises from the second-order derivatives of the log-likelihood, which are more difficult to compute than their first-order counterparts, especially when one or more parameters lie near their lower or upper bounds. The sensitivity matrix $A_{n}$ must be positive definite and therefore invertible to compute both the naive and sandwich variance estimators. If $A_{n}$ is not invertible, most textbooks advise that the model should be reconsidered, re-specified, and the analysis rerun, or, in some cases, that additional data should be collected. Holding certain model parameters constant at known or hypothesized values can restore invertibility, but this comes at the cost of a reduced model flexibility and potentially introducing bias if the fixed values are incorrect. Furthermore, model simplification affects the estimates of the remaining variables and therefore the interpretation of the findings [81].

Gill and King [41] suggest using a pseudo-factorization $A_{n}=V^{⊤}V$ of the sensitivity matrix if $A_{n}$ is not positive definite. Their so-called generalized Cholesky decomposition $V=\mathrm{gchol}(A_{n})$ avoids the failures of earlier factorization methods by Gill and Murray [40] and Gill et al. [82] by selectively modifying small or negative pivots. This yields a controlled decomposition even when $A_{n}$ is indefinite or nearly singular. The resulting pseudo-variance matrix ${\left(V^{⊤}V\right)}^{-1}=V^{-1}{\left(V^{⊤}\right)}^{-1}$ serves as a stand-in for $A_{n}^{-1}$. While this provides a computational workaround, it does not resolve the underlying invertibility problem; it merely allows variance estimation to proceed despite numerical artifacts. Consequently, when the sensitivity matrix is not invertible, results should be interpreted with caution, and model diagnostics and a careful reevaluation of assumptions remain essential.

Another possibility that requires almost no additional computation is to derive matrix $A_{n}$ from samples of the naive posterior distribution [25]. Theory establishes that this distribution will be asymptotically normal around the MAP estimator ${\hat{\theta }}_{*}$ and with a covariance matrix equal to a single slice of bread $\frac{1}{n}A_{n}^{-1}$. Thus, we can use the post-burn-in naive posterior samples as estimators of the bread matrix, ${\hat{A}}_{n}=\frac{1}{n}\mathrm{Cov}{\left[\left\{\theta_{\left(b\right)},\theta_{\left(b+1\right)},\ldots ,\theta_{\left(T\right)}\right\}\right]}^{-1}$. Alternatively, we retain the results of the evaluations of $L_{n}\left(\theta \right)$ at each iteration of the sampler and use them to numerically estimate the Hessian matrix at ${\hat{\theta }}_{*}$. This Hessian approximation will generally be a good estimator of $A_{n}$.

## 8. Limitations of Sandwich-Adjusted MCMC Simulation

Unlike existing approaches that rely on arbitrary matrix square roots, eigendecompositions or a single scaling factor applied uniformly across the parameter space, our method employs a parameter-dependent learning rate λ(θ) that enables direction-specific tempering of the likelihood. This allows the sampler to capture directional asymmetries in the sandwich distribution, particularly under model misspecification or in small-sample regimes, and yields credible regions that remain valid when standard Bayesian inference underestimates uncertainty. In our research for this paper, we identified one potential weakness of our methodology. When the posterior distribution is multimodal and these modes are disconnected, then the learning rate λ(θ) can suppress one of the peaks, thereby inflating the probability mass of one or more other peaks. The sandwich-adjusted chains then concentrate on the other modes. Through our investigations, we found that a simple and effective remedy is to restrict the learning rate to the interval (0,1]. This preserves the multimodal structure of the posterior sandwich distribution.

## 9. Formal Measures for the Degree of Model Misspecification

The misalignment of the naive and sandwich variance estimators can be summarized by scalar measures of model misspecification. This idea is not new. For example, White [83] developed an information matrix test to assess whether the discrepancy between $A_{n}$ and $B_{n}$ is statistically significant. This is a Wald-type $\chi^{2}$ test: under regularity conditions and correct specification, the stacked elements $\sqrt{n}\;\left(B_{n}-A_{n}\right)$ are asymptotically *jointly* normal with mean zero, so the associated quadratic form converges to $\chi_{d(d+1)/2}^{2}$ [84]. This section introduces additional misspecification metrics and presents an information-theoretic interpretation of the misalignment score of Vrugt et al. [8]. These measures complement commonly used model evaluation techniques such as residual diagnostics, which assess the validity of likelihood assumptions about variance, distributional form, and dependence structure [11,57]. In contrast, our proposed metrics do not rely explicitly on residual behavior or associated goodness-of-fit statistics. Instead, they assess misspecification implicitly through structural features, specifically, the alignment between the sensitivity and variability matrices, $A_{n}$ and $B_{n}$, which reflect both the model’s internal dynamics and its interaction with the data. These diagnostics help guide model selection and improvement, and serve as a safeguard against overconfidence in model-based inference, particularly in applications where structural model error is difficult to detect or eliminate through residual analysis alone.

In theory, the proposed metrics can be evaluated at any θ∈Θ provided that the local sensitivity matrix is nonsingular (ideally positive definite) and the local variability matrix is positive semidefinite. In practice, we presuppose calibration and report the metrics at the MAP estimate ${\hat{\theta }}_{*}$, using the naive $\Sigma_{n}^{\mathrm{naive}}$ and sandwich $\Sigma_{n}^{\mathrm{sand}}$ variances computed from $A_{n}$ and $B_{n}$. Without calibration, these quantities are not as meaningful.

### 9.1. Relative Entropy

Let $F=N_{d}\left({\hat{\theta }}_{*},\Sigma_{n}^{\mathrm{sand}}\right)$ and $P=N_{d}\left({\hat{\theta }}_{*},\Sigma_{n}^{\mathrm{naive}}\right)$ denote the *d*-variate normal distributions of the sandwich and naive variance estimators, respectively. The Kullback and Leibler [19] divergence $d_{\mathrm{KL}}\left(P,F\right)$ of *F* and *P* equals (derivation in Appendix B of Vrugt [85])

$$\begin{array}{cc} d_{\mathrm{KL}}\left(N_{d}\left({\hat{\theta }}_{*},\Sigma_{n}^{\mathrm{naive}}\right),N_{d}\left({\hat{\theta }}_{*},\Sigma_{n}^{\mathrm{sand}}\right)\right) & =\frac{1}{2}\left[\log\left(\left|{\left(\Sigma_{n}^{\mathrm{naive}}\right)}^{-1}\Sigma_{n}^{\mathrm{sand}}\right|\right)+\mathrm{tr}\left({\left(\Sigma_{n}^{\mathrm{sand}}\right)}^{-1}\Sigma_{n}^{\mathrm{naive}}\right)-d\right]. \end{array}$$

This statistical distance between the sandwich and naive posterior distributions is also known as the relative entropy from *P* to *F*, and equals the multivariate divergence score proposed by Dawid and Sebastiani [56] for identical means. We can admit the bread and meat matrices

(32) $$\begin{array}{cc} d_{\mathrm{xx}}\left(N_{d}\left({\hat{\theta }}_{*},\Sigma_{n}^{\mathrm{naive}}\right),N_{d}\left({\hat{\theta }}_{*},\Sigma_{n}^{\mathrm{sand}}\right)\right) & =\frac{1}{2}\left[\log\left(\left|nA_{n}\frac{1}{n}A_{n}^{-1}\beta_{n}A_{n}^{-1}\right|\right)+\mathrm{tr}\left(nA_{n}\beta_{n}^{-1}A_{n}\frac{1}{n}A_{n}^{-1}\right)-d\right]\\ \; & =\frac{1}{2}\log(|\beta_{n}A_{n}^{-1}\left|\right)+\frac{1}{2}\mathrm{tr}\left(A_{n}\beta_{n}^{-1}\right)-\frac{d}{2}. \end{array}$$

This divergence score is *strictly proper*, meaning that $d_{\mathrm{xx}}\left(P,F\right)$ is nonnegative and zero only if P=F, thus, $\Sigma_{n}^{\mathrm{naive}}=\Sigma_{n}^{\mathrm{sand}}$. The greater the misalignment between the sensitivity and variability matrices, the larger the value of $d_{\mathrm{xx}}\left(P,F\right)$. The subscript ‘xx’ is intentionally used as a neutral placeholder, and we leave the formal naming of this divergence to future users or the broader research community. The misalignment score of Equation (32) is particularly well suited for applications in machine learning, where the sensitivity (bread) matrix, $A_{n}$ and variability (meat) matrix $\beta_{n}$ can often be obtained “for free” as by-products of automatic differentiation. The misalignment score satisfies $d_{\mathrm{xx}}\left(P,F\right)=H\left(P,F\right)-H\left(P\right)$, where

$$\begin{array}{cc} H(P,F) & =\frac{1}{2}\log\left({\left(2\pi \right)}^{d}\left|\frac{1}{n}A_{n}^{-1}\beta_{n}A_{n}^{-1}\right|\right)+\frac{1}{2}\mathrm{tr}\left(nA_{n}\beta_{n}^{-1}A_{n}\frac{1}{n}A_{n}^{-1}\right)\left(\left|\frac{1}{n}A_{n}^{-1}\beta_{n}A_{n}^{-1}\right|=n^{-d}|A_{n}^{-1}{|}^{2}\;\left|\beta_{n}\right|\right)\\ \; & =\frac{d}{2}\log\left(2\pi \right)-\frac{d}{2}\log\left(n\right)-\log(|A_{n}\left|\right)+\frac{1}{2}\log(|\beta_{n}\left|\right)+\frac{1}{2}\mathrm{tr}\left(A_{n}\beta_{n}^{-1}\right), \end{array}$$

is the cross-entropy between the *d*-variate normal naive and sandwich distributions and

$$\begin{array}{cc} H(P) & =\frac{1}{2}\log\left({\left(2\pi e\right)}^{d}\left|\frac{1}{n}A_{n}^{-1}\right|\right)\\ \; & =\frac{d}{2}+\frac{d}{2}\log\left(2\pi \right)-\frac{d}{2}\log\left(n\right)-\frac{1}{2}\log(|A_{n}\left|\right), \end{array}$$

is the differential entropy of the multinormal naive distribution. This formulation highlights the misalignment score’s role as an information-theoretic measure of misspecification and closes the circle with our earlier work on probabilistic model evaluation [85].

The misalignment score of Equation (32) can be directly compared across models with differing numbers of parameters. It should yield the same model ranking as the logarithmic score, or expected log predictive density

(33) $$S_{\mathrm{LS}}\left(P,\omega \right)=\frac{1}{n}\sum_{t=1}^{n}\log\left(f_{P_{t}}\left(\omega_{t}|M\right)\right)$$

where $P=\{P_{1},\ldots ,P_{n}\}$ are the posterior predictive distributions under model M for the naive estimator. This forms the basis of model selection criteria such as the widely applicable information criterion or WAIC and leave-one-out cross-validation [86,87]. If so desired, the misalignment score can be normalized to explicitly account for the number of model parameters *d*. This yields a per-dimension misalignment score ${\bar{d}}_{\mathrm{xx}}\left(P,F\right)$

(34) $$\begin{array}{cc} {\bar{d}}_{\mathrm{xx}}\left(N_{d}\left({\hat{\theta }}_{*},\Sigma_{n}^{\mathrm{naive}}\right),N_{d}\left({\hat{\theta }}_{*},\Sigma_{n}^{\mathrm{sand}}\right)\right) & =\frac{1}{2}d^{-1}\log(|\beta_{n}A_{n}^{-1}\left|\right)+\frac{1}{2}d^{-1}\mathrm{tr}\left(A_{n}\beta_{n}^{-1}\right)-\frac{1}{2}, \end{array}$$

which can be compared across an ensemble of candidate models with differing number of parameters.

### 9.2. Fréchet Distance

The misalignment between $F=N_{d}\left({\hat{\theta }}_{*},\Sigma_{n}^{\mathrm{sand}}\right)$ and $P=N_{d}\left({\hat{\theta }}_{*},\Sigma_{n}^{\mathrm{naive}}\right)$ can also be quantified by the Earth Mover’s or Fréchet distance [88,89]

$$\begin{array}{cc} d_{F}\left(N_{d}\left({\hat{\theta }}_{*},\Sigma_{n}^{\mathrm{naive}}\right),N_{d}\left({\hat{\theta }}_{*},\Sigma_{n}^{\mathrm{sand}}\right)\right) & =\sqrt{\mathrm{tr}\left(\Sigma_{n}^{\mathrm{naive}}+\Sigma_{n}^{\mathrm{sand}}-2{\left(\Sigma_{n}^{\mathrm{naive}}\Sigma_{n}^{\mathrm{sand}}\right)}^{1/2}\right)}\\ \; & =n^{-1/2}\sqrt{\mathrm{tr}\left(A_{n}^{-1}+A_{n}^{-1}\beta_{n}A_{n}^{-1}-2{\left(A_{n}^{-2}\beta_{n}A_{n}^{-1}\right)}^{1/2}\right)}. \end{array}$$

This distance is widely used in machine learning to compare the distribution of generated images from a model against the distribution of real images. Smaller values indicate greater similarity between distributions, with $d_{F}=0$ corresponding to perfect agreement.

### 9.3. Frobenius Norm

An alternative diagnostic metric is the Frobenius norm of the difference between the naive and sandwich variance estimators

(35) $$\begin{array}{cc} {∥P-F∥}_{F} & =\sqrt{\sum_{i=1}^{d}\sum_{j=1}^{d}{\left(\Sigma_{n,ij}^{\mathrm{naive}}-\Sigma_{n,ij}^{\mathrm{sand}}\right)}^{2}}=\frac{1}{n}{∥A_{n}^{-1}\left(I_{d}-\beta_{n}A_{n}^{-1}\right)∥}_{F}, \end{array}$$

where smaller values indicate better model specification, and a value of zero is ideal. Larger values imply greater degrees of misspecification. Alternatively, one can compare the observed Fisher ${\hat{I}}_{n}$ and Godambe ${\hat{G}}_{n}$ information matrices at the MAP parameter values

$$\begin{array}{cc} \; & {∥{\hat{I}}_{n}-{\hat{G}}_{n}∥}_{F}=n\;{∥A_{n}\left(I_{d}-\beta_{n}^{-1}A_{n}\right)∥}_{F}. \end{array}$$

This yields qualitatively similar conclusions but on a different scale. Further examination of the Fisher-Godambe discrepancy offers valuable insight into the nature and extent of model misspecification, particularly under different modeling assumptions and data sets.

### 9.4. Herfindahl Index

Under correct specification, the theoretical precision matrix M≡A*1/2−1B*−11/2A*1/2−1 defined in Remark 8 on Page 17 will equal an identity matrix $I_{d}$. Then, the eigenvalues ${\underline{\lambda }}_{1},\ldots ,{\underline{\lambda }}_{d}$ of $M=I_{d}$ will equal one. Suppose we normalize the *d* eigenvalues of M

$${\underline{\lambda }}_{i,n}=\frac{{\underline{\lambda }}_{i}}{\sum_{j=1}^{d}{\underline{\lambda }}_{j}},\;i=1,\ldots ,d,$$

then the *Herfindahl index H*

$$H=\sum_{i=1}^{d}{\underline{\lambda }}_{i,n}^{2}.$$

is a measure of how dispersed or concentrated the eigenvalues are across the parameter space. This metric is commonly used in economics as a scalar summary of the variance concentration in the principal components of a covariance matrix [90,91]. Under correct specification, all normalized eigenvalues attain a value of $d^{-1}$ and H=1/d. This is the lowest possible value for the Herfindahl index and indicates maximum variance uniformity across dimensions. The maximum value H=1 is reached when all variability is concentrated in a single direction. Higher Herfindahl indices, thus, imply that most of the uncertainty is concentrated along a few dimensions of the parameter space, potentially indicating *ill-conditioning* or *overfitting*. Large differences between the Herfindahl indices of $\Sigma_{n}^{\mathrm{naive}}$ and $\Sigma_{n}^{\mathrm{sand}}$ signal model misspecification. In particular, the naive estimator may imply uniformly distributed uncertainty, whereas the sandwich estimator captures the anisotropic structure introduced by model error.

For the identity matrix $I_{d}$ and diagonal matrix $\mathrm{diag}({\underline{\lambda }}_{1},\ldots ,{\underline{\lambda }}_{d})$ we find

$$d_{\mathrm{KL}}(N_{d}\left(0,I_{d}\right),N_{d}(0,\mathrm{diag}\left({\underline{\lambda }}_{1},\ldots ,{\underline{\lambda }}_{d}\right)))=\frac{1}{2}\sum_{i=1}^{d}\left\{{\underline{\lambda }}_{i}^{-1}-1+\log\left({\underline{\lambda }}_{i}\right)\right\},$$

and the reverse KL-divergence

$$d_{\mathrm{KL}}(N_{d}\left(0,({\underline{\lambda }}_{1},\ldots ,{\underline{\lambda }}_{d})\right),N_{d}\left(0,I_{d}\right))=\frac{1}{2}\sum_{i=1}^{d}\left\{{\underline{\lambda }}_{i}-1-\log\left({\underline{\lambda }}_{i}\right)\right\}.$$

The symmetrized KL divergence is $\frac{1}{4}\sum_{i=1}^{d}\left\{{\underline{\lambda }}_{i}-1+{\underline{\lambda }}_{i}^{-1}\right\}$.

Thus, the Herfindahl index adds to the suite of diagnostics by providing an interpretable scalar summary of the effective dimensionality of the parameter uncertainty. This makes it particularly useful for comparing models of varying complexity or visualizing behavior along a complexity–regularization trade-off. A related measure is the sample variance of ${\underline{\lambda }}_{i}$’s or $s_{\underline{\lambda }}^{2}=\left(\sum_{i=1}^{d}{\underline{\lambda }}_{i}^{2}-dm_{\underline{\lambda }}^{2}\right)/\left(d-1\right)$, where $m_{\underline{\lambda }}$ is the sample mean of ${\underline{\lambda }}_{1},\ldots ,{\underline{\lambda }}_{d}$.

The misspecification diagnostics introduced here serve as a companion to predictive model selection criteria such as the Akaike information criterion (AIC; [92]), the Bayesian information criterion (BIC; [93]), and the WAIC [86]. Whereas these criteria rank models by expected predictive performance under a correctly specified likelihood, our measures assess whether that assumption is credible by quantifying the alignment between the sensitivity and variability matrices. Low misalignment scores support the use of AIC/BIC/WAIC with greater confidence. Large discrepancies warn that their penalties may understate uncertainty and yield overconfident rankings. In practice, the proposed diagnostics can be used to screen out poorly specified models before comparing predictive performance.

In this study, we applied the proposed sandwich-adjusted MCMC simulation method to a collection of discharge data sets and a suite of hydrologic models of varying complexity. For each case, we computed the *omnibus* scalar *k* introduced by Pauli et al. [31]. In nearly all applications, the estimated *k* deviated markedly from the value of unity expected under correct model specification, indicating substantial misspecification across all models. Although more complex models with larger parameter dimensionality *d* often yielded higher omnibus values, the relationship was not strictly monotonic. This suggests that model structure rather than dimensionality alone plays a critical role in determining specification quality. These findings highlight the practical value of our method for assessing model adequacy and underscore the need for further research on the interplay between model complexity, misalignment scores, other misspecification-based diagnostics, and commonly used residual-based measures of predictive performance.

## 10. Summary and Conclusions

Frequentist and Bayesian methods are widely used for standard tasks such as statistical inference and hypothesis testing, as well as for more specific tasks including model training (calibration) and prediction (forecasting). In an earlier article, we demonstrated a critical flaw in both maximum likelihood (ML) and Bayesian approaches under model misspecification. Contrary to common teaching and statistical practice, the asymptotic covariance matrix of the ML parameter estimates, $\Sigma_{n}$, does not equal the inverse of the observed Fisher information matrix, ${\hat{I}}_{n}$. Instead, it corresponds to the sandwich variance matrix Σnsand=G^G^_n−1 where the observed Godambe information is defined as G^n=nAn−1Bn−1An−1. This Godambe matrix serves as the fundamental measure of data informativeness under model misspecification [8]. Here, $A_{n}$ and $B_{n}$ are sample averages of the sensitivity and variability matrices, respectively, for *n* data points $\omega_{1},\ldots ,\omega_{n}$, evaluated at the ML parameter estimates ${\hat{\theta }}_{*}$.

The goals of this paper were three-fold. First, we reviewed and examined three existing methods for producing asymptotically valid sandwich posterior distributions. The first method, known as the open-faced sandwich (OFS) adjustment of Shaby [25], applies direction-specific dilations along the principal axes of the samples of the naive posterior distribution, Σnnaive=I^I^_n−1 to align its local curvature around the MAP estimator ${\hat{\theta }}_{*}$ with that of the sandwich variance matrix $\Sigma_{n}^{\mathrm{sand}}$. Specifically, naive posterior samples $\theta_{\left(1\right)},\ldots ,\theta_{\left(M\right)}$ are centered on the MAP estimator and pre-multiplied by the matrix Ψn=An−11/2Bn1/2An1/2 to yield OFS-adjusted samples $\theta_{\left(j\right)}^{\mathrm{ofs}}={\hat{\theta }}_{*}+\Psi_{n}\left(\theta_{\left(j\right)}-{\hat{\theta }}_{*}\right)$ for all j=1,…,M. This a-posteriori transformation is computationally efficient and simple to implement, but it does not guarantee a fully accurate characterization of the sandwich distribution.

The second method, magnitude-adjusted MCMC of Pauli et al. [31], aligns the posterior with the sandwich distribution by raising the likelihood function $L_{n}\left(\theta \right)$ to a scalar power 0<k<1, known as the *omnibus* scalar. The scalar *k* is chosen such that the estimated information matrices, $A_{n}$ and $B_{n}$ satisfy the information identity $kA_{n}=k^{2}B_{n}$. This power-likelihood approach, denoted $L_{n}^{k}\left(\theta \right)$, effectively tempers the learning rate and produces posterior samples whose covariance is inversely proportional to the observed Godambe information G^n=nAn−1Bn−1An−1. While this method is computationally attractive, it applies a single scalar *k* to all *d* parameters. Consequently, it yields exact results only when the local shape of $L_{n}\left(\theta \right)$ near ${\hat{\theta }}_{*}$ is approximately quadratic. If the posterior exhibits anisotropy (directional variation) or asymmetry, a single scalar *k* may distort the geometry of the true sandwich distribution, suggesting the need for dimensionality-specific scaling.

The third method, curvature-adjusted MCMC of Ribatet et al. [24], modifies the sampling procedure by evaluating the likelihood $L_{n}\left(\theta \right)$ at an affine transformation of the candidate points. Specifically, each proposed point $\theta_{p}$ is transformed to $\theta_{p}^{\mathrm{ca}}={\hat{\theta }}_{*}+C_{n}\left(\theta_{p}-{\hat{\theta }}_{*}\right)$, where the tuning matrix is defined as $C_{n}=B_{n}^{-1/2}A_{n}^{1/2}$. This transformation effectively enforces the sandwich covariance on the MCMC samples, ensuring that the sampled chains reflect the curvature implied by the observed Godambe information. However, this method has important limitations. The matrix square roots $A_{n}^{1/2}$ and $B_{n}^{1/2}$ are not uniquely defined unless the log-likelihood $L_{n}\left(\theta \right)$ is exactly quadratic in the neighborhood of ${\hat{\theta }}_{*}$. As a result, the transformation can induce arbitrary rotations of the posterior ellipsoids, which may misrepresent the true directional asymmetries of the sandwich distribution. Moreover, curvature-adjusted MCMC does not respect parameter bounds, and care must be taken to ensure that proposed candidate points lie within the feasible domain.

As the second objective of this paper, we presented the theoretical foundation of a kernel adjustment method for sandwich-adjusted MCMC simulation. This approach is similar in spirit as magnitude-adjusted MCMC but employs a scaled log-likelihood function of the form $L_{n}^{p}\left(\theta |\lambda \right)=\lambda \left(\theta \right)\left\{L_{n}\left(\theta \right)-L_{n}\left({\hat{\theta }}_{*}\right)\right\}$ centered at the maximum a posteriori (MAP) estimator ${\hat{\theta }}_{*}$ and governed by a nonconstant, parameter-dependent power λ(θ)>0. This dynamic learning rate is defined as $\lambda \left(\theta \right)=\left\{{\left(\theta -{\hat{\theta }}_{*}\right)}^{⊤}{\hat{G}}_{n}\left(\theta -{\hat{\theta }}_{*}\right)\right\}/\left\{{\left(\theta -{\hat{\theta }}_{*}\right)}^{⊤}nA_{n}\left(\theta -{\hat{\theta }}_{*}\right)\right\}$ and is typically less than one as the sandwich or Godambe information ${\hat{G}}_{n}$ is generally smaller in magnitude than $nA_{n}$ under correct specification. Thus, the power λ(θ)>0 flattens the posterior surface in regions where the observed information exceeds the robust information, reducing overconfidence and improving robustness under model misspecification. Note that under correct specification, ${\hat{G}}_{n}={\hat{I}}_{n}=nA_{n}$, we yield a unit learning rate for all θ and recover the naive covariance matrix Σnnaive=I^I^_n−1. The learning rate λ(θ) facilitates the construction of robust Bayesian credible regions under misspecification without requiring matrix square roots or eigen decompositions.

We demonstrated the four different sandwich adjustment methods by application to three case studies of increasing complexity. The first two case studies focus attention to simple statistical models with analytically tractable derivatives and offer deeper insight into the differences between naive and sandwich variance estimators under a wrong parameterization (study 1) and inadequate parametric form (study 2) of the data-generating process. The naive variance estimator fails to account for these discrepancies and leads to overconfident inference. The first study confirmed that the frequentist sandwich variance estimator produces asymptotically valid confidence intervals. The second study demonstrated that the OFS adjustment of Shaby [25] increased the spread of the naive posterior samples, but the resulting credible regions did not achieve the theoretical sandwich coverage probabilities. In contrast, parameter credible regions obtained using magnitude-, curvature-, and sandwich-adjusted MCMC simulation were in close agreement with one another and almost attained the expected coverage. This deviation was due to the assumption of symmetry in constructing the 100(1−α)% credible intervals. Altogether, the first two studies confirmed that the sandwich estimator yields asymptotically valid “robust standard errors” even when $L_{n}\left(\theta \right)$ is wrongly parameterized or misspecified.

The third and final study applied the proposed methods to a rainfall–discharge simulation using the Xinanjiang watershed model. The results confirmed that traditional MCMC methods tend to produce overly narrow credible intervals for both model parameters and simulated outputs. This well-known phenomenon of overconditioning arises from the incorrect assumption of a well-specified model. Magnitude-, curvature-, and sandwich-adjusted MCMC simulation relax this assumption and yield substantially larger credible regions of the Xinanjiang model parameters and simulated streamflows. Our proposed method with a dynamic learning rate yields more robust Bayesian credible intervals than magnitude-adjusted MCMC sampling and does not suffer problems with nonuniqueness of principal matrix square roots as in curvature-adjusted MCMC simulation. All three methods, magnitude-, curvature-, and sandwich-adjusted MCMC methods require sample estimates $A_{n}$ and $B_{n}4$ of the sensitivity and variability matrices, respectively, along with an estimate of ${\hat{\theta }}_{*}$. In principle, the sampled chains from sandwich-adjusted MCMC converge rapidly to the sandwich distribution, since the chains are initialized in the vicinity of the MAP solution. However, this approach will always incur a greater computational cost than naive Bayesian methods.

As third and final objective of this paper, we presented an information-theoretic interpretation of the alignment score proposed by Vrugt et al. [8]. This *strictly proper* score measures the concordance of the bread and meat matrices and can be decomposed into a cross-entropy and differential entropy term. The misalignment score guides model improvement and enables direct comparison across models with different numbers of parameters, supporting model selection. We also explored others scalar measures for the degree of model misspecification. These include the Earth Mover’s distance, Frobenius norm, and Herfindahl index. Each measure captures different aspects of the discrepancy between naive and sandwich variance estimators caused by model misspecification. The Herfindahl index also offers a scalar diagnostic of the effective dimensionality of posterior uncertainty and serves as a useful diagnostic of anisotropy and concentration in the naive and sandwich variance estimators. Application of these measures to a suite of hydrologic models confirmed that all models were substantially misspecified. This analysis further showed that an increased model complexity does not guarantee better specification. Further research is warranted on the interplay between model complexity, the proposed misalignment score, other misspecification-based diagnostics, and widely used residual-based measures of predictive performance.

## Figures and Tables

**Table 1:** Normal distribution model $N(m,s^{2})$: ML estimate of $\hat{m}$, and associated values of the information matrices $A_{n}$ and $B_{n}$, *omnibus* scalar $\hat{k}$, and naive and sandwich variance estimators using the normal log-likelihood function $L_{n}^{n}\left(m|s^{2}\right)$ in Equation (5) with $s^{2}=2$, $s^{2}=1$, and $s^{2}=0.5$. Tabulated values are an average of $M=10^{4}$ different realizations of $\omega_{1},\ldots ,\omega_{100}$ sampled from the data generating process. Standard deviations are listed between parenthesis.

$s^{2}$	$\hat{m}$	$A_{n}$	$B_{n}$	$\hat{k}$	$\Sigma_{n}^{\mathrm{naive}}$	$\Sigma_{n}^{\mathrm{sand}}$
2	−0.001 (0.099)	0.500 (0.000)	0.247 (0.036)	2.041 (0.298)	0.020 (0.000)	0.010 (0.001)
1	−0.001 (0.099)	1.000 (0.000)	0.990 (0.143)	1.021 (0.149)	0.010 (0.000)	0.010 (0.001)
0.5	−0.001 (0.099)	2.000 (0.000)	3.959 (0.568)	0.516 (0.089)	0.005 (0.000)	0.010 (0.001)

**Table 2:** Coverage in % of the *true* mean μ of the data generating process $N(\mu ,\sigma^{2})$ for 100(1−α)% confidence intervals of the ML estimate $\hat{m}$ under the normal distribution model $N(m,s^{2})$ with $s^{2}=2$, $s^{2}=1$, and $s^{2}=1/2$ using the naive and sandwich variance estimators. Results are based on the code in Appendix C using $M=10^{3}$ trials with μ=0, $\sigma^{2}=1$, and sample size n=100.

α	$s^{2}=2$		$s^{2}=1$		$s^{2}=0.5$		Theoretic Coverage
	$\Sigma_{n}^{\mathrm{naive}}$	$\Sigma_{n}^{\mathrm{sand}}$	$\Sigma_{n}^{\mathrm{naive}}$	$\Sigma_{n}^{\mathrm{sand}}$	$\Sigma_{n}^{\mathrm{naive}}$	$\Sigma_{n}^{\mathrm{sand}}$	
0.01	100.00	98.70	98.90	98.70	93.00	98.70	99%
0.05	99.10	94.80	94.70	94.80	83.10	94.80	95%
0.10	97.90	90.00	90.30	90.00	75.70	90.00	90%
0.20	92.70	79.20	79.20	79.20	64.00	79.20	80%
0.30	84.90	71.60	71.40	71.60	53.40	71.60	70%
0.40	75.90	60.10	60.40	60.00	44.00	60.00	60%
0.50	67.00	49.70	49.80	49.80	34.50	49.80	50%

**Table 3:** Coverage in % of the *true* mean μ=a·b of the data generating process G(a,b) for 100(1−α)% confidence intervals of the ML estimate $\hat{\mu }$ under the exponential distribution E(μ) using the naive and sandwich variance estimators. Results are based on $M=10^{4}$ trials with a=0.5, b=0.2 and sample size n=100. The Matlab code is given in Appendix D.

Method/Coverage	α=0.01	α=0.05	α=0.1	α=0.2	α=0.3	α=0.4	α=0.5
	99%	95%	90%	80%	70%	60%	50%
Naive estimator	92.98	83.56	75.62	63.71	54.22	45.26	37.19
Sandwich estimator	97.71	93.71	88.46	79.04	68.73	59.38	49.72

**Table 4:** Coverage (in %) of the *true* mean μ=a·b of the data-generating process G(a,b) for the 100(1−α)% credible intervals of parameter $\hat{\mu }$ of E(μ) obtained from MCMC simulation (=naive MCMC), OFS-adjusted naive posterior samples, and magnitude-, curvature-, and sandwich-adjusted DREAM_(ZS)_ algorithms. Results are based on $M=10^{4}$ trials with a=0.5, b=0.2 and n=100.

Method	Likelihood	α=0.01	α=0.05	α=0.1	α=0.2	α=0.3	α=0.4	α=0.5
		99%	95%	90%	80%	70%	60%	50%
Naive MCMC	$L_{n}\left(\mu \right)$	92.33	83.11	75.30	63.69	53.38	44.64	36.38
**OFS adjustment **	$L_{n}\left(\mu \right)$	94.93	90.69	85.98	76.36	66.57	57.04	47.46
**Algorithm 1**	$L_{n}{\left(\mu \right)}^{k}$	98.39	93.67	88.98	78.61	68.89	58.93	48.92
**Algorithm 2**	$L_{n}^{\mathrm{ca}}\left(\mu \right)$	98.41	93.69	88.86	78.87	68.80	59.14	48.86
**Algorithm 3**	$L_{n}^{p}\left(\mu |\lambda \right)$	98.49	93.78	88.96	78.58	68.96	59.44	49.31

**Table 5:** Total sensitivity $A_{n}^{s}$ and variability $\beta_{n}^{s}$ matrices of the Xinanjiang model parameters and nuisance variables ν and ξ of the Student *t* likelihood.

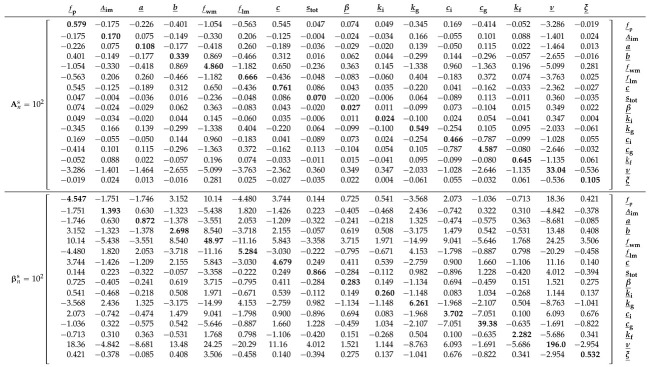

**Figure A1:**
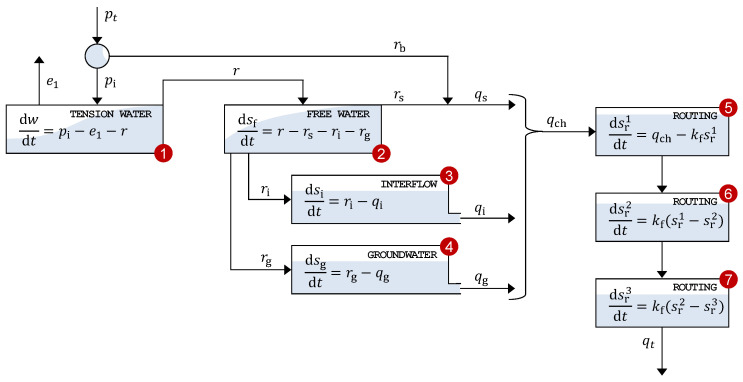
Schematic illustration of the Xinanjiang conceptual watershed model. Blue boxes labeled in red are fictitious control volumes that govern the rainfall-runoff transformation. The model includes seven state variables, including the free *w* and tension $s_{t}$ water storages of the upper soil layer, interflow $s_{i}$ and groundwater $s_{g}$ reservoirs and water levels $s_{r}^{1}$, $s_{r}^{2}$, and $s_{r}^{3}$ of the routing reservoirs. Fluxes (arrows) describe water movement into and out of compartments: precipitation ($p_{t}$), runoff from impervious areas ($r_{b})$, infiltration ($p_{i}$), surface runoff from the contributing free area ($r_{s}$), evaporation ($e_{1}$), runoff (*r*), interflow ($r_{i}$), baseflow ($r_{g}$), delayed interflow ($q_{i}$), delayed baseflow ($q_{g}$), and surface runoff ($q_{s}$). These fluxes are computed as follows, $r_{b}=A_{\mathrm{im}}p_{t}$, $p_{i}=\left(1-A_{\mathrm{im}}\right)p_{t}$, $r_{s}=r\left\{1-{\left(1-s_{f}/s_{\max}\right)}^{\beta }\right\}$, $e_{1}=e_{\mathrm{pan}}$ if w>lm, $e_{1}=\left(w/\mathrm{lm}\right)e_{\mathrm{pan}}$ if c·lm≤w≤lm otherwise $e_{1}=c\;\cdot \;e_{\mathrm{pan}}$, $r=p_{i}\left\{{\left(0.5-a\right)}^{\left(1-b\right)}{\left(w/w_{\max}\right)}^{b}\right\}$ if $(w/w_{\max})\leq 0.5-a$ and $r=p_{i}\left\{1-{\left(0.5+a\right)}^{\left(1-b\right)}{\left(1-w/w_{\max}\right)}^{b}\right\}$ otherwise, $r_{i}=k_{i}s_{f}\left\{1-{\left(1-s_{f}/s_{\max}\right)}^{\beta }\right\}$, $r_{g}=k_{i}s_{g}\left\{1-{\left(1-s_{f}/s_{\max}\right)}^{\beta }\right\}$, $q_{i}=c_{i}s_{i}$, $q_{g}=c_{g}s_{g}$, and $q_{s}=r_{b}+r_{s}$, where $e_{\mathrm{pan}}=f_{p}e_{p}$ is pan evaporation, $e_{p}$ denotes the potential evapotranspiration, $w_{\max}=f_{\mathrm{wm}}s_{\mathrm{tot}}$ is the maximum tension water depth, $s_{\max}=\left(1-f_{\mathrm{wm}}\right)s_{\mathrm{tot}}$ is the maximum free water depth, $\mathrm{lm}=f_{\mathrm{lm}}w_{\max}$ is the tension water threshold for evaporation change and $f_{p}$, $A_{\mathrm{im}}$, *a*, *b*, $s_{\mathrm{tot}}$, $f_{\mathrm{wm}}$, $f_{\mathrm{lm}}$, *c*, β, $k_{i}$, $k_{g}$, $c_{i}$, $c_{g}$ and $k_{f}$ are free parameters. Total channel inflow $q_{\mathrm{ch}}=q_{s}+q_{i}+q_{g}$ is routed through three linear reservoirs (with identical recession constant $k_{f}$) and produces streamflow at watershed outlet, $q_{t}=k_{f}s_{r}^{3}$.

## Data Availability

The theory, methodology, and case studies presented in this paper are part of DREAM-Suite, a MATLAB–Python software package for Bayesian model training, evaluation, and diagnostics [28]. This software is available at https://github.com/jaspervrugt/dream-suite (accessed on 3 September 2025).

## Abbreviations

The following abbreviations are used in this manuscript:

MCMC	Markov Chain Monte Carlo
MH	Metropolis–Hastings
DREAM_(ZS)_	DiffeRential Evolution Adaptive Metropolis
ML	Maximum Likelihood
MAP	Maximum A Posteriori
OFS	Open-Face Sandwich
CAMH	Curvature-Adjusted Metropolis–Hastings
SAMH	Sandwich-adjusted Metropolis–Hastings
DREAM-Suite	Matlab-Python software package for Bayesian training, evaluation and diagnostics

## Appendix A

In this Appendix, we demonstrate that an equivalent expression for $B_{*}$ is the variance of the score ${\dot{L}}_{\omega }^{n}\left(m\right)$ at m=μ.

The normal log-likelihood $L_{\omega }^{n}\left(m|s^{2}\right)$ for a single datum ω is given by

$$\begin{array}{c} L_{\omega }^{n}\left(m|s^{2}\right)=\frac{1}{2}\log\left(2\pi s^{2}\right)-\frac{1}{2}s^{-2}{\left(\omega -m\right)}^{2}, \end{array}$$

with first derivative

$$\begin{array}{c} {\dot{L}}_{\omega }^{n}\left(m|s^{2}\right)=s^{-2}\left(\omega -m\right), \end{array}$$

as shown in Equation (6).

The expected value of the score is equal to

$$\begin{array}{cc} E_{\omega }\left[{\dot{L}}_{\omega }^{n}\left(m|s^{2}\right)\right] & =E_{\omega }\left[s^{-2}\left(\omega -m\right)\right]\\ \; & =s^{-2}\left(E_{\omega }\left[\omega \right]-E_{\omega }\left[m\right]\right)\\ \; & =s^{-2}\left(\mu -m\right), \end{array}$$

where $\mu =E_{\omega }\left[\omega \right]$ is the population mean. The variance of the score becomes

$$\begin{array}{cc} \mathrm{Var}[{\dot{L}}_{\omega }^{n}\left(m|s^{2}\right)] & =E_{\omega }\left[\left({\dot{L}}_{\omega }^{n}\left(m|s^{2}\right)-E_{\omega }\left[{\dot{L}}_{\omega }^{n}\left(m|s^{2}\right)\right]\right){\left({\dot{L}}_{\omega }^{n}\left(m|s^{2}\right)-E_{\omega }\left[{\dot{L}}_{\omega }^{n}\left(m|s^{2}\right)\right]\right)}^{⊤}\right]\\ \; & =E_{\omega }\left[{\left(s^{-2}\left(\omega -m\right)-s^{-2}\left(\mu -m\right)\right)}^{2}\right]\\ \; & =E_{\omega }\left[{\left(s^{-2}\left(\omega -\mu \right)\right)}^{2}\right]\\ \; & =s^{-4}E_{\omega }\left[{\left(\omega -\mu \right)}^{2}\right]. \end{array}$$

At the likelihood maximum m=μ, we yield the following expression

$$\begin{array}{c} \mathrm{Var}\left[{\dot{L}}^{n}\omega \left(m|s^{2}\right)\right]=s^{-4}\sigma^{2}, \end{array}$$

which corresponds to $B_{*}$ in Equation (8).

## Appendix B

In this Appendix, we present an asymptotic proof of convergence of sandwich adjusted MCMC simulation.

**Theorem A1.** 
*Let $\theta_{*}\in R^{d}$ denote the vector of pseudo-true parameter values and ${\hat{\theta }}_{*}$ the MLE (or MAP under a uniform prior). Assume the prior is continuous and strictly positive at $\theta_{*}$. Suppose the log-likelihood $L_{n}\left(\theta \right)=\log\left(L_{n}\left(\theta \right)\right)$ is twice continuously differentiable in a neighborhood of $\theta_{*}$, and define*

$$A_{*}=-\frac{1}{n}E\left[\nabla^{2}L_{n}\left(\theta_{*}\right)\right],\;B_{*}=\frac{1}{n}E\left[\nabla L_{n}\left(\theta_{*}\right)\nabla^{⊤}L_{n}\left(\theta_{*}\right)\right],$$

*with $A_{*}$ and $B_{*}$ positive definite. Consider the power log-likelihood of Equation (21)*

(A1)
$$L_{n}^{p}\left(\theta |\lambda \right)=\lambda \left(\theta \right)\left[L_{n}\left(\theta \right)-L_{n}\left({\hat{\theta }}_{*}\right)\right],$$

*with learning rate of Equation (24)*

(A2)
λ(θ)=(θ−θ^*)⊤A*−1B*−1A*−1(θ−θ^*)(θ−θ^*)⊤A*−1(θ−θ^*),

*Then the corresponding target density*

$$\phi \left(\theta \right)\;\propto \;{\left(\frac{L_{n}\left(\theta \right)}{L_{n}\left({\hat{\theta }}_{*}\right)}\right)}^{\lambda (\theta )},$$

*is asymptotically equivalent to a d-variate normal distribution with mean ${\hat{\theta }}_{*}$ and d×d covariance matrix 1nA*−1B*−1A*−1.*

*Consequently, the stationary distribution of the sandwich-adjusted Metropolis–Hastings (SAMH) algorithm targeting ϕ is the sandwich-adjusted posterior in the large-sample limit.*

**Proof.** Let θ lie in a neighborhood of ${\hat{\theta }}_{*}$. A second-order Taylor expansion of the log-likelihood around ${\hat{\theta }}_{*}$ yields

$$L_{n}\left(\theta \right)-L_{n}\left({\hat{\theta }}_{*}\right)\;\approx \;-\frac{1}{2}n{\left(\theta -{\hat{\theta }}_{*}\right)}^{⊤}A_{*}\left(\theta -{\hat{\theta }}_{*}\right).$$

Substituting this into (A1) gives

$$L_{n}^{p}\left(\theta |\lambda \right)\;\approx \;-\frac{1}{2}n\lambda \left(\theta \right){\left(\theta -{\hat{\theta }}_{*}\right)}^{⊤}A_{*}\left(\theta -{\hat{\theta }}_{*}\right).$$

Using (A2)

Lnp(θ∣λ)≈−12n(θ−θ^*)⊤A*−1B*−1A*−1(θ−θ^*)(θ−θ^*)⊤A*−1(θ−θ^*)(θ−θ^*)⊤A*−1(θ−θ^*),

which simplifies to

Lnp(θ∣λ)≈−12n(θ−θ^*)⊤A*−1B*−1A*−1(θ−θ^*).

Therefore, under the assumed continuity and positivity of the prior at $\theta_{*}$

ϕ(θ)∝exp−12n(θ−θ^*)⊤A*−1B*−1A*−1(θ−θ^*),

which is *d*-variate normal with mean ${\hat{\theta }}_{*}$ and covariance matrix 1nA*−1B*−1A*−1. Hence, the SAMH target is asymptotically the sandwich-adjusted posterior. □

**Remark A1.** 

(i) *Informative priors. Let P(θ) be the log-prior. Under standard regularity conditions (prior density is positive and continuous near $\theta_{*}$), log-prior curvature is O(1) while the log-likelihood curvature is O(n), so the asymptotic covariance remains 1nA*−1B*−1A*−1, with $A_{*}$ and $B_{*}$ defined from the likelihood. Centering at the MAP estimator $\hat{\theta }$ improves finite-sample accuracy without altering the limit.*
(ii) *Sample estimators. As discussed in Section 5 we must replace $A_{*}$ and $B_{*}$ by consistent estimators $A_{n}$ and $B_{n}$ evaluated at ${\hat{\theta }}_{*}$. If $A_{*}=\mathrm{plim}A_{n}$ and $B_{*}=\mathrm{plim}B_{n}$ with positive definiteness, the same asymptotic result holds.*
(iii) *Finite-sample stability. When n is small or the model is ill-conditioned, eigenvalue clipping or ridge regularization of $A_{n}$ and $B_{n}$, robust covariance estimation for dependence [61], smoothing across iterations, and constraining λ(θ)∈[0,1] can improve stability without affecting the asymptotic target.*

## Appendix C

In this Appendix, we determine the bread and meat matrices $A_{*}$ and $B_{*}$, respectively, for the normal power likelihood function $L_{n}^{\mathrm{np}}\left(m|s^{2}\right)$ in Section 3.

The normal power log-likelihood $L_{n}^{\mathrm{np}}\left(m|s^{2}\right)$ is defined as

$$\begin{array}{cc} L_{n}^{\mathrm{np}}\left(m|s^{2}\right) & =kL_{n}^{n}\left(m|s^{2}\right)=-\frac{1}{2}nk\log\left(2\pi s^{2}\right)-\frac{1}{2}ks^{-2}\sum_{i=1}^{n}{\left(\omega_{i}-m\right)}^{2}, \end{array}$$

or for a single datum ω we can write

$$\begin{array}{c} L_{\omega }^{\mathrm{np}}\left(m|s^{2}\right)=-\frac{1}{2}k\log\left(2\pi s^{2}\right)-\frac{1}{2}ks^{-2}{\left(\omega -m\right)}^{2}. \end{array}$$

The first and second derivatives with respect to *m* are

$$\begin{array}{cc} {\dot{L}}_{\omega }^{\mathrm{np}}\left(m|s^{2}\right) & =\frac{d}{dm}L_{\omega }^{\mathrm{np}}\left(m|s^{2}\right)=ks^{-2}\left(\omega -m\right)\\ {\ddot{L}}_{\omega }^{\mathrm{np}}\left(m|s^{2}\right) & =\frac{d^{2}}{dm^{2}}L_{\omega }^{\mathrm{np}}\left(m|s^{2}\right)=-ks^{-2}. \end{array}$$

The sensitivity matrix (a scalar in this case) is

$$\begin{array}{cc} \; & A_{*}=E_{\omega }\left[-{\ddot{L}}_{\omega }^{\mathrm{np}}\left(m|s^{2}\right)\right]=E_{\omega }\left[ks^{-2}\right]=ks^{-2}. \end{array}$$

The variability matrix (also a scalar here) is

$$\begin{array}{cc} B_{*} & =E_{\omega }\left[{\dot{L}}_{\omega }^{\mathrm{np}}\left(m|s^{2}\right){\dot{L}}_{\omega }^{\mathrm{np}}{\left(m|s^{2}\right)}^{⊤}\right]\\ \; & =E_{\omega }\left[ks^{-2}\left(\omega -m\right)ks^{-2}\left(\omega -m\right)\right]\\ \; & =k^{2}s^{-4}E_{\omega }\left[{\left(\omega -m\right)}^{2}\right]. \end{array}$$

At the likelihood maximum m=μ, we have $E_{\omega }\left[{\left(\omega -m\right)}^{2}\right]⟶\sigma^{2}$ and hence $B_{*}=k^{2}s^{-4}\sigma^{2}$.

Suppose the normal distribution model is correctly specified, so that $s^{2}=\sigma^{2}$. Then, the variability matrix simplifies to $B_{*}=k^{2}s^{-2}$ and the naive and sandwich variances equal

(A3) Σ=1nA*−1=k−1s2/n,naivevariance,1nA*−1B*−1A*−1=k−1s2k2s−4σ2k−1s2/n=σ2/n,sandwichvariance.

The expression for the naive variance supports the widely held belief that applying an arbitrary power k>0 to the likelihood function provides a mechanism to control parameter uncertainty. This idea underlies the GLUE methodology of Beven and Binley [13]. Specifically, values of 0<k<1 inflate the confidence regions of the estimated parameters, while learning rates k>1 lead to a contraction of the “posterior” distribution of ${\hat{\theta }}_{*}$, thereby reducing parameter uncertainty.

Although elastic stretching of the likelihood function may appear to offer a pragmatic remedy for over-conditioning, it lacks rigorous theoretical support. This is clearly evidenced by the closed-form expression for the sandwich variance, in which the arbitrary power *k* cancels in the product A*−1B*−1A*−1. Consequently, under model misspecification, the learning rate *k* has no effect on the estimated parameter (and predictive) uncertainty.

This result provides important insight into the robust quantification of model and predictive uncertainty in the presence of outliers or structural model errors. The same conclusion was previously discussed in Vrugt et al. [8], to which interested readers are referred for further discussion.

### Computer Implementation

We implement the naive and sandwich variance estimators of Equation (A3) in Matlab. The code below generates Table 1 and Table 2. Built-in functions are highlighted with a low dash.

## Appendix D

In this Appendix, we derive analytic expressions for the empirical and theoretic naive and sandwich variances of the mean μ of the exponential distribution E(μ). This also leads to an expression for the *omnibus* scalar *k* in Equation (10).

Suppose measurements $\omega_{1},\ldots ,\omega_{n}$ are drawn from a gamma distribution Ω∼G(a,b) but we fit an exponential distribution E(μ) with location (mean) parameter μ>0. The exponential likelihood for a single observation ω is defined as

$$L_{\omega }\left(\mu \right)=f\left(\omega |\mu \right)=\mu^{-1}\exp\left(-\omega /\mu \right),$$

and the corresponding log-likelihood becomes

$$L_{\omega }\left(\mu \right)=\log\left(L_{\omega }\left(\mu \right)\right)=-\log\left(\mu \right)-\omega /\mu .$$

The first and second derivatives of the log-likelihood with respect to μ are

$$\begin{array}{cc} {\dot{L}}_{\omega }\left(\mu \right) & =\frac{d}{d\mu }L_{\omega }\left(\mu \right)=-\mu^{-1}+\omega \mu^{-2}\\ {\ddot{L}}_{\omega }\left(\mu \right) & =\frac{d}{d\mu }{\dot{L}}_{\omega }\left(\mu \right)=\mu^{-2}-2\omega \mu^{-3}. \end{array}$$

The sensitivity matrix (scalar, since μ is univariate) is defined as

(A4) $$\begin{array}{cc} A_{*} & =E_{\omega }\left[-{\ddot{L}}_{\omega }\left(\mu \right)\right]=E_{\omega }\left[-\mu^{-2}+2\omega \mu^{-3}\right]=2E_{\omega }\left[\omega \right]\mu^{-3}-\mu^{-2}, \end{array}$$

and the variability matrix (also a scalar here) is

(A5) $$\begin{array}{cc} B_{*} & =E_{\omega }\left[{\dot{L}}_{n}\left(\mu \right){\dot{L}}_{n}{\left(\mu \right)}^{⊤}\right]=E_{\omega }\left[\left(-\mu^{-1}+\omega \mu^{-2}\right){\left(-\mu^{-1}+\omega \mu^{-2}\right)}^{⊤}\right]\\ \; & =E_{\omega }\left[\mu^{-2}+\omega^{2}\mu^{-4}-2\omega \mu^{-3}\right]\\ \; & =\mu^{-2}-2E_{\omega }\left[\omega \right]\mu^{-3}+E_{\omega }\left[\omega^{2}\right]\mu^{-4}. \end{array}$$

### Appendix D.1. Correct Model Specification

If the data ω are drawn from E(μ), then the known moment identities apply:

(A6) $$\begin{array}{cc} \; & E_{\omega }\left[\omega \right]=\mu \;\mathrm{and}\;E_{\omega }\left[\omega^{2}\right]=2\mu^{2}. \end{array}$$

Substituting in Equations (A4) and (A5) yields

$$\begin{array}{cc} A_{*} & =-\mu^{-2}+2\mu^{-3}\mu =\mu^{-2}\\ B_{*} & =\mu^{-2}-2\mu \mu^{-3}+2\mu^{2}\mu^{-4}=\mu^{-2}. \end{array}$$

Hence, under correct model specification, we have $A_{*}=B_{*}$ for any $\mu \in R_{+}$, implying that the naive and sandwich variance estimators coincide and both equal $\mu^{2}/n$.

### Appendix D.2. Incorrect Model Specification

If Ω≁E(λ), the identities in (A6) no longer hold. Instead, we define

$$\begin{array}{cc} \; & E_{\omega }\left[\omega \right]=m_{\omega }\;\mathrm{and}\;E_{\omega }\left[\omega^{2}\right]=\mathrm{Var}\left[\omega \right]+E{\left[\omega \right]}^{2}=s_{\omega }^{2}+m_{\omega }^{2}, \end{array}$$

where $m_{\omega }$ and $s_{\omega }^{2}$ are the sample mean and variance of $\omega_{1},\ldots ,\omega_{n}$, respectively. Substituting in the sensitivity and variability matrices of Equations (A4) and (A5) gives

(A7) $$\begin{array}{cc} A_{n} & =2m_{\omega }\mu^{-3}-\mu^{-2}\\ B_{n} & =\mu^{-2}-2m_{\omega }\mu^{-3}+\left(s_{\omega }^{2}+m_{\omega }^{2}\right)\mu^{-4}. \end{array}$$

Let $\hat{\mu }$ be the maximum likelihood estimator of μ, and define the sample statistics

$$\begin{array}{c} m_{\omega }=\frac{1}{n}\sum_{t=1}^{n}\omega_{t},\;s_{\omega }^{2}=\frac{1}{n-1}\sum_{t=1}^{n}{\left(\omega_{t}-m_{\omega }\right)}^{2}. \end{array}$$

The naive variance estimator then becomes

(A8) $$\begin{array}{cc} \Sigma_{n}^{\mathrm{naive}} & =\frac{1}{n}A_{n}^{-1}\\ \; & ={\left(2m_{\omega }{\hat{\mu }}^{-3}-{\hat{\mu }}^{-2}\right)}^{-1}/n\\ \; & ={\hat{\mu }}^{3}{\left(2m_{\omega }-\hat{\mu }\right)}^{-1}/n, \end{array}$$

and the sandwich variance estimator is

(A9) Σnsand=1nAn−1Bn−1An−1=μ^3(2mω−μ^)−1μ^−2−2mωμ^−3+(sω2+m^ω2)μ^−4μ^3(2mω−μ^)−1/n=μ^4(2mω−μ^)−21−2mωμ^−1+(sω2+m^ω2)μ^−2/n,

The maximum likelihood estimate of μ can be derived from the full log-likelihood

(A10) $$\begin{array}{cc} L_{n}\left(\mu \right) & =\sum_{t=1}^{n}L_{\omega_{t}}\left(\mu \right)=-n\log\left(\mu \right)-\mu^{-1}\sum_{t=1}^{n}\omega_{t}, \end{array}$$

by setting its derivative to zero

$$\begin{array}{cc} \frac{d}{d\mu }L_{n}\left(\mu \right) & =\frac{d}{d\mu }\left(-n\log\left(\mu \right)-\mu^{-1}n\;\cdot \;m_{\omega }\right)=0. \end{array}$$

This results in the following expression for $\hat{\mu }$

$$\begin{array}{cc} \; & -n{\hat{\mu }}^{-1}+n\;\cdot \;m_{\omega }{\hat{\mu }}^{-2}=0, \end{array}$$

from which it follows that the ML value of $\hat{\mu }=m_{\omega }$, the sample mean of the data.

Substituting $\hat{\mu }=m_{\omega }$ into Equations (A8) and (A9) yields the following estimators of the naive and sandwich variances

(A11) $$\Sigma_{n}=\left\{\begin{array}{cc} \Sigma_{n}^{\mathrm{naive}}=m_{\omega }^{2}/n,\; & \mathrm{naive}\;\mathrm{variance},\\ \Sigma_{n}^{\mathrm{sand}}=s_{\omega }^{2}/n,\; & \mathrm{sandwich}\;\mathrm{variance}. \end{array}\right.$$

Since the *n* scores ${\dot{L}}_{\omega_{1}}\left(\hat{\mu }\right),\ldots ,{\dot{L}}_{\omega_{n}}\left(\hat{\mu }\right)$ are independent, the variability matrix $B_{n}$ in Equation (A7) is not affected by serial dependence. Accordingly, no autocorrelation adjustment such as the Newey and West [61] correction in Equation (27) is required.

The *omnibus* scalar *k* for $\hat{\mu }=m_{\omega }$ is now equal to

(A12) $$k=\frac{d}{\mathrm{tr}\left({\left(\Sigma_{n}^{\mathrm{naive}}\right)}^{-1}\Sigma_{n}^{\mathrm{sand}}\right)}=\frac{1}{{\left(m_{\omega }^{2}/n\right)}^{-1}\left(s_{\omega }^{2}/n\right)}=m_{\omega }^{2}s_{\omega }^{-2}.$$

### Appendix D.3. Population Quantities

If we would know that the ω’s are drawn from a gamma distribution G(a,b), then

$$\begin{array}{cc} \; & E_{\omega }\left[\omega \right]=ab\;\mathrm{and}\;\mathrm{Var}\left[\omega \right]=ab^{2}. \end{array}$$

We can substitute these expressions into the sensitivity and variability matrices of Equation (A11). This would give the following expressions for the naive and sandwich variances

(A13) $$\Sigma =\left\{\begin{array}{cc} \;a^{2}b^{2}/n & \mathrm{naive}\;\mathrm{variance},\\ \;ab^{2}/n & \mathrm{sandwich}\;\mathrm{variance}. \end{array}\right.$$

This demonstrates that the sandwich variance can be either larger or smaller than the naive variance. Specifically, if a<1 the sandwich variance exceeds the naive variance, whereas for 0<a<1 it yields smaller confidence intervals for $\hat{\mu }$. For a=1, the two estimators coincide. This is intuitive, because setting a=1 in the gamma PDF $f_{G}\left(\omega |a,b\right)$ of Equation (28) recovers the exponential density $f_{E}\left(\omega |\mu \right)$ with b=μ.

We can also derive an expression for the theoretic *omnibus* scalar. Indeed, we can write

(A14) $$k=\frac{d}{\mathrm{tr}\left({\left(\Sigma^{\mathrm{naive}}\right)}^{-1}\Sigma^{\mathrm{sand}}\right)}=\frac{1}{{\left(a^{2}b^{2}/n\right)}^{-1}\left(ab^{2}/n\right)}=a.$$

And, thus, the theoretic *omnibus* scalar is simply equal to *a*, the shape parameter of the gamma distribution. This confirms the relationship, $\Sigma^{\mathrm{sand}}=a^{-1}\Sigma^{\mathrm{naive}}$.

### Appendix D.4. Computer Implementation

We implement the naive and sandwich variance estimators of Equation (A11) in Matlab. We also compute credible intervals of $\hat{\mu }$ using OFS adjustment and magnitude-, curvature- and sandwich-adjusted MCMC simulation using the Random Walk Metropolis algorithm. The script below computes Table 3 and Table 4 of this paper. Built-in functions are highlighted with a low dash.

## Appendix E

The Xinanjiang conceptual watershed model is the result of decades of work by Dr. Renjun Zhao and his colleagues at the Hydrological Bureau of the Ministry of Water Resources in China. The model’s initial formulation, based on a saturation-excess runoff mechanism and a top-down runoff generation approach, was developed in 1963 [65]. In 1980, it was formally named the Xinanjiang model [66], reflecting its intended application to the humid Xinanjiang river basin in China [94]. In a second development phase (1980–2002), several structural improvements were made, including a three-layer evapotranspiration module, the introduction of interflow as a runoff component, and the replacement of the original hydrograph method with a linear reservoir and/or lag-routing techniques.

The Xinanjiang model transforms areal average precipitation into streamflow by modeling control volumes, state variables, and fluxes as outlined in Figure A1.

The Xinanjiang model is driven by daily time series of areal-average rainfall, ${\left(p_{1},\ldots ,p_{n}\right)}^{⊤}$, and potential evapotranspiration, ${\left(e_{p1},\ldots ,e_{pn}\right)}^{⊤}$. Our implementation follows the formulations of Zhao [94] and Jayawardena and Zhou [75], as summarized in ODE form by Knoben et al. [76], but includes two key additions: (i) an adjustment coefficient, $f_{c}$, to convert meteorological estimates of potential evapotranspiration, $e_{p}$ (mm/d), into local estimates of actual evaporation; and (ii) a cascade of three linear reservoirs to route channel inflow and convert it into river discharge, *q* (mm/d).

Table A1 summarizes the state variables and fluxes associated with the control volumes of the Xinanjiang model, along with their corresponding symbols and units.

**Table A1:** State variables and fluxes of the Xinanjiang model.

	Symbol	Description	Units
State	*w*	Tension water storage in upper soil layer	mm
	$s_{f}$	Free water storage in upper soil layer	mm
	$s_{i}$	Interflow reservoir	mm
	$s_{g}$	Groundwater reservoir	mm
	$s_{r}^{m}$	Water storage in cascade of routing reservoirs; m=1,…,3	mm
Fluxes into/out of compartments	$p_{t}$	Precipitation	mm d^−1^
	$e_{p}$	Potential evapotranspiration	mm d^−1^
	$e_{\mathrm{pan}}$	Pan evaporation	mm d^−1^
	$p_{i}$	Infiltration	mm d^−1^
	$r_{b}$	Direct runoff from impermeable area	mm d^−1^
	*r*	Runoff from tension water	mm d^−1^
	$r_{s}$	Surface runoff	mm d^−1^
	$r_{i}$	Interflow	mm d^−1^
	$r_{g}$	Baseflow	mm d^−1^
	$q_{s}$	Surface runoff	mm d^−1^
	$q_{i}$	Delayed interflow	mm d^−1^
	$q_{g}$	Delayed baseflow	mm d^−1^
	$q_{\mathrm{ch}}$	Channel inflow	mm d^−1^
	$q_{t}$	River discharge	mm d^−1^

A mass-conservative, second-order integration method with adaptive time stepping is used to solve for the state variables *w*, $s_{f}$, $s_{i}$, $s_{g}$, $s_{r}^{1}$, $s_{r}^{2}$, and $s_{r}^{3}$, as well as the fluxes $r_{x}$ and $q_{x}$ into and out of the seven control volumes. A spin-up period is applied to minimize the influence of initial state conditions.

The fourteen parameters of the Xinanjiang model are listed in Table A2.

**Table A2:** Description of Xinanjiang parameters, including symbols, units, lower and upper bounds.

Symbol	Description	Units	Min.	Max.
$f_{p}$	Ratio of potential evapotranspiration to pan evaporation	-	0.5	1.5
$A_{\mathrm{im}}$	Impervious area	-	$10^{-4}$	$10^{-1}$
*a*	Tension water distribution inflection parameter	-	−0.5	0.5
*b*	Tension water distribution shape parameter	-	$10^{-1}$	2
$f_{\mathrm{wm}}$	Fraction of $s_{\mathrm{tot}}$ that is $w_{\max}$	-	$10^{-3}$	1
$f_{\mathrm{lm}}$	Fraction of $W_{\max}$ that is tension water threshold for evaporation change	-	$10^{-3}$	1
*c*	Fraction of tension water threshold for second evaporation change	-	$10^{-3}$	1
$s_{\mathrm{tot}}$	Total soil moisture storage	mm	1	$10^{3}$
β	Free water distribution shape parameter	-	$10^{-3}$	2
$k_{i}$	Free water interflow parameter	d^−1^	$10^{-3}$	3
$k_{g}$	Free water groundwater parameter	d^−1^	$10^{-3}$	1
$c_{i}$	Interflow time coefficient	d^−1^	$10^{-3}$	1
$c_{g}$	Baseflow time coefficient	d^−1^	$10^{-3}$	1
$k_{f}$	Recession constant of routing reservoirs	d^−1^	$10^{-1}$	5

The minimum and maximum values of the model parameters are collected in the d×1 vectors $\theta^{\min}$ and $\theta^{\max}$, respectively, where individual entries are denoted by $\theta_{j}^{\min}$ and $\theta_{j}^{\max}$ for all j=1,…,d. This concludes the description of the Xinanjiang model.

## Appendix F

This Appendix presents the correlation matrix of the naive posterior samples of the Xinanjiang model parameters and nuisance variables ν and ξ using the historical record of discharge measurements and Student *t* log-likelihood function of Equation (31).

**Table A3:** MCMC-derived naive posterior correlation matrix of the Xinanjiang model parameters and ν and ξ using the Student *t* likelihood.

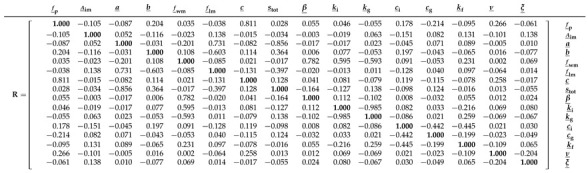

## Appendix G

In this Appendix, we compare the frequentist bread matrix $A_{n}^{s}$ against its sample average ${\hat{A}}_{n}^{s}$ derived from Bayesian inference using the naive posterior realizations, ${\hat{A}}_{n}^{s}=\frac{1}{n}\mathrm{Cov}{\left[\left\{\theta_{\left(b\right)},\theta_{\left(b+1\right)},\ldots ,\theta_{\left(T\right)}\right\}\right]}^{-1}$, where the first *b* samples $\theta_{\left(0\right)},\ldots ,\theta_{\left(b-1\right)}$ of the *N* Markov chains are discarded as burn-in.

**Table A4:** Frequentist $A_{n}^{s}$ and Bayesian ${\hat{A}}_{n}^{s}$ estimator of the bread matrix of the Xinanjiang model parameters and nuisance variables ν and ξ for the Student *t* log-likelihood function.

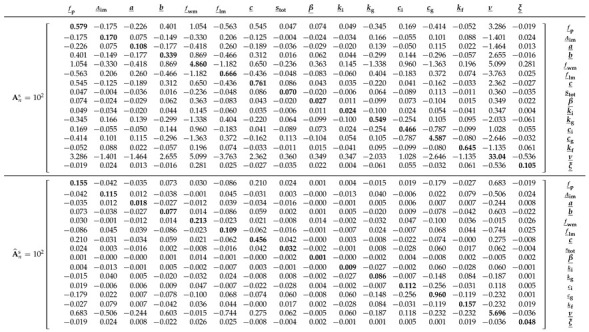

## Appendix H

In this Appendix, we present a scatterplot of the width of the 100γ% Xinanjiang streamflow credible intervals as function of ML simulated discharge. The blue and red dots correspond to the naive and sandwich variance estimators, respectively. The color tints represent different confidence levels.
